# Opening a Pandora’s Flask on a Prototype Catalytic Direct Arylation Reaction of Pentafluorobenzene: The Ag_2_CO_3_/Pd(OAc)_2_/PPh_3_ System

**DOI:** 10.1021/acs.organomet.3c00309

**Published:** 2023-08-30

**Authors:** George M.H. Platt, Pedro M. Aguiar, Gayathri Athavan, Joshua T.W. Bray, Neil W.J. Scott, Ian J.S. Fairlamb, Robin N. Perutz

**Affiliations:** Department of Chemistry, University of York, York YO10 5DD, United Kingdom

## Abstract

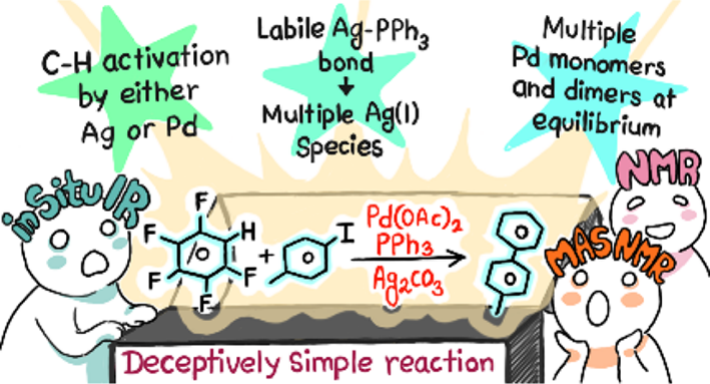

Direct C–H functionalization reactions have opened new avenues in catalysis, removing the need for prefunctionalization of at least one of the substrates. Although C–H functionalization catalyzed by palladium complexes in the presence of a base is generally considered to proceed by the CMD/AMLA-6 mechanism, recent research has shown that silver(I) salts, frequently used as bases, can function as C–H bond activators instead of (or in addition to) palladium(II). In this study, we examine the coupling of pentafluorobenzene **1** to 4-iodotoluene **2a** (and its analogues) to form 4-(pentafluorophenyl)toluene **3a** catalyzed by palladium(II) acetate with the commonplace PPh_3_ ligand, silver carbonate as base, and DMF as solvent. By studying the reaction of **1** with Ag_2_CO_3_/PPh_3_ and with isolated silver (triphenylphosphine) carbonate complexes, we show the formation of C–H activation products containing the Ag(C_6_F_5_)(PPh_3_)_n_ unit. However, analysis is complicated by the lability of the Ag–PPh_3_ bond and the presence of multiple species in the solution. The speciation of palladium(II) is investigated by high-resolution-MAS NMR (chosen for its suitability for suspensions) with a substoichiometric catalyst, demonstrating the formation of an equilibrium mixture of Pd(Ar)(κ^1^-OAc)(PPh_3_)_2_ and [Pd(Ar)(μ-OAc)(PPh_3_)]_2_ as resting states (Ar = Ph, 4-tolyl). These two complexes react stoichiometrically with **1** to form coupling products. The catalytic reaction kinetics is investigated by *in situ* IR spectroscopy revealing a two-term rate law and dependence on [Pd_tot_/nPPh_3_]^0.5^ consistent with the dissociation of an off-cycle palladium dimer. The first term is independent of [**1**], whereas the second term is first order in [**1**]. The observed rates are very similar with Pd(PPh_3_)_4_, Pd(Ph)(κ^1^-OAc)(PPh_3_)_2_, and [Pd(Ph)(μ-OAc)(PPh_3_)]_2_ catalysts. The kinetic isotope effect varied significantly according to conditions. The multiple speciation of both Ag^I^ and Pd^II^ acts as a warning against specifying the catalytic cycles in detail. Moreover, the rapid dynamic interconversion of Ag^I^ species creates a level of complexity that has not been appreciated previously.

## Introduction

Metal-mediated direct C–H bond functionalization reactions have been studied extensively as cost-effective, eco-friendly, and sustainable synthetic chemistry alternatives to conventional cross-coupling reactions, with enhanced atom economy (at least in substrate) and less metal waste.^1^ The strategies are most commonly applied to aryl–aryl bond formation as it avoids the prefunctionalization of aromatics/heteroaromatics with electropositive heteroatoms.^2,3^ A wide range of aromatic hydrocarbons have been shown to undergo C–H bond functionalization reactions in the presence of carboxylates,^4^ and this method has been successfully applied to the functionalization of electron-rich (*e.g.*, indole),^5,6^ neutral (*e.g.*, benzene),^7^ and poor (*e.g.*, pyridine *N*-oxide)^8^ aromatic systems. The direct arylation of a fluoroarene^9−19^ is an example with significant industrial interest for the potential in accessing fluorinated compounds without presynthesized organometallic species.^20,21^ Furthermore, polyfluoroarenes undergo regioselective functionalizations influenced by their electronic and steric properties via the ortho-fluorine effect.^9,22−27^ In this paper, we address a prototype example: the cross-coupling of aryl iodides with pentafluorobenzene catalyzed by palladium acetate with silver carbonate as the added base.

**Scheme 1:**
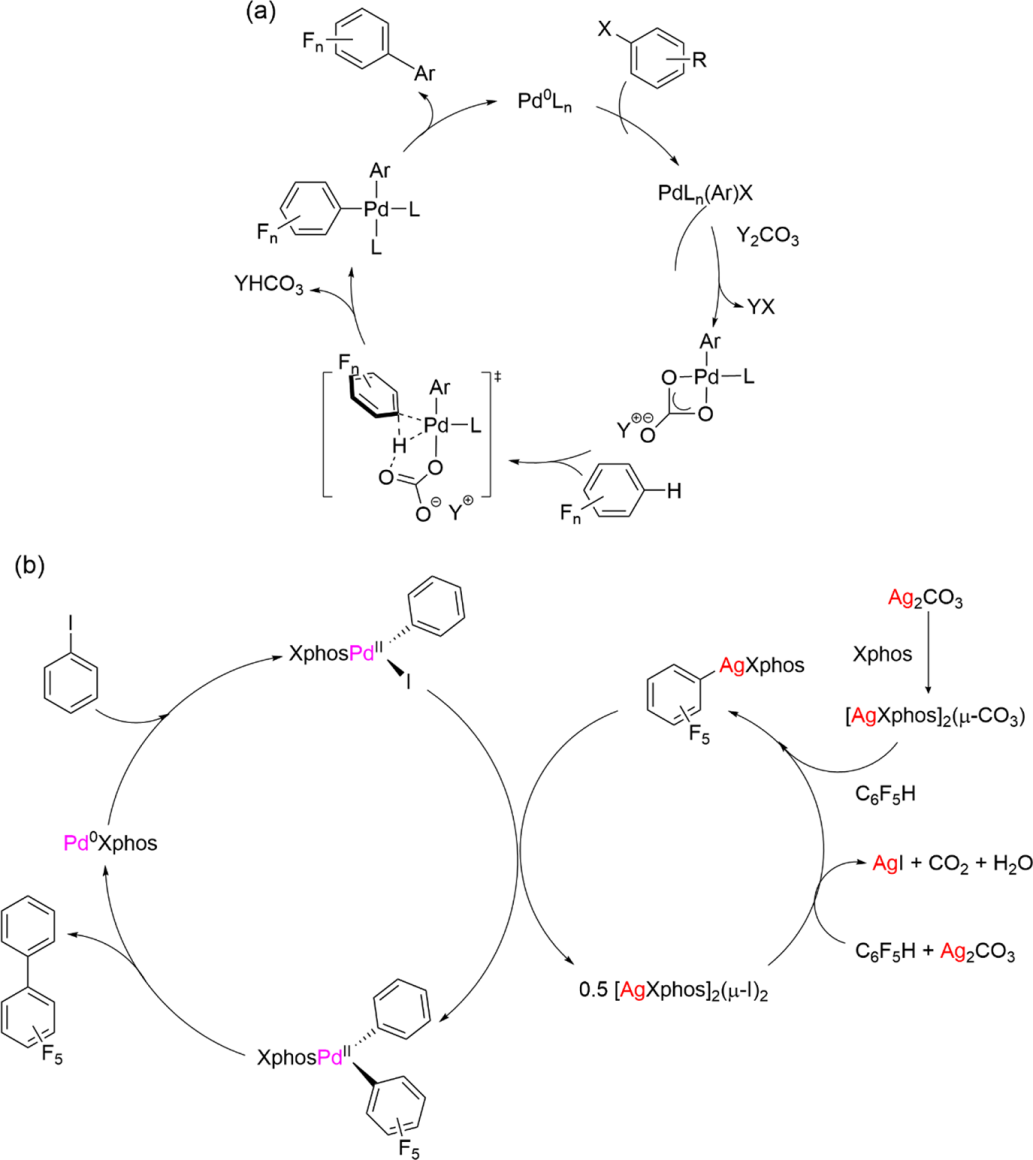
(a) Commonly Proposed CMD/AMLA(6) Mechanism for Direct Arylation of Pentafluorobenzene. (b) Catalytic Cycle with Silver Performing C–H Activation Step (Adapted with Permission from ref (45), Copyright 2022, American Chemical Society)

The catalytic cycle for the direct arylation of polyfluoroarenes has been proposed to involve ambiphilic metal ligand activation (AMLA) or concerted metalation deprotonation (CMD)^28−31^ between an aryl-Pd κ^1^-carboxylate intermediate and the fluoroaromatic reactant (Scheme 1a).^30^ The AMLA(6) transition state is characterized by the agostic interaction of the arene substrate at the same time as the interaction of the arene hydrogen with the carbonyl of the coordinated carboxylate. This mechanism highlights the potential to enhance the reactivity of typically inert bonds by a combination of multiple weak interactions working in synergy; the H-bonding interaction between the C–H bond and the carboxylate ligand increases the electron density on the C–H bond, and the resulting enhancement in the agostic interaction polarizes the C–H bond and increases the acidity of the proton.^30^ The reactivity of isolated metal complexes in stoichiometric reactions has been used as the evidence for proposing catalytic intermediates in the AMLA(6) mechanism. This approach was highlighted by Wakioka and co-workers who reported the stoichiometric reaction of a preformed dinuclear [Pd(Ar)(μ-OAc)(PPh_3_)]_2_ (Ar = Ph, 2-MeC_6_H_4_, 2,6-Me_2_C_6_H_3_) complex with 3-methylthiophene^32^ and benzothiazole.^33^ A mononuclear complex, Pd(Ar)(κ^2^-OAc)(PPh_3_), was proposed as the active catalytic species based on the equilibrium with the dinuclear [Pd(Ar)(μ-OAc)(PPh_3_)]_2_ in solution and the isolation of stable mononuclear [Pd(Ar)(*N*-BT)(κ^1^-OAc)(PPh_3_)] (BT = benzothiazole).

In Scheme 1a, the base is written as Y_2_CO_3_ with no particular role for the cation, Y, other than to remove YX, as highlighted for AgI.^10^ In the frequent situation that the base is silver carbonate or another silver salt, this mechanistic hypothesis has been unraveling. Several publications, notably from the Sanford, Hartwig, and Larrosa groups, have recently demonstrated that silver(I) complexes are active in C–H activation even in the absence of palladium.^34−44^ Evidence comes from the detection of silver aryl complexes, H/D exchange reactions, and study of the kinetics of cross-coupling; a scheme summarizing the evidence may be found in ref (45). The evidence for silver participation has also been reviewed.^46−49^ We recently reported^45^ that the reaction of Ag_2_CO_3_ with XPhos in the presence of pentafluorobenzene yields Ag(C_6_F_5_)(XPhos) and showed that this is a mononuclear complex that is linear at silver. Its ^31^P{^1^H} NMR spectra show characteristic large couplings to ^107^Ag and ^109^Ag (51.8 and 48.2% abundance, respectively). It undergoes ready exchange with free XPhos or its *t*Bu analogue by an associative mechanism on a timescale of a few seconds. This complex reacts stoichiometrically with PdI(C_6_H_5_)(XPhos) to form the cross-coupling product C_6_H_5_-C_6_F_5_. Catalytic cross-coupling can be achieved with 5 mol % Ag(C_6_F_5_)(XPhos) as the sole silver source.^45^ These results led us to propose a catalytic cycle (Scheme 1b) in which the C–H activation step occurs at silver and is followed by transmetalation to palladium prior to C–C bond formation. This cycle may compete with a cycle in which C–H activation occurs at palladium as in Scheme 1a. Although heterobimetallic Ag–Pd species are also plausible intermediates, no experimental evidence has been found to support their involvement.^50,51^

Palladium catalyst speciation, particularly involving Pd(0)L_*n*_ species, is affected by the type of phosphine, solvent, additives, and reaction conditions in general. For Xphos and PPh_3_, there is experimental evidence that Pd(0)L_*n*_ species can form from mixtures of Pd(OAc)_2_/phosphine ligand.^52^ We recognize that dinuclear Pd species (leading to other higher-order Pd clusters)^53^ can also form, as shown by stoichiometric experiments reported by Fairlamb et al. (for PPh_3_), Jutand et al. (for Xphos), and others.^53−55^ For the purposes of this paper, we refer to the active catalyst species being Pd(0)L_*n*_, which is supported by our global findings (see later).

**Scheme 2:**
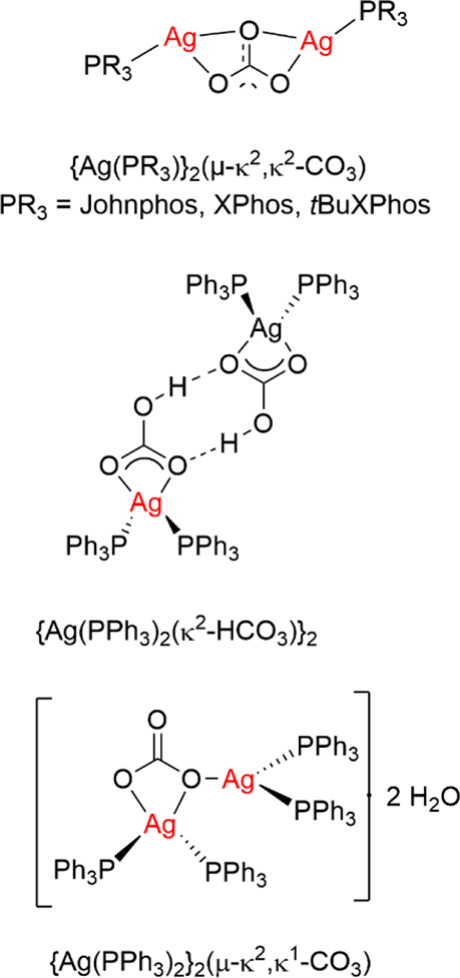
Carbonate and Bicarbonate Complexes of Silver with Phosphine Ligands

Silver carbonate is highly insoluble in solvents used for cross-coupling reactions but reacts with phosphines to form soluble silver phosphine carbonate or bicarbonate complexes. Tlahuext-Aca and Hartwig reported two {Ag(phosphine)}_2_(μ-κ,^2^κ^2^-CO_3_) complexes, one with a P*t*BuXPhos ligand that was characterized crystallographically and one with a Johnphos ligand (Scheme 2).^40^ We reported a direct analogue with XPhos, also characterized crystallographically.^45^ Importantly, {Ag(Johnphos)}_2_(μ-κ,^2^κ^2^-CO_3_) proved to be an active H/D exchange catalyst for thiophene. A related dinuclear complex of silver carbonate with triphenylphosphine {Ag(PPh_3_)_2_}_2_(μ-κ,^2^κ^1^-CO_3_) and a bicarbonate complex {Ag(PPh_3_)_2_(κ^2^-HCO_3_)}_2_ have been described (Scheme 2),^56^ but their reactivity toward fluoroarenes is unknown.

The study of phosphines coordinated to silver is greatly aided by the presence of two I = ^1^/_2_ isotopes of silver (see above). Muetterties and Alegranti showed that Ag(P*p*-tol_3_)_*n*_ species are extremely labile (activation energy for Ag–P rupture in [Ag(P*p*-tol_3_)_4_]NO_3_ is 9 ± 1 kcal/mol). The lability prevents observation of coupling at room temperature, but the coupling constants can be measured from low-temperature spectra, revealing a trend according to the number of phosphine ligands:^57^ [Ag(P*p*-tol_3_)_n_]PF_6_*J*(^107^Ag–P) *n* = 4, 224 Hz; *n* = 3, 321 Hz; *n* = 2, 496 Hz. Subsequent work has confirmed that the coupling constants become larger as the coordination number of silver decreases.^58−61^ At a more detailed level, the value of *J*(Ag–P) is associated with the *s*-character of the hybrid orbitals of Ag and P via the Fermi contact term. The *s* character of the phosphorus orbitals barely varies for different PPh_3_ complexes, but the *s* character at silver is sensitive to the geometry and increases as the P–Ag–P angle increases within a set of complexes with related geometries.^61−63^ In keeping with Muetterties and Alegranti’s early work, later authors have found that PPh_3_ is very labile at silver, so measurements of *J*(Ag–P) typically require low temperatures.

In our previous paper,^45^ we showed that silver carbonate reacts with pentafluorobenzene and PPh_3_ leading to a new ortho-fluorine resonance in the ^19^F NMR and a broad singlet in the ^31^P{^1^H} NMR spectrum with no apparent coupling to ^107^Ag or ^109^Ag at room temperature. However, the ^31^P{^1^H} signal splits into several resonances with conspicuous coupling to Ag nuclei on cooling to −100 °C, indicating the presence of multiple species at equilibrium (Table S8). At the same time, several ^19^F resonances were observed at low temperature around δ −100, characteristic of the ortho-F of Ag(C_6_F_5_) species. The complex speciation contrasted with the corresponding reaction with XPhos that led to a single species, Ag(C_6_F_5_)(XPhos). Our earlier work as well as the related studies relied on the use of more specialist phosphines, leaving it unclear to what extent the results could be generalized. In this paper, we return to the use of the everyday phosphine, PPh_3_, and investigate the effect of different parameters on the palladium-catalyzed cross-coupling reaction of 4-iodotoluene with pentafluorobenzene leading to improved understanding of the optimum reaction conditions. We also investigate the reaction of the carbonate and bicarbonate complexes of Scheme 2 with pentafluorobenzene in the absence of palladium and show that they are capable of C–H bond activation, albeit with complex speciation.

Instead of following the reaction by conventional solution NMR spectroscopy that is hampered by the suspensions formed with insoluble salts, we adopted two different techniques: high-resolution magic angle spinning (HR-MAS) NMR spectroscopy and *in situ* IR spectroscopy. HR-MAS NMR has been applied for analysis of biological samples and structural study on heterogeneous catalysts in suspension,^64−68^ including real-time reaction monitoring.^69−71^ We observed several Pd intermediates involved in the catalytic direct arylation reaction of pentafluorobenzene by HR-MAS NMR spectroscopy and confirmed their identity by *ex situ* MS analysis. These species were also studied in stoichiometric reactions to discriminate between the key catalytic intermediates. *In situ* IR spectroscopy allows the catalytic reaction to be monitored in a stirred, temperature-controlled flask attached to a Schlenk line, revealing that the kinetics requires a two-term rate law and that the kinetic isotope effect varies with conditions. Our study shows that (a) the reaction with PPh_3_ is complicated by multiple speciation with both silver and palladium coordination; (b) Ag(I) plays a direct role in C–H bond activation; and (c) there are two very different pathways for catalytic reaction, resulting in unusual kinetic behavior.

## Results

### Effect of Reaction Conditions on Catalytic Conversion to Biaryls

The direct arylation of 4-iodotoluene **2a** with pentafluorobenzene **1** was selected as the model reaction system (Reactions R1).^72^ Quantitative conversion of substrate **2a** was observed, and the product 4-(pentafluorophenyl)toluene **3a** was isolated in 84% yield after purification. Conversion of **2a** after 24 h was determined from the integration of the methyl ^1^H signals of reagent **2a** and product **3a** at δ 2.31 and 2.44, respectively. The homocoupling side-product 4,4′-dimethyl-1,1′-biphenyl was formed in trace quantities for the reaction of **2a** with over 10 mol % of Pd catalyst loading or the reaction in the absence of the Ag^I^ additive but was otherwise absent. The methyl protons of this biaryl side-product were observed at δ 2.40 and did not interfere with the analysis.


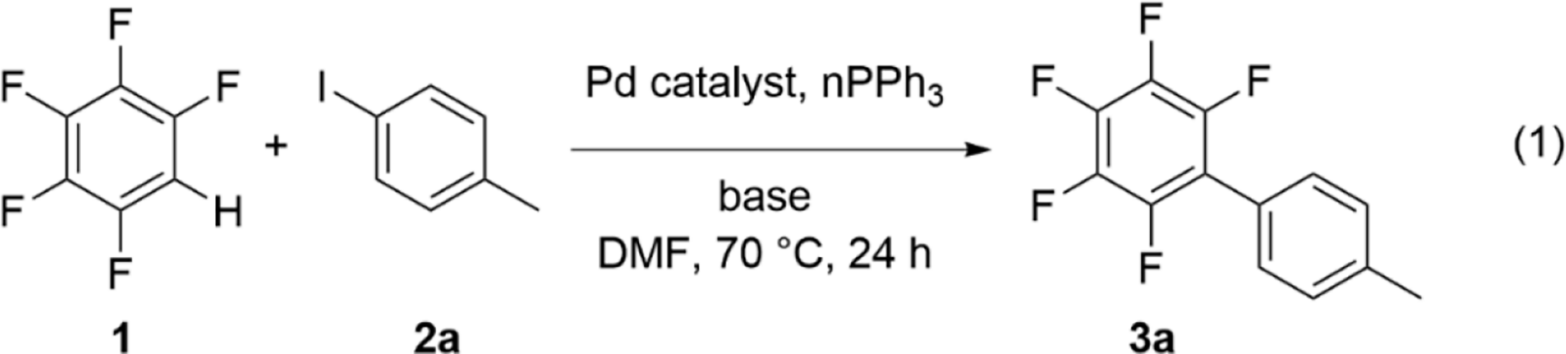
R1

The role of the acetate ligands on the precatalyst Pd(OAc)_2_ (we abbreviate high-purity nitrite-free Pd_3_(OAc)_6_ to Pd(OAc)_2_ throughout the paper) was studied by comparing the yields of biaryl product **3a** for reactions using Pd(PPh_3_)_4_, Pd(PPh_3_)_2_Cl_2_, and PdCl_2_ precatalysts, in each case with 0.75 equiv Ag_2_CO_3_ (Table 1). Results comparable with Pd(OAc)_2_ + 10 mol % PPh_3_ (100% NMR yield, 84% isolated yield) were obtained for reactions catalyzed by Pd(PPh_3_)_4_ and Pd(PPh_3_)_2_Cl_2_. The ligand PPh_3_, particularly in excess greater than 3, is understood to play a role in both the reduction of Pd^II^ to Pd^0^ and the stabilization of the oxidative addition complex in stoichiometric studies.^73^

**Table 1:** Conversions of **2a** and Yields of Isolated Product **3a**

entry	catalyst^a^	Ag_2_CO_3_ (equiv)	PPh_3_ (mol %)	% conversion^a^ (% yield)^b^
				
1	5 mol % Pd(OAc)_2_	0.75	10	quant. (84)
2	5 mol % Pd(PPh_3_)_4_	0.75	0	quant. (85)
3	5 mol % Pd(PPh_3_)_2_Cl_2_	0.75	10	quant. (87)
4	5 mol % PdCl_2_	0.75	10	72 (61)
5	5 mol % Pd(PPh_3_)_2_Cl_2_,	0.75	0	60 (57)
7	1.5 mol % Pd(OAc)_2_	0	3	0
8	1.5 mol % PdCl_2_	0	3	0
9	20 mol % Pd(OAc)_2_	0	40	(14)
10	20 mol % PdCl_2_	0	40	0
11	20 mol % Pd(PPh_3_)_4_	0	0	0

a Standard reaction time 24 h. Determined from integration of methyl ^1^H NMR peaks of the reagent **2a** and the product **3a**.

b After purification by flash chromatography.

We also investigated the effect of changing the base on the catalytic reaction (Table S1). Of the metal carboxylates tested, Ag_2_CO_3_ resulted in the highest conversion of substrate **2a** and yield of the isolated product **3a**. Similar yields were obtained with Pd(OAc)_2_ + 10 mol % PPh_3_ + 1.5 equiv [Me_4_N][OAc] or with Pd(OAc)_2_ + 10 mol % PPh_3_ + 0.75 equiv Ag_2_O. Changing the base to any of K_2_CO_3_, Cs_2_CO_3_, AgOAc, or [Bu_4_N][OAc] resulted in marked reductions in yields. A recent paper reports H/D exchange of pentafluorobenzene with 10 mol % Cs_2_CO_3_ in *d*_6_-acetone or *d*_6_-DMSO.^74^ Some of the action of bases that we observe may be caused by deprotonation of C_6_F_5_H. Nevertheless, only [Me_4_N]OAc and Ag_2_CO_3_ give full conversion in the catalytic reaction.

Alternative solvents utilized in the literature for C–H bond functionalization reactions include dialkyl carbonates, PEG, and water.^75^ The polar aprotic solvents such as dimethylacetamide (DMAc) and *N*-methyl 2-pyrrolidone (NMP) resulted in comparable yields to DMF (Table S2). Ethylene carbonate and propylene carbonate afforded **3a** in reasonable yields.

A variety of monophosphines were tested for comparison to PPh_3_ (Table S3). Of these, P(4-FC_6_H_4_)_3_, P(3,5-(CF_3_)_2_C_6_H_3_)_3_, and P(2-furyl)_3_ were as effective as PPh_3_. Substitution with methoxy groups proved deleterious, whereas PCy_3_ was very poor indeed.

Under the standard conditions, the reaction solution remains clear until the reaction is ca. 70% complete, but subsequently, the solution darkens, suggesting the formation of Pd metal (or possibly Ag). The use of preformed stabilized Pd nanoparticles (Pd-NPs) as an alternative to Pd(OAc)_2_ precatalyst was therefore examined to establish the type of catalyst (homogeneous and/or heterogeneous) involved in the reaction, recognizing that stabilized PdNPs are usually less active than naked, polar aprotic solvent stabilized PdNPs.^76,77^ The catalytic activities of Pd-NPs supported on polyvinylpyrrolidone (PVP) of different polymer weights and particle sizes were tested (Table S4) while maintaining the molar quantity of the Pd at 5 mol %. The reaction at 70 °C required PPh_3_, and the isolated yields were significantly lower than when using Pd(OAc)_2_ precatalyst.

The structure–reactivity relationship between the electronic properties of the fluoroarenes and the reaction rates was studied to characterize the transition state based on a modified Hammett equation. The electronic properties of fluoroarenes were tuned by varying the substituents on the C1 position of 2,3,5,6-tetrafluorobenzene.^78^ Functional groups with electronic properties ranging from electron-donating dimethylamino to electron-withdrawing trifluoromethyl were selected. The reactions of these fluoroarenes achieved quantitative conversion of the starting material to the desired products after 20 h at 70 °C. The substituent effect was determined from relative yields obtained by competition reaction between 4-iodotoluene with 10 equiv each of the 1-X-2,3,5,6-tetrafluorobenzene (X = NMe_2_, OMe, F, Cl, and CF_3_) and pentafluorobenzene analyzed by ^19^F NMR (eq 2).


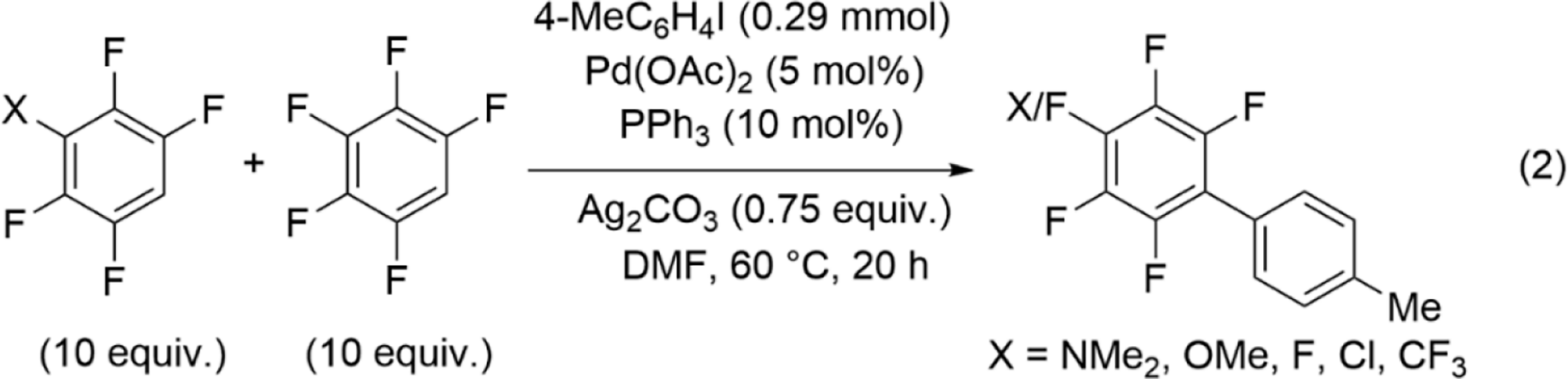
R2

**Figure 1:**
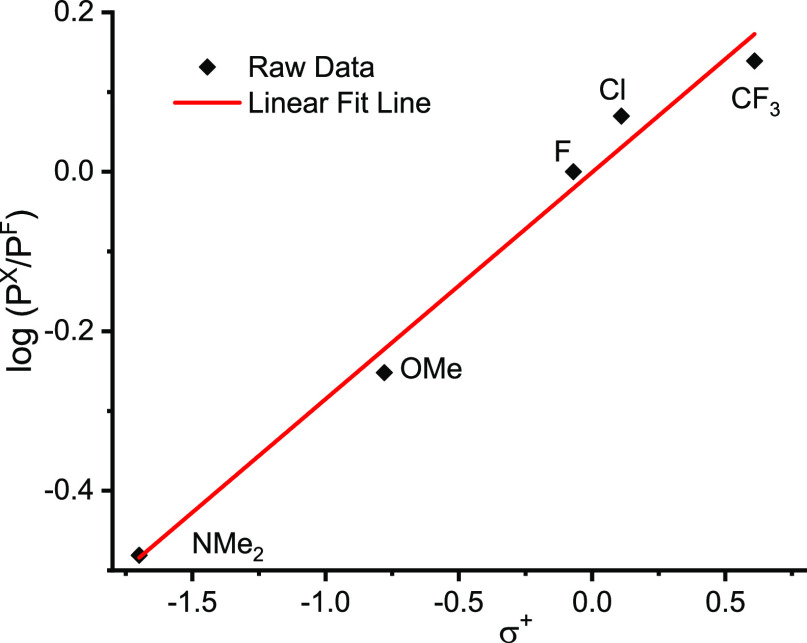
Hammett plot of the competition reaction shown in Reaction R2.

The Hammett equation was modified by substitution of the relative reaction rates represented by the ratio of the two product concentrations (*P*^X^/*P*^F^) with the standard set as X = F instead of X = H (eq 1).^79^ Modest increases in yields were achieved with more electron-withdrawing functional group in the C1 position (Table S5). A linear free-energy relationship (LFER) was observed with the logarithms of the relative yields directly proportional to σ^+^ (Figure 1) yielding *R*^2^ of 0.954. The reaction constant (ρ) determined from the slope of the LFER was +0.28 ± 0.02, consistent with the AMLA(6) pathway that is expected to favor electron-withdrawing substituents stabilizing the negative charge at the TS.^80^
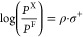
1where *P*^X^ = integration of 1-X-2,3,5,6-tetrafluorobiaryl, *P*^F^ = integration of pentafluorobiaryl, ρ = reaction constant, and σ^+^ = substituent constant.

### Ag Speciation and Possible Intermediates

Following the reports of C–H activation by silver salts, we investigated H/D exchange between pentafluorobenzene and D_2_O catalyzed by Ag(I) salts, monitoring the reaction by ^19^F NMR spectroscopy. Replacement of H by D causes an isotopic shift of the ortho-fluorine resonance of +0.3 ppm and loss of F–H coupling. The exchange with 1 equiv D_2_O in DMF at 50 °C catalyzed by Ag_2_CO_3_ (10 mol %) and PPh_3_ (20 mol %) resulted in 99% deuteration (Reaction R3). When the triphenylphosphine was omitted, the reaction resulted in 51% deuteration, whereas the corresponding reaction using Ag(PPh_3_)_2_(κ^2^-OAc) (see below) in place of Ag_2_CO_3_ + PPh_3_ yielded 37% deuteration.


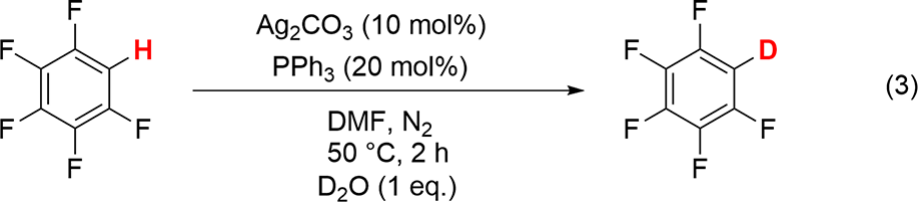
R3

When AgOAc (1 equiv) and PPh_3_ (2 equiv) were reacted with pentafluorobenzene (10 equiv), no Ag(C_6_F_5_)-containing product was detected by ^19^F{^1^H} NMR spectroscopy. Replacement of AgOAc by Ag_2_CO_3_ (1 equiv) resulted in the detection of an Ag(C_6_F_5_)-containing product with ortho-F resonances centered at δ −106.5 (in CH_3_CN). The reaction of AgOAc (1 equiv) or Ag_2_CO_3_ (1 equiv) with C_6_F_5_H (10 equiv) in the absence of phosphine did not generate any detectable Ag(C_6_F_5_) complex, indicating that the PPh_3_ ligand is critical for C–H activation (Table S6). To investigate this reactivity further, the PPh_3_-coordinated Ag carbonate complexes in Scheme 2 were synthesized following literature methodologies,^56^ together with Ag(PPh_3_)_2_(κ^2^-OAc).^81^

**Figure 2:**
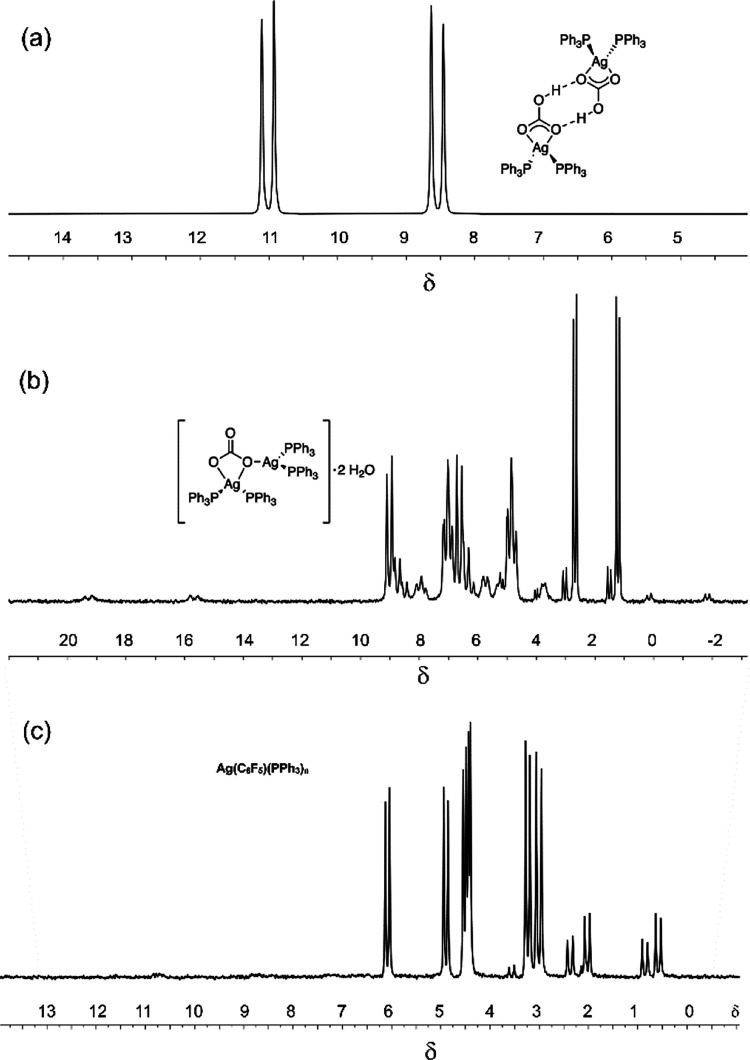
Low-temperature ^31^P{^1^H} NMR spectrum in toluene/dichloromethane (20:80) of (a) [Ag(PPh_3_)_2_(κ^2^-HCO_3_)]_2_ at −80 °C, (b) [Ag(PPh_3_)_2_]_2_CO_3_·2H_2_O at −100 °C, and (c) Ag(C_6_F_5_)(PPh_3_)_n_ at −100 °C.

The Ag–PPh_3_ bond was found to be highly labile at room temperature, resulting in a singlet at room temperature in the ^31^P{^1^H} NMR spectrum of these complexes. The complexes {Ag(PPh_3_)_2_(κ^2^-HCO_3_)}_2_ and {Ag(PPh_3_)_2_}_2_(μ-κ,^2^κ^1^-CO_3_) were characterized using low-temperature ^31^P{^1^H} NMR spectroscopy. The {Ag(PPh_3_)_2_(κ^2^-HCO_3_)}_2_ complex existed as a well-defined species in solution, and the ^31^P{^1^H} NMR spectrum was fully resolved at −80 °C to reveal a pair of overlapping doublets centered at δ 9.78 with coupling constants ^1^*J*_^31^P –^107^Ag_ = 465 Hz and ^1^*J*_^31^P –^109^Ag_ = 537 Hz (Figure 2a). These values may be compared with those for [Ag(XPhos)]_2_(CO_3_) of 634 and 731 Hz; the reduction in the PPh_3_ complex relative to the XPhos complex reflects the increased coordination number of Ag.^57−60^ In contrast, the low-temperature ^31^P{^1^H} NMR showed that {Ag(PPh_3_)_2_}_2_(μ-κ,^2^κ^1^-CO_3_) exists in equilibrium with multiple Ag–PPh_3_ containing species. Most of the resonances appeared as overlapping doublets with characteristic Ag–P coupling; the chemical shifts and coupling constants of the major species could be identified clearly, but other peaks were difficult to distinguish due to peak overlap (Figure 2b, Table S9). The distinguishable coupling constants varied from 317 to 778 Hz for ^109^Ag, indicating the presence of species with a wide range of coordination numbers. Thus, the published crystal structure^56^ represents one of many different species that are formed in solution. Because carbonate can act as a bridging ligand in several different ways,^82^ we can envisage numerous different oligomeric structures.

The phosphine-coordinated Ag complexes were reacted with C_6_F_5_H (10 equiv) to examine the C–H activation capabilities of these complexes. The presence of a C–H activation product, Ag(C_6_F_5_)(PPh_3_)_n_, was detected by the ortho-F resonance at δ −106 using ^19^F{^1^H} NMR spectroscopy with [Ag(PPh_3_)_2_]_2_CO_3_·2H_2_O. Because the structure of the resulting Ag(C_6_F_5_)(PPh_3_)_n_ complex is poorly defined, percent conversions could not be measured accurately. Only when Cs_2_CO_3_ is added is a reaction between {Ag(PPh_3_)_2_(κ^2^-HCO_3_)}_2_ and C_6_F_5_H observed, either at 60 °C or at room temperature (Table 2). We also tested Ag(PPh_3_)_2_(κ^2^-OAc) for reaction with C_6_F_5_H but found no evidence for C–H activation. These experiments show that the carbonate complex [Ag(PPh_3_)_2_]_2_CO_3_·2H_2_O is capable of C–H bond activation but not the bicarbonate complex or the acetate complex.

**Table 2:**
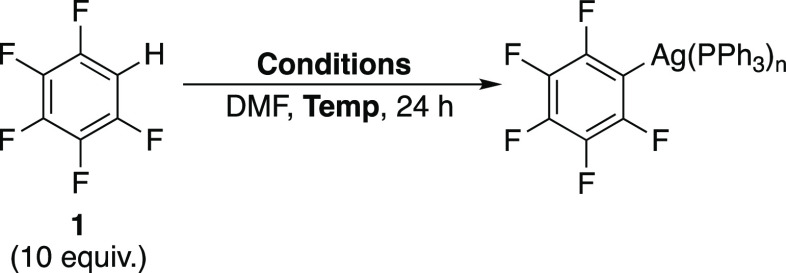
Stoichiometric Reaction of PPh_3_-Coordinated Ag Complexes and C_6_F_5_H^a^


entry	conditions	temperature (°C)	detection of Ag(C_6_F_5_) product^b^
1	[Ag(PPh_3_)_2_(κ^2^-HCO_3_)]_2_ (0.5 equiv)	60	no
2	[Ag(PPh_3_)_2_(κ^2^-HCO_3_)]_2_ (0.5 equiv) + Cs_2_CO_3_ (1 equiv)	60	yes
3	[Ag(PPh_3_)_2_]_2_CO_3_·2H_2_O	60	yes
4	[Ag(PPh_3_)_2_]_2_CO_3_·2H_2_O	RT	yes
5	Ag(PPh_3_)_2_(κ^2^-OAc) (1 equiv)	60	no

a 10 equiv C_6_F_5_H.

b Detected by the ortho-F resonances at δ −106.0.

To study Ag(C_6_F_5_)(PPh_3_)_n_ further, an authentic sample of Ag(C_6_F_5_) was prepared by literature methods^83^ and then reacted with PPh_3_ (2 equiv) in acetonitrile (Reaction R4). The resulting product was isolated from solution as brown crystals and characterized by low-temperature NMR spectroscopy (Figure 2c). The ^31^P{^1^H} NMR spectrum showed five major pairs of Ag-coupled doublets and one minor pair (Table S10). Considering the tetrameric structure of several silver aryl complexes^84−86^ and the infinite chain structure of Ag(C_6_F_5_)(EtCN)_2_,^83^ the presence of multiple species at low temperature may indicate both variable numbers of coordinated phosphines and formation of different oligomers.


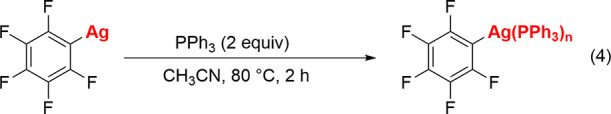
R4

The complex and multiple speciation demonstrated first by the low-temperature spectra of {Ag(PPh_3_)_2_}_2_(μ-κ,^2^κ^1^-CO_3_) and second by the reaction of Ag(C_6_F_5_) with PPh_3_ is reminiscent of the spectrum obtained by the reaction of Ag_2_CO_3_ with PPh_3_ and C_6_F_5_H,^45^ suggesting that there may be species in common. Comparison of the spectra (Tables S8–S10) shows three sets of resonances that are sufficiently close in coupling constant and chemical shift to belong to the same species for all three spectra (Table S11). Quoting the values for {Ag(PPh_3_)_2_}_2_(μ-κ,^2^κ^1^-CO_3_), the first two are at δ 2.0 (*J* 275, 317 Hz) and δ 2.3 (*J* 285, 329 Hz). Literature data for [Ag(PPh_3_)_3_X] (X = BF_4_, Cl, I with *J*(^107^Ag–P) 318, 227, and 262 Hz, respectively)^61^ and for [Ag(PPh_3_)_4_][BF_4_] *J*(av) = 238 Hz^63^ (hence, *J*(^107^Ag–P) = 221 Hz) suggest that the species we observe close to δ 2.0 are most likely of the type [Ag(PPh_3_)_3_X]. There is one further species common to {Ag(PPh_3_)_2_}_2_(μ-κ,^2^κ^1^-CO_3_) and Ag_2_CO_3_/PPh_3_/C_6_F_5_H at δ 17.5 (*J* 677, 778 Hz) that may be due to [Ag(PPh_3_)]_2_(CO_3_) or similar species.

It seemed likely that the amount of silver salt could be reduced to catalytic quantities if an alternative stoichiometric base was employed. The catalytic abilities of silver carbonate and the Ag-carbonate triphenylphosphine complexes were tested employing Cs_2_CO_3_ (0.75 equiv) as the base in each reaction (Table 3). When Cs_2_CO_3_ is used alone for the standard catalytic direct arylation reaction, the conversion after heating for 23 h is 69%. Trace amounts of side-product were also detected in the ^1^H NMR spectrum. When 5 mol % of either [Ag(PPh_3_)_2_(κ^2^-HCO_3_)]_2_ or {Ag(PPh_3_)_2_}_2_(μ-κ,^2^κ^1^-CO_3_) was used with Cs_2_CO_3_ (0.75 equiv) as the base, the conversion improved to 98 and 99%, respectively (entries 2 and 3), and the side-product was no longer detected. With 5 mol % of Ag_2_CO_3_, the greatest conversion is 73% at 3 h, and the reaction reaches near-completion by 23 h (94% conversion) (entry 4). Trace amounts of the homocoupling side-product were also detected. The amount of Ag used can be decreased to 2.5 mol % without an appreciable decrease in the conversion, but when 1 mol % of Ag_2_CO_3_ was used, the ^1^H NMR yield dropped to 80% (entries 4 and 8). The use of 10 mol % Ag(C_6_F_5_) with 10 mol % PPh_3_ and Cs_2_CO_3_ (0.75 equiv) resulted in almost identical conversion (entry 9) to the experiments with other Ag complexes. These reactions demonstrate that the conversion can be increased to well above 90% by combining sub-stoichiometric Cs_2_CO_3_ with catalytic quantities of any of Ag_2_CO_3_, [Ag(PPh_3_)_2_(κ^2^-HCO_3_)]_2_, [Ag(PPh_3_)_2_]_2_CO_3_, or Ag(C_6_F_5_), thus eliminating the need for large quantities of silver salts.

**Table 3:**
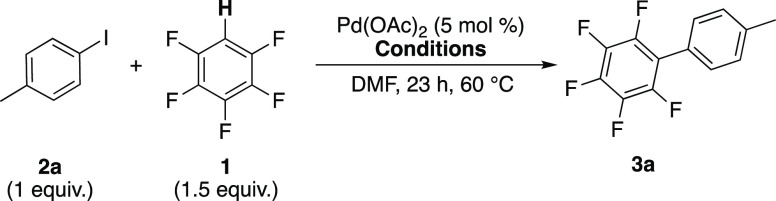
Influence of Catalytic Amounts of Ag Salts and Complexes on Direct Arylation

			^1^H NMR conversion^a^ (%)	
entry	PPh_3_ (mol %)	base Cs_2_CO_3_ (0.75 equiv) + Ag salt	3 h	23 h
1	10		15	69
2	5	[Ag(PPh_3_)_2_(κ^2^-HCO_3_)]_2_ (5 mol %)	51	98
3	5	[Ag(PPh_3_)_2_]_2_CO_3_ (5 mol %)	62	99
4	10	Ag_2_CO_3_ (5 mol %)	73	94
5	5	[Ag(PPh_3_)_2_(κ^2^-HCO_3_)]_2 **3**_ (2.5 mol %)	54	93
6	5	[Ag(PPh_3_)_2_]_2_CO_3_·2H_2_O (2.5 mol %)	59	96
7	10	Ag_2_CO_3_ (2.5 mol %)	57	89
8	10	Ag_2_CO_3_ (1 mol %)	44	80
9	10	Ag(C_6_F_5_) (10 mol %)	70	96

a The conversion was calculated by integrating the C*H*_3_ resonance of **2a** (δ 2.30) with respect to 1,3,5-trimethoxybenzene (δ 3.78) in the ^1^H NMR spectrum of the mixture with D1 = 1 s.

### Pd Speciation: Studies by HR-MAS and Conventional NMR

#### Identification of Catalytic Resting States

**Figure 3:**
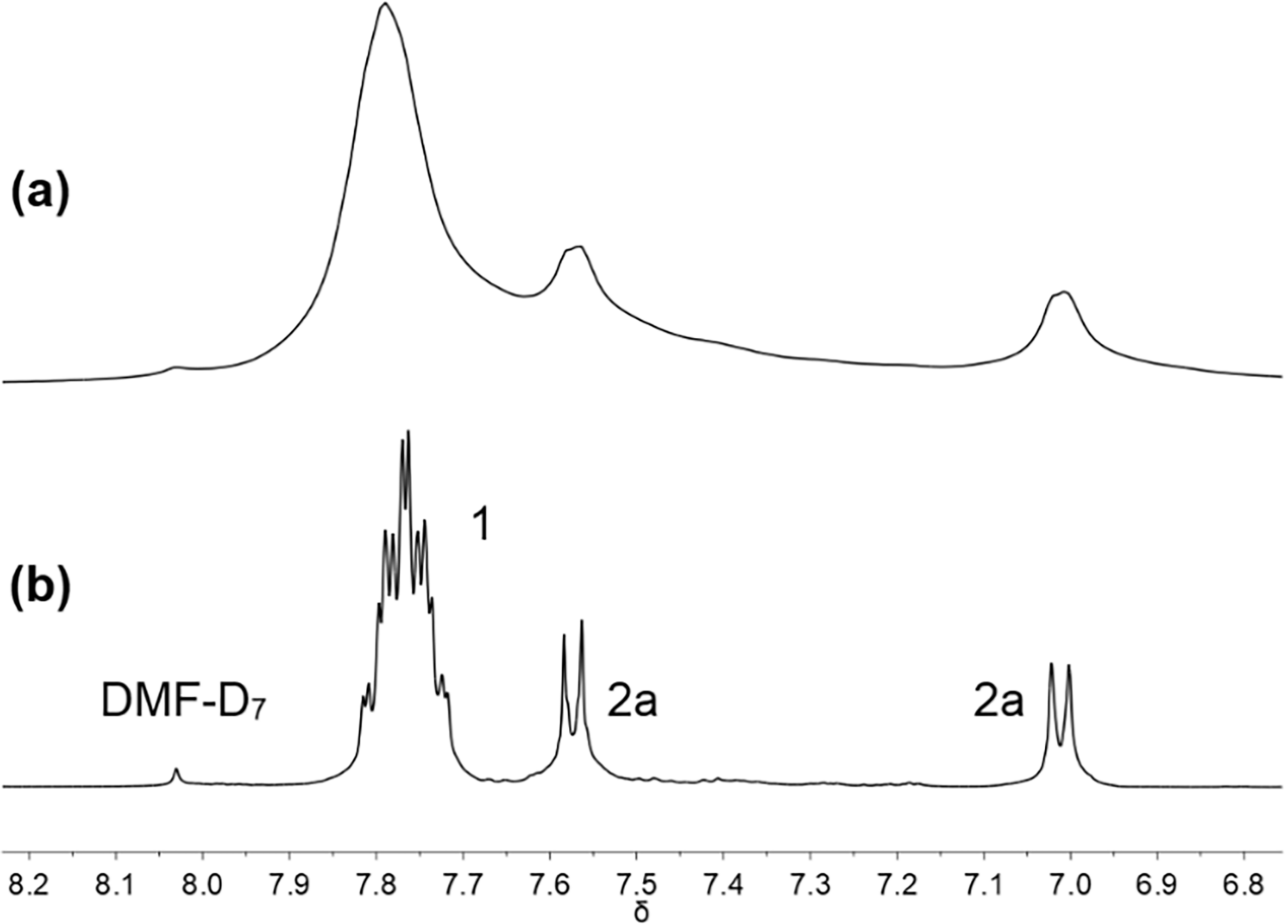
^1^H NMR spectra of a mixture of **1**, **2a**, and silver carbonate in DMF-*d*_7_ at 13 °C (a) without spinning and (b) spinning at 3 kHz.


The direct arylation of 4-iodobenzene **2b** with pentafluorobenzene **1** was selected as the model reaction system to be studied by HR-MAS NMR spectroscopy due to the ease of synthesis of phenyl-Pd species compared to their 4-tolyl analogues (Reaction R5). The line-broadening observed by standard NMR spectroscopy for the inhomogeneous reaction mixture was successfully resolved by the application of HR-MAS NMR spectroscopic analysis as shown in a test sample of **1**, **2a**, and Ag_2_CO_3_ (Figure 3).

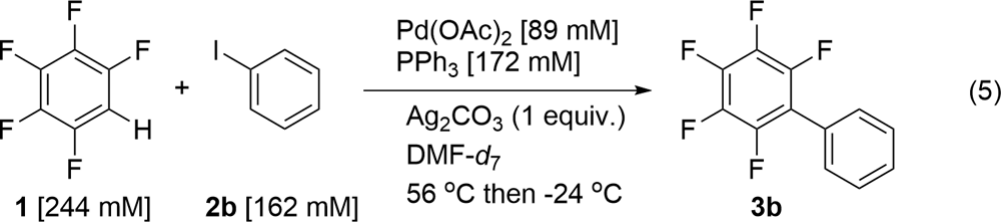
R5

**Scheme 3:**
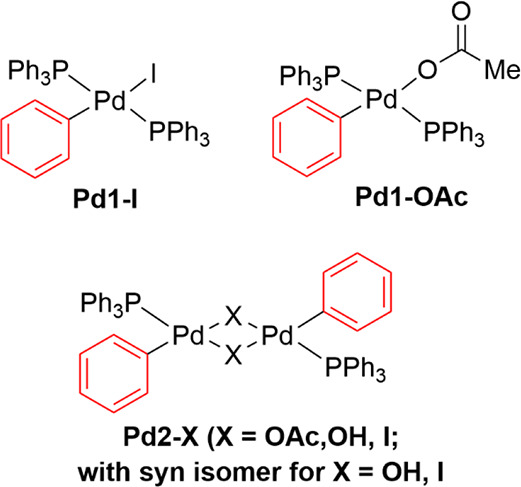
Potential Pd(II)-Resting States

The catalyst loading was increased from catalytic (i.e., 5 mol %) to sub-stoichiometric (i.e., 0.5 equiv) to observe possible species involved in the catalytic cycle. After heating at 56 °C in the rotor, broad phenyl signals were observed that disappeared on completion of the reaction. The reaction was repeated, but this time, the probe was cooled after 20% conversion to products. The broad signals observed at δ 6.83 and 6.69 during the reaction at 56 °C in the ^1^H NMR spectrum were resolved into two triplets at δ 6.91 and 6.75 at −24 °C. The carboxylic acid proton of the AcOH at δ 12.63 was also observed upon cooling. 2D ^1^H–^1^H COSY spectra revealed the presence of two species in the aromatic region (Figure S12). The triplet signal at δ 6.91 (*J* = 7.1 Hz) was correlated with the other triplet at δ 6.75 (*J* = 7.1 Hz) and to a signal at δ 7.29 overlapping with other aromatic signals. A second species was observed with correlation between weak signals at δ 6.29 and 6.53. Five well-known Pd complexes were synthesized for comparison and were heated to 56 °C for 90 min in DMF-*d*_7_ to simulate the catalytic conditions before measurement of NMR spectra at −24 °C (Scheme 3, Table S12).

**Figure 4:**
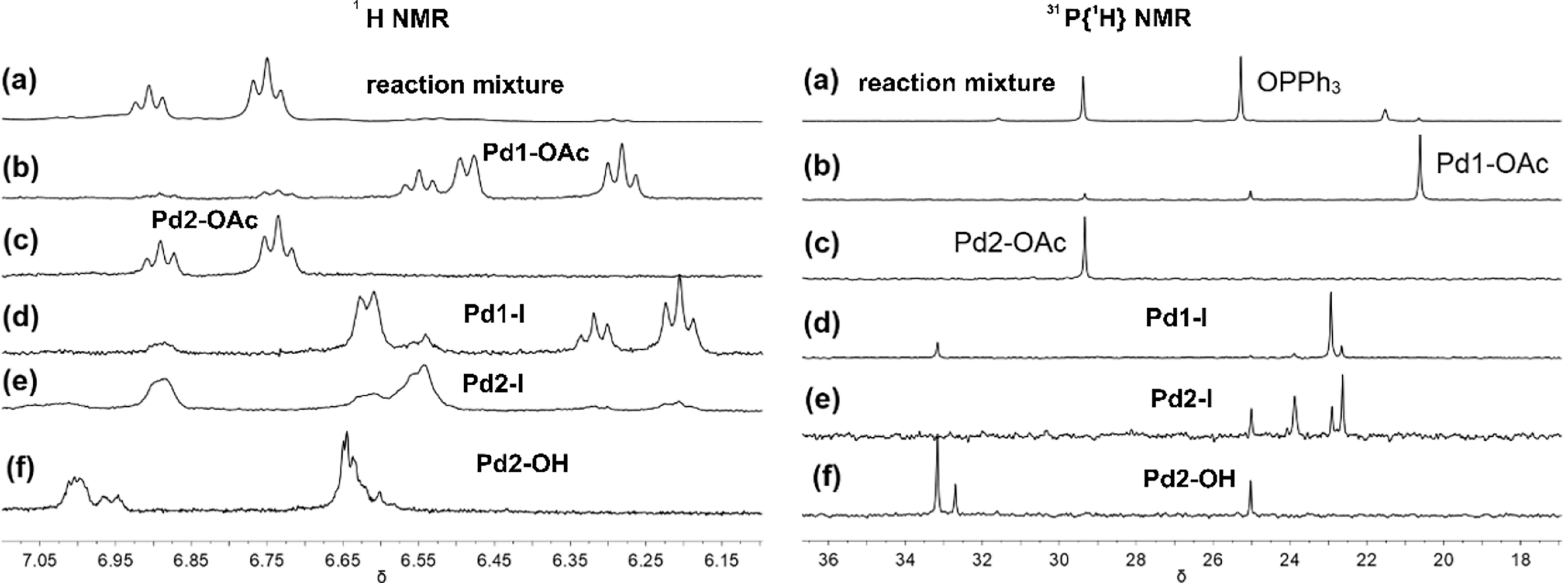
^1^H NMR spectra in the aromatic region (left) and ^31^P{^1^H} NMR spectra (right) of (a) the reaction shown in Reaction R5, (b) Pd(Ph)(κ^1^-OAc)(PPh_3_)_2_**Pd1-OAc**, (c) [Pd(Ph)(μ-OAc)(PPh_3_)]_2_**Pd2-OAc**, (d) Pd(Ph)(I)(PPh_3_)_2_**Pd1-I**, (e) [Pd(Ph)(μ-I)(PPh_3_)]_2_**Pd2-I**, and (f) [Pd(Ph)(μ-OH)(PPh_3_)]_2_**Pd2-OH**. Spectra collected after heating at 56 °C and measured at −24 °C in DMF-*d*_7_.

Comparison of the phenyl resonances and the ^31^P NMR signals of the Pd complexes with those of the intermediate observed during the reaction revealed that the reaction mixture contained **Pd2-OAc** with trace quantities of **Pd1-OAc** (Figure 4). Additionally, of the five Pd species analyzed, **Pd2-OAc** was the only complex retaining complete structural integrity after heating to 56 °C. Decomposition products such as OPPh_3_ were observed for other complexes; dinuclear **Pd2-OAc** and **Pd2-I** species were observed in the ^1^H and ^31^P NMR spectra of mononuclear **Pd1-OAc** and **Pd1-I** complexes, respectively.

The well-resolved phenyl signals of **Pd2-OAc** in DMF-*d*_7_ at 56 °C were broadened when the dinuclear complex was used as the catalyst for the reaction of **1** with **2b** (Reaction R6). It is likely that **Pd2-OAc** is in equilibrium with **Pd1-OAc**. Furthermore, the use of **Pd2-OAc** as the catalyst for the direct arylation reaction of **2a** with **1** yielded a mixture of two biaryl products in 3.5:1 ratio (**3a**:**3b**). Thus, **Pd2-OAc** reacted with **1** to form pentafluorophenylbenzene **3b** and then catalytically turned over **2a** to form the 4-(pentafluorophenyl)toluene **3a**.


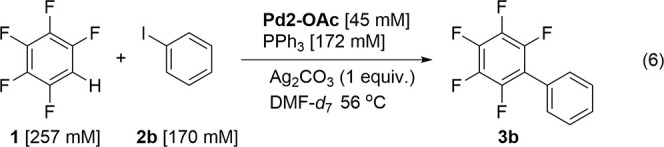
R6

Whereas the reaction catalyzed by 25 mol % **Pd2-OAc** achieved 12% product **3b** formation after 60 min, the addition of 25 mol % PPh_3_ improved the yield of **3b** to 25% after the same time. For the reaction with additional 75 mol % PPh_3_, 57% yield was achieved after 20 min. The addition of extra PPh_3_ resulted in increased formation of species with ^1^H and ^31^P{^1^H} NMR spectra very similar to those of **Pd1-OAc**.

**Figure 5:**
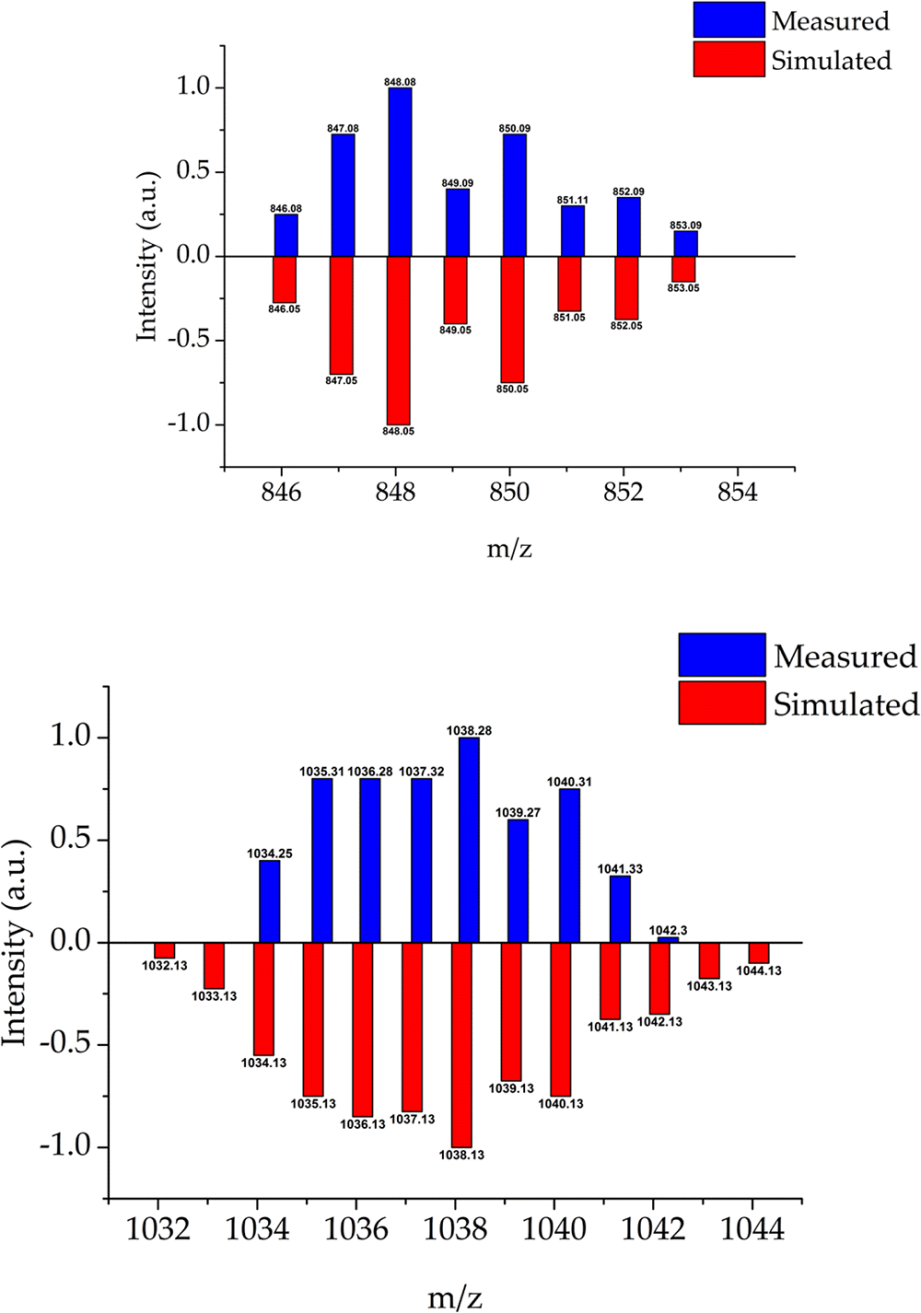
LIFDI-MS spectrum of (above) Pd(4-tolyl)(I)(PPh_3_)_2_ (PdC_43_H_37_IP_2_ requires 848.05 for ^106^Pd) and (below) [Pd(4-tolyl)(μ-OAc)(PPh_3_)]_2_ (Pd_2_C_54_H_50_O_4_P_2_ requires 1038.13 for ^106^Pd^108^Pd).

The involvement of **Pd2-OAc** and **Pd1-OAc** as catalytic resting states was further supported by *ex situ* LIFDI mass spectrometric studies.^87−89^ Reference spectra of Pd(tol)(κ^1^-OAc)(PPh_3_)_2_ and [Pd(tol)(μ-OAc)(PPh_3_)]_2_, the tolyl analogues of **Pd2-OAc** and **Pd-1-OAc**, are shown in Figure 5 (the LIFDI spectra of **Pd2-OAc** and **Pd-1-OAc** are in Figures S14 and S15). LIFDI spectra from the direct arylation reaction of **2a** with **1** revealed the [Pd(tol)(+μ-OAc)(PPh_3_)]_2_ and Pd(tol)(κ^1^-OAc)(PPh_3_)_2_ complexes at *m/z* = 518.05 and 1038.16, respectively, during the reaction (Table S13 and Figures S18–S21).

#### Stoichiometric Reaction of Palladium Species with Pentafluorobenzene

**Scheme 4:**
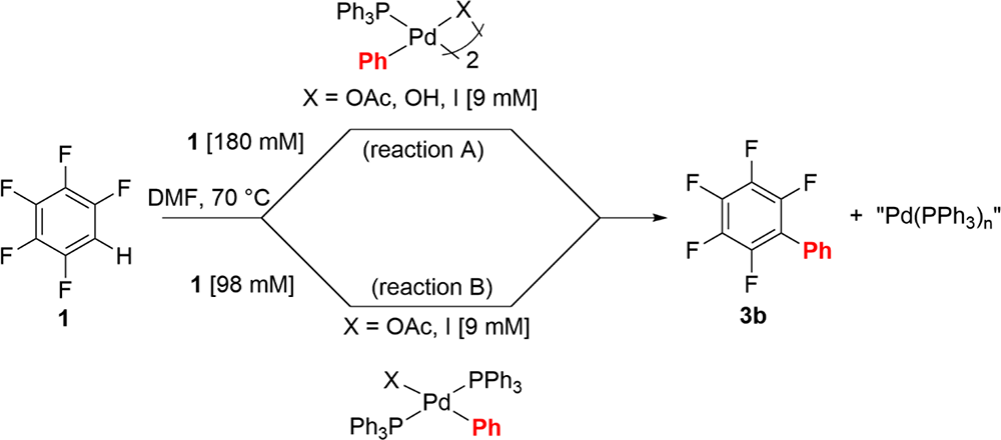
Stoichiometric Reaction of **1** in DMF [Reaction A: with Dinuclear Pd Species (X = OAc **Pd2-OAc**, OH **Pd2-OH**, and I **Pd2-I**); Reaction B: with Mononuclear Pd Species **Pd1-OAc** and Pd(Ph)(I)(PPh_3_)_2_**Pd1-I**]

The dinuclear species [Pd(Ph)(μ-X)(PPh_3_)]_2_ (where X = OAc **Pd2-OAc**, OH **Pd2-OH**, and I **Pd2-I**) were heated at 70 °C in the presence of 20-fold excess of pentafluorobenzene **1** in DMF (Scheme 4, reaction A, compare ref (32) and (33)). Formation of the product **3b** was observed for the reaction of **Pd2-OAc** and **Pd2-OH** in quantitative and 60% yields, respectively, but not for **Pd2-I** (Table 4, entries 1–3). However, partial formation of the product **3b** was observed for **Pd2-I** in the presence of Ag_2_CO_3_ or AgOAc (entries 4 and 5). The mononuclear species **Pd1-OAc** and **Pd1-I** were heated similarly (Scheme 4, reaction B). The **Pd1-OAc** complex formed the product **3b** in 45% yield (Table 4, entry 6). However, excellent conversion of the complex was achieved in the presence of Ag_2_CO_3_ (99%) and AgOAc (98%) (entries 7 and 8). As with the dinuclear iodide complex, the mononuclear Pd(Ph)(I)(PPh_3_)_2_**Pd1-I** complex was unreactive toward pentafluorobenzene **1** unless in the presence of Ag_2_CO_3_ (49%) and AgOAc (67%) (entries 9–11). No product was formed in the presence of AgBF_4_ (entry 12). In interpreting these results, it should be recalled that only **Pd2-OAc** is stable on heating to 70 °C.

**Table 4:** NMR Yields of **3b** from Stoichiometric Reactions of Pd Species and **1** in DMF^a^

entry	complex	base	NMR conversion (%)^b^
1	**Pd2-OAc**	none	99
2	**Pd2-OH**	none	60
3	**Pd2-I**	none	0
4	**Pd2-I**	3 equiv Ag_2_CO_3_	36
5	**Pd2-I**	7 equiv AgOAc	22
6	**Pd1-OAc**	none	45
7	**Pd1-OAc**	2 equiv Ag_2_CO_3_	99
8	**Pd1-OAc**	7 equiv AgOAc	98
9	**Pd1-I**	none	0
10	**Pd1-I**	2 equiv Ag_2_CO_3_	49
11	**Pd1-I**	4 equiv AgOAc	67
12	**Pd1-I**	7 equiv AgBF_4_	0

a Conditions as shown in Scheme 4 other than base.

b Based on integration of C_6_F_6_ (internal standard) and 3,5-fluorines of **3a** and **3b**.

#### Speciation in the Absence of Substrate

Recent studies have demonstrated the formation of Pd^I^ clusters with bridging phosphide ligands in the reactions of Pd(OAc)_2_ and PPh_3_ in THF.^53,90^ We were concerned to establish whether these species are formed under the conditions of our cross-coupling reactions. We therefore examined the nature of the Pd species formed in the absence of substrates by conventional ^31^P{^1^H} NMR spectroscopy, searching for characteristic phosphide resonances in the δ 200 region. Stoichiometric reactions of Pd(OAc)_2_ with PPh_3_ (2 equiv) in DMF for 20 min at 60 °C with and without Ag_2_CO_3_ yielded no evidence for phosphide species. There were traces of phosphide species after prolonged reaction (15 h) in the presence of Ag_2_CO_3_. Corresponding reactions in the presence of AgOAc yielded no evidence of phosphide species. The dominant product in all these reactions is OPPh_3_.

## Reaction Kinetics

### Monitoring the Reaction by IR Spectroscopy

**Figure 6:**
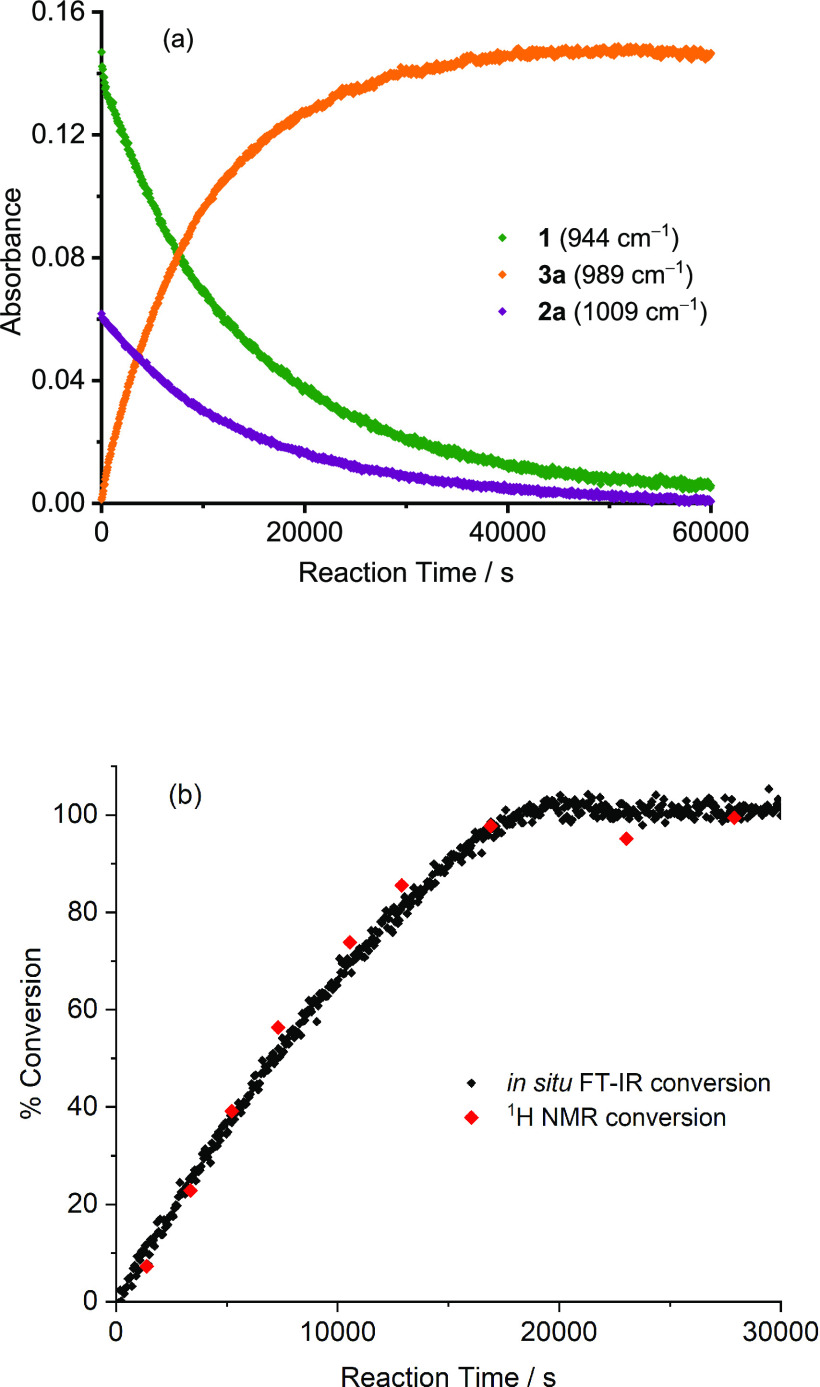
(a) Changes in the IR absorbance observed during the reaction shown in Reaction R7. Concentrations: pentafluorobenzene **1** (201 mM), 4-iodotoluene **2a** (200 mM), Pd(OAc)_2_ (10.1 mM), PPh_3_ (20.2 mM), with Ag_2_CO_3_ (0.75 equiv) in DMF at 56 ± 1 °C. (b) Progress of the reaction with 10-fold excess of **1** (*i.e.*, 0.18 M) generated by (black) *in situ* FTIR spectroscopy and (red) conversion determined by NMR spectroscopic analysis of sampled aliquots.


The course of the catalytic cross-coupling reaction could be monitored by *in situ* IR spectroscopy. Distinctive absorption bands were identified for pentafluorobenzene, iodotoluene, and their cross-coupling product in a region where the DMF solvent does not absorb significantly (Reaction R7). The *in situ* FTIR spectroscopic measurements were recorded by the ReactIR with a silicon probe dipping into a stirred flask with a thermocouple measuring the temperature in the solution. An example of the reaction profiles is shown in Figure 6a for a 1:1 substrate ratio and 5 mol % Pd(OAc)_2_. To validate the method, the conversion of 4-iodotoluene **2a** to biaryl product **3a** was determined by integrating the methyl peaks observed by ^1^H NMR analysis of aliquots sampled at regular intervals in a reaction with a 10:1 ratio of **1**:**2a** (Figure 6b). The kinetic profiles observed by FTIR and NMR were in good agreement. The reaction exhibited no induction period and reached 50% completion in ca. 6600 s and 90% completion after 15,000 s at 56 ± 1 °C under the conditions illustrated, making it clear that the temperature could be lowered and the reaction time reduced compared to the standard of Reaction R1.

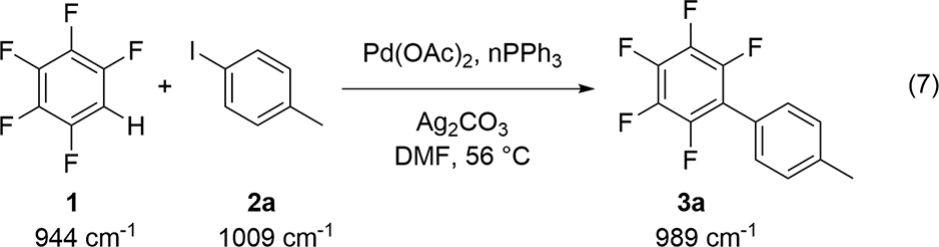
R7

### Determination of the Rate Law

**Figure 7:**
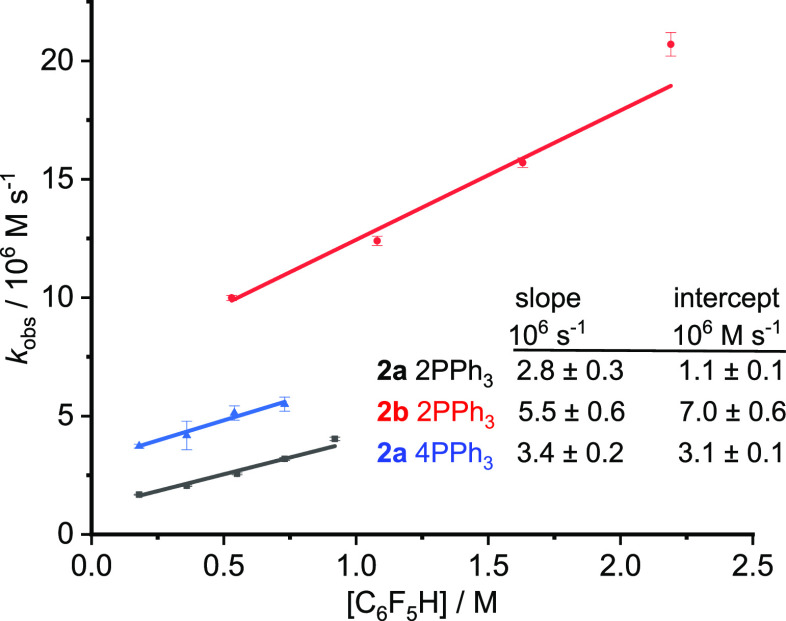
Dependence of pseudo-zero-order rate constant on [**1]**: black, reaction with **2a** with Pd/PPh_3_ 1:2; red, reaction with **2b** with Pd/PPh_3_ 1:2; blue, reaction with **2a** with Pd/PPh_3_ 1:4. Higher concentrations were used for **2b** than for **2a** because of lower IR absorption coefficients. [Catalyst] 5 mol % wrt **2a**/**2b**: 0.92 and 2.8 mM for reaction with **2a** and **2b**, respectively. [**2a**] 18 mM, [**2b**] 55 mM. Ag_2_CO_3_ 0.75 equiv, temp 56 ± 1 °C.

Detailed kinetic analysis was performed using this *in situ* IR approach by traditional kinetic methods and by variable time normalization analysis (VTNA) methods. Full details of the concentrations used together with the results from the VTNA methods are given in the Supporting Information (Figures S24–S26). Here we report the results from traditional isolation methods. Initial experiments were performed with a 10-fold excess of either **1** or **2a** (initial concentrations 0.018 and 0.18 M). With excess **1**, the decay of **2a** was linear to 50% conversion (*R*^2^ = 0.997); with excess **2a**, the decay of **1** was exponential with a linear fit to ln [**1**] to 50% conversion (*R*^2^ = 0.998). These results are consistent with a reaction that is zero order in **2a** and first order in **1**. A more detailed analysis of the dependence of pseudo-zero-order rate constant on [**1**] was carried out with [**1**]/[**2a**] ranging from 10 to 50, revealing a two-term rate law with slope (2.83 ± 0.26) × 10^–6^ s^–1^ and intercept (1.12 ± 0.10) × 10^–6^ mol dm^–3^ s^–1^ (Figure 7).

**Figure 8:**
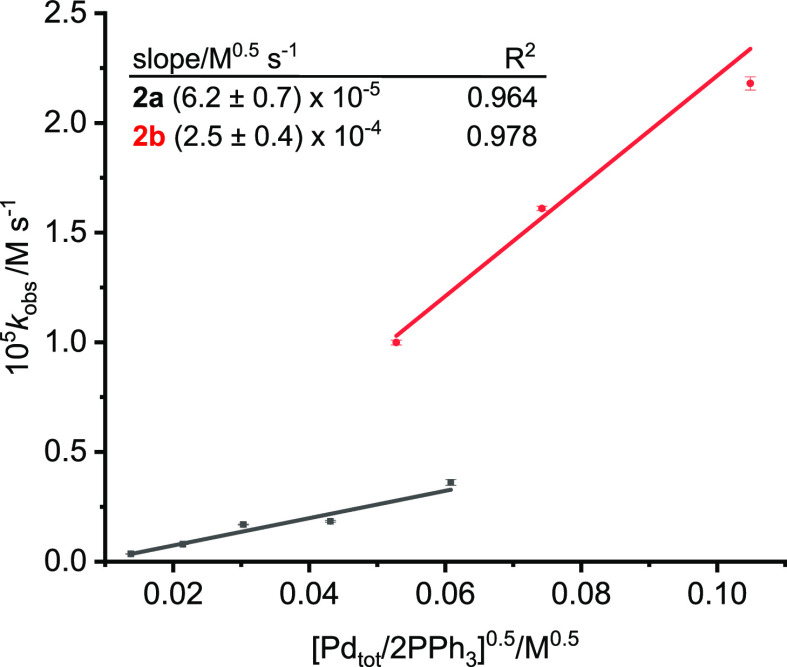
Plot of *k*_obs_ against [Pd_tot_/2PPh_3_]^1/2^ for the direct arylation reaction of 4-iodotoluene **2a** (black) and iodobenzene **2b** (red) at 56 ± 1 °C recorded under pseudo-zeroth-order conditions with 10-fold excess of **1**. Higher concentrations were used for **2b** than for **2a** because of lower IR absorption coefficients. [**1**] 180 mM for reaction with [**2a**] 18 mM, [**1**] 560 mM for reaction with [**2b**] 55 mM; Ag_2_CO_3_ 0.75 equiv, temp 56 ± 1 °C.

The rate dependence on [Pd_tot_/2PPh_3_] at 56 ± 1 °C was measured between 0.19 and 3.7 mM catalyst concentration under pseudo-zeroth-order reaction conditions with a 10-fold excess of pentafluorobenzene **1** (0.18 M) to 4-iodotoluene **2a** (0.018 M) (Table S14). Because the Pd(OAc)_2_/PPh_3_ ratio was maintained at 1:2 for each kinetic measurements, we refer to the concentration [Pd_tot_/2PPh_3_]. The plot of *k*_obs_ vs [Pd_tot_/2PPh_3_]^0.5^ gave better correlation coefficients (Figure 8) than the plot of *k*_obs_ vs [Pd_tot_/2PPh_3_]^0.75^ whose correlation coefficients, in turn, were much better than those for the plot of *k*_obs_ vs [Pd_tot_/2PPh_3_]. The kinetics was measured similarly for iodobenzene **2b** as substrate. The concentration of the limiting reagent **2b** was increased to 55 mM (from 18 mM used for **2a**) to allow for the lower peak intensities observed for **2b** (at 1016 cm^–1^) and the product **3b** (at 989 cm^–1^). The kinetics follows a similar pattern to that for **2a** (Figures 7 and 8).

Thus, the overall rate law is given by eq 2:
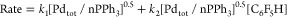
2

The values of *k*_1_ and *k*_2_ are estimated by dividing the slopes of Figure 7 by [Pd_tot_/2PPh_3_]^0.5^ (Table 5). The value of *k*_1_ is about 3 times larger for **2b** than for **2a**, but the values of *k*_2_ are very similar.

**Table 5:** Overall Rate Constants for Reaction of **1** with **2a** and **2b**^a^

entry	*k*_1_/10^–4^ M^1/2^ s^–1^	*k*_2_/10^–4^ M^–1/2^ s^–1^
**2a Pd 2PPh_3_**	0.336 ± 0.045	0.999 ± 0.075
**2b Pd 2PPh_3_**	1.14 ± 0.21	1.21 ± 0.14
**2a Pd 4PPh_3_**	1.02 ± 0.03	1.29 ± 0.081

a 56 ± 1 °C, DMF, Ag_2_CO_3_ 0.75 equiv.

Triphenylphosphine can play several roles including acting as a reducing agent for Pd(OAc)_2_, ligand for silver, and ligand for palladium. Moreover, several species may form in each role. For these reasons, we investigated the effect of the Pd/PPh_3_ ratio on the reaction kinetics. At a constant 5 mol % Pd(OAc)_2_, the reaction kinetics was studied with [PPh_3_] ranging from 5 mol % (1:1 Pd/PPh_3_) to 40 mol % (1:8 Pd/PPh_3_). The rate increased linearly up to 1:4 Pd/PPh_3_ but decreased slightly at higher concentrations than 1:4 and then leveled off (Figure S31). The dependence of the rate on [C_6_F_5_H] (at 0.93 mM catalyst) exhibited a very similar slope to that for 1:2 Pd/PPh_3_ but a markedly larger intercept (Figure 7). The variation of the rate with [Pd_tot_/4PPh_3_] was also determined (Figure S32); whereas the slope of *k*_obs_ vs [Pd_tot_/2PPh_3_]^0.5^ was (6.2 ± 0.7) × 10^–5^ M^0.5^ s^–1^, that of [Pd_tot_/2PPh_3_]^0.5^ was (15.6 ± 2.0) × 10^–5^ M^0.5^ s^–1^, an increase of a factor of 2.5. The overall rate constants are given in Table 5; the most marked change is the increase in the *k*_1_ term by a factor of 3. This change can be understood if the oxidative addition of aryl iodide precedes the rate determining step. Thus, the rate is determined by the reactivity of Pd(PPh_3_)_n_(Ar)I or related species (see below).

The reaction kinetics was also determined for a range of 4-substituted iodoarenes in place of iodotoluene. Additionally, the conversions were determined in competition between 4-iodotoluene and alternative 4-substituted iodoarenes. The effects of substitution were very minor (Table S22).

### Alternative Catalysts

The rate of coupling of pentafluorobenzene **1** with 4-iodotoluene **2a** using Ag_2_CO_3_ (0.75 equiv) and Pd(OAc)_2_/2PPh_3_ catalyst was compared to that with Pd(PPh_3_)_4_ at 50 ± 1 °C. The rates were (3.05 ± 0.03) × 10^–5^ and (9.12 ± 0.04) × 10^–5^ mol dm^–3^ s^–1^, respectively (Table S23, Figure S33). Considering that we know that the rate for Pd(OAc)_2_ is enhanced by a factor of 2.5 (at 56 °C) by increasing the PPh_3_/Pd ratio to 4, we conclude that the difference in performance of Pd(OAc)_2_/4PPh_3_ and Pd(PPh_3_)_4_ is very slight.

The kinetics of reactions in the presence of catalytic quantities of the isolated stable Pd^II^ species studied earlier by HR-MAS NMR was monitored by *in situ* FTIR spectroscopic analysis (Table 6). The mononuclear and the dinuclear complexes were added in 5 and 2.5 mol % loading, respectively, to give 5 mol % Pd-atom loading per reaction. It should be noted that some thermal decomposition at 56 ± 1 °C was observed by HR-MAS NMR study for every complex except for [Pd(Ph)(μ-OAc)(PPh_3_)]_2_. The kinetic profiles and the observed rate constants for the reactions catalyzed by the dinuclear Pd complex were compared with those for the catalyst mixture of 5 mol % Pd(OAc)_2_ and 10 mol % PPh_3_. Of the dinuclear Pd complexes tested, **Pd-2-OAc** and **Pd1-OAc** gave rates very close to Pd(OAc)_2_/2PPh_3_. **Pd1-I** was significantly faster, comparable to Pd(OAc)_2_/3PPh_3_. **Pd2-OH** and **Pd2-I** were ineffective when used alone but reached comparable rates with added AcOH.

**Table 6:** Observed Rate Constants for the Direct Arylation Reaction of **1** with **2a** Catalyzed by Isolated Pd Species

entry	catalyst (5 mol % Pd-atom)	*k*_obs_/10^–6^. mol dm^–3^ s^–1^
**1**	Pd(OAc)_2_ + 2PPh_3_	9.90 ± 0.04
**2**	**Pd2-OAc**	7.90 ± 0.05
**3**	**Pd2-OAc** + 2PPh_3_	7.56 ± 0.06
**4**	**Pd2-OH**	
**5**	**Pd2-I**	
**6**	**Pd2-OH** + 2AcOH	9.71 ± 0.10
**7**	**Pd2-I** + 2AcOH	8.32 ± 0.05
**8**	**Pd1-OAc**	8.52 ± 0.09
**9**	**Pd1-I**	14.83 ± 0.01
**10**	Pd(OAc)_2_ + 3PPh_3_	14.58 ± 0.01
**11**	**Pd1-I** + AcOH	7.34 ± 0.04
**12**	**Pd1-I** + 2AcOH	11.10 ± 0.14
**13**	**Pd1-I** + 4AcOH	14.00 ± 0.01

The role of Pd-NPs in the model reaction was considered by monitoring the reaction kinetics using preformed DMF-stabilized Pd-NP solution (0.9 mM). The reaction achieved 54% conversion of **1** after 40 h. In comparison, the reaction catalyzed by Pd(OAc)_2_ achieved quantitative conversion of substrate in 3 h (Figure S36). The result suggested that the two reactions are catalyzed by different active species and that the Pd-NPs were much less active.

### Kinetic Isotope Effect

**Figure 9:**
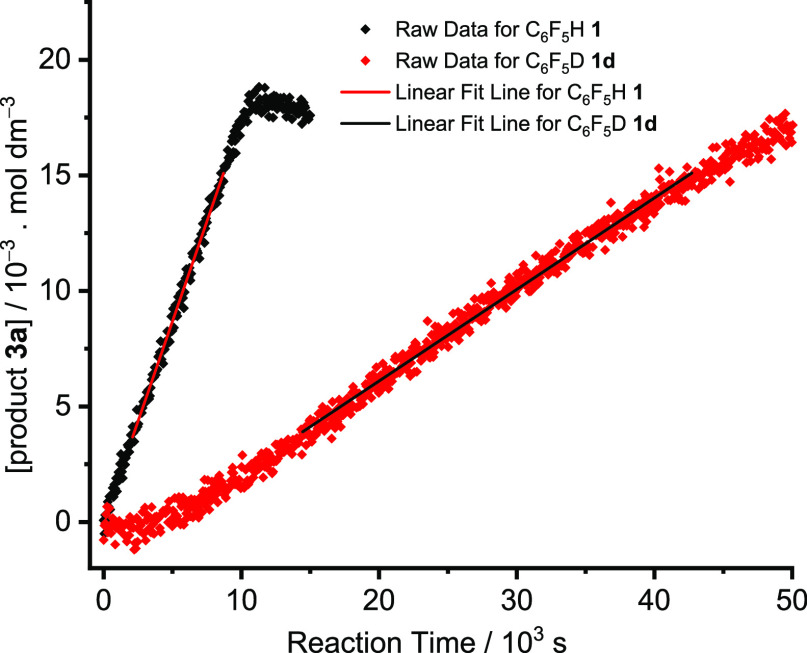
Change in the concentration of product **3a** over time for the reaction of **2a** with 10-fold excess of (black) C_6_F_5_H **1** and (red) C_6_F_5_D **1-d**. [Catalyst] 5 mol % wrt **2a**: 0.92 mM, [PPh_3_] 1.8 mM, [**1**] 180 mM, [**2a**] 18 mM. Ag_2_CO_3_ 0.75 equiv, temp 56 ± 1 °C.

The kinetic isotope effect was investigated by monitoring the reaction under pseudo-zeroth-order kinetics with 10-fold excess (*i.e.*, 0.18 M) of deuteropentafluorobenzene (C_6_F_5_D) **1-d** at 56 ± 1 °C with Pd(OAc)_2_/2PPh_3_. The IR spectrum of **1-d** was significantly different from that of C_6_F_5_H **1**. The characteristic peak of **2a** at 1009 cm^–1^ overlapped with the band of **1-d** at 1007 cm^–1^. However, it was possible to follow the reaction progress by observing the peak of the product **3a** at 989 cm^–1^. The *k*_obs_ values of separate reactions of **1** and **1-d** were obtained from the gradient between 20 and 80% conversion yielding *k*_H_/*k*_D_ 4.36 ± 0.06 at 56 ± 1 °C (Figure 9). These measurements were repeated with a higher concentration of **1**/**1-d** at both 56 and 40 °C, revealing a substantial reduction in KIE at higher concentrations of **1**/**1-d** and an increase in KIE on reduction of the temperature (Table 7). The KIE was also determined with Pd/PPh_3_ 1:4 as 3.86 ± 0.12 at 56 ± 1 °C with a 10-fold excess of **1**/**1-d**. A control reaction showed that no formation of **1** occurred according to ^1^H and ^19^F NMR spectroscopy under the standard reaction conditions when the direct arylation was carried out with **1-d**, showing that the C–H bond activation step is irreversible. Exchange does occur, however, with **1** in the presence of D_2_O and Ag_2_CO_3_ and PPh_3_ as described above.

**Table 7:** KIE for the Direct Arylation Reaction of **2a** by **1** or **1-d** at Different Reaction Temperatures and Different Concentrations of **1**/**1-d**^a^

*T*/°C	catalyst	[**1**] or [**1-d**]/M	isotope	*k*_obs_/10^–7^ M s^–1^	KIE^a^ (*k*_obs(H)_/*k*_obs(D)_)
40 ± 1	Pd(OAc)_2_/2PPh_3_	0.18	H	4.19 ± 0.02	5.47 ± 0.05
			D	0.765 ± 0.002	
		0.73	H	9.50 ± 0.12	2.97 ± 0.05
			D	3.20 ± 0.02	
56 ± 1	Pd(OAc)_2_/2PPh_3_	0.18	H	17.3 ± 0.2	4.36 ± 0.06
			D	3.96 ± 0.02	
		0.73	H	29.4 ± 0.3	2.30 ± 0.04
			D	12.8 ± 0.1	
56 ± 1	Pd(OAc)_2_/4PPh_3_	0.18	H	27.4 ± 0.4	3.86 ± 0.12
			D	7.09 ± 0.11	

a *k*_obs_ obtained under pseudo-zeroth-order rate law with [**2a**] = 18 mM.

The values of the KIE reported above are derived directly from *k*_obs_ and therefore represent a composite of the *k*_1_ and *k*_2_ terms. Because the *k*_1_ term is independent of [**1**], it is expected to have a KIE of 1.0. However, close inspection of the data shows that the *k*_1_ term is much less important for **1-d**. The *k*_2_ term is dominant at higher [**1**], suggesting that the true value of the KIE for this term is ∼2.30. The origin of the paradoxical behavior is probed further in the discussion.

### Temperature Dependence of Rates

**Figure 10:**
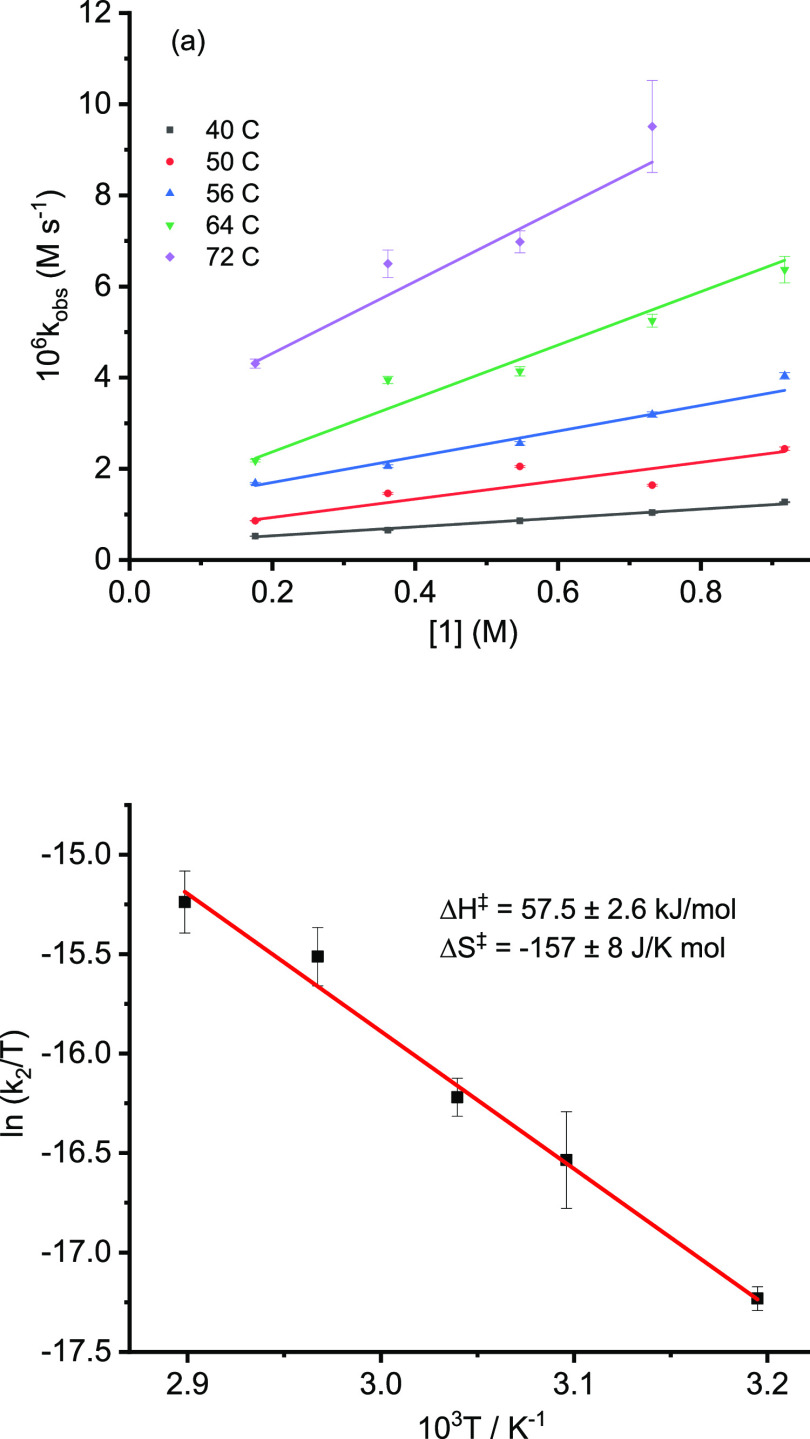
(a). Plot of *k*_obs_ against [**1**] for the catalytic reaction of **1** with **2a** catalyzed by Pd(OAc)_2_ with 2 equiv PPh_3_ at 40 ± 1, 50 ± 1, 56 ± 1, 64 ± 1, and 72 ± 1 °C with lines of best fit. (b) Eyring plot for values of *k*_2_ derived assuming the reaction is half order in [Pd_tot_/2PPh_3_].

The temperature dependence of the rate of the catalytic reaction between **1** and **2a** was studied at varying concentrations of **1** under the same conditions as in Figure 7 over the temperature range 323–345 K (Figure 10a). After conversion of the resulting slopes and intercepts to *k*_1_ and *k*_2_ assuming half-order in [Pd_tot_], Eyring plots yielded values of Δ*H*^‡^ and Δ*S*^‡^ (Figure 10b). The values of Δ*H*^‡^ for the *k*_1_ and *k*_2_ terms were 57.4 ± 4.8 and 57.5 ± 2.6 kJ/mol, and those for Δ*S*^‡^ were −166 ± 15 and −157 ± 8 J/K mol. These values are the same within error, suggesting that there is a link between them; but at present, we have not identified the cause.

## Discussion

The results in this paper show that direct arylation of 4-iodotoluene **2a** with pentafluorobenzene **1** is effective using readily available Pd^II^ and Pd^0^ precatalysts Pd(OAc)_2_/PPh_3_, Pd(PPh_3_)_2_Cl_2_, and Pd(PPh_3_)_4_. The most effective bases were found to be Ag_2_CO_3_ and [Me_4_N]OAc when used in conjunction with polar aprotic solvents such as DMF, DMAc, and NMP. The system with Pd(OAc)_2_/PPh_3_ and Ag_2_CO_3_ in DMF was studied in detail.

The first indications of the reactivity of Ag_2_CO_3_ toward **1** came from H/D exchange experiments. Investigation of the reactions of PPh_3_ and pentafluorobenzene with Ag_2_CO_3_ showed the formation of Ag(C_6_F_5_)(PPh_3_)_n_ species, but low-temperature ^31^P{^1^H} NMR revealed the presence of numerous species at equilibrium. Likewise, the previously described silver carbonate [Ag(PPh_3_)_2_]_2_CO_3_·2H_2_O proved highly labile and reactive toward **1** even at room temperature. [Ag(PPh_3_)_2_(κ^2^-HCO_3_)]_2_ was also reactive if Cs_2_CO_3_ was added. Related species could be obtained by reaction of Ag(C_6_F_5_) with PPh_3_. Considering the reactivity of the silver compounds toward **1**, we tested whether the standard 0.75 equiv Ag_2_CO_3_ used in the catalytic reaction of **1** with **2a** could be reduced to a catalytic amount. Indeed, catalytic quantities of any of Ag_2_CO_3_, [Ag(PPh_3_)_2_]_2_CO_3_·2H_2_O, or [Ag(PPh_3_)_2_(κ^2^-HCO_3_)]_2_ were effective if used in conjunction with Cs_2_CO_3_ as sub-stoichiometric base. These experiments indicate that the Ag_2_CO_3_/PPh_3_ system is competent to activate the C–H bond of **1** in a similar way to Ag_2_CO_3_/XPhos^45^ but the PPh_3_ system is complicated by the extreme lability of the Ag–PPh_3_ bonds. We therefore have to consider the possibility that C–H activation by Ag^I^ competes with C–H activation by Pd^II^.

Studies of the reactivity of **1** with iodobenzene **2b** in the presence of a variety of Pd complexes and Ag_2_CO_3_ by HR-MAS NMR led to the identification of [Pd(C_6_H_5_)(μ-OAc)(PPh_3_)]_2_**Pd2-OAc** and Pd(C_6_H_5_)(κ^1^-OAc)(PPh_3_)_2_**Pd1-OAc** under high Pd(OAc)_2_ concentrations. The stoichiometric reaction of the isolated Pd species **Pd2-OAc** with **1** at 70 °C yielded the expected coupling product **3b** quantitatively, whereas the reaction of **Pd1-OAc** with **1** achieved 45% yield without Ag_2_CO_3_ and 100% with Ag_2_CO_3_. These investigations lead to the postulate of **Pd1-OAc** and **Pd2-OAc** as likely resting states in the catalytic reaction. Other species such as **Pd1-I** and **Pd2-I** are also active but less effective than the acetate complexes. Evidently, multiple Pd species are present, and the speciation will vary with PPh_3_ concentration and temperature.

We now consider the interpretation of the kinetic results. The *k*_2_ term of the rate law (eq 2) shows that the rate depends on [C_6_F_5_H] and [Pd_tot_/2PPh_3_]^0.5^_,_ a similar rate expression to that described by Rosner et al.^91^ This expression is consistent with a catalytically active mononuclear Pd^0^ species that reacts rapidly with ArI to form Pd^II^ species present in monomeric and dimeric forms. The monomer is active in the catalytic cycle, but the dimeric species with which it is at equilibrium is inactive. The concentration of the monomer is given by *K*_eq_[dimer]^0.5^, leading to the half-order dependence. The monomer proceeds to react with C_6_F_5_H and base before reductive elimination of product ensues. The small value of Δ*H*^‡^ and the large negative Δ*S*^‡^ are consistent with a bimolecular reaction in the rate determining transition state.^92^ This catalytic cycle is similar to that shown in Scheme 1a with the addition of the monomer–dimer equilibrium. It is tempting to identify the monomer and dimer as **Pd1-OAc** and **Pd2-OAc** (or their tolyl equivalents) that were identified as resting states through the HR-MAS NMR experiments. The value of *k*_2_ is little changed when using a 4:1 ratio of [PPh_3_]/[Pd(OAc)_2_] in place of a 2:1 ratio, indicating that the Pd^II^ speciation is little affected by this change. The significant KIE (∼2.30 taken from the higher [**1**] at 56 °C) and substantial entropy of activation are consistent with this model. There are several indications that this model of the *k*_2_ reaction may be oversimplified and that multiple species contribute. First, the experiments with carbonate complexes of silver show that multiple complexes of Ag^I^ with PPh_3_ and carbonate are present and could be active as bases; second, the HR-MAS NMR experiments show the presence and activity of several Pd^II^ species. The variation of the KIE with reaction conditions is a likely consequence of the complex speciation.

The *k*_1_ term of the rate law shows dependence on [Pd_tot_/2PPh_3_]^0.5^ and is increased by a factor of ∼2.5 with a 4:1 ratio of PPh_3_ to Pd_tot_. The absence of a dependence on [C_6_F_5_H] is consistent with C–H activation of **1** by Ag^I^(PPh_3_)_n_ followed by transmetalation to any of **Pd1-OAc**, **Pd2-OAc**, **Pd1-I**, or **Pd2-I**. The dependence on [Pd_tot_/2PPh_3_]^0.5^ indicates that the monomer–dimer equilibrium remains important and that the rate-determining transition state is likely to be associated with the transmetalation and/or reductive elimination. Considering the negative value of Δ*S*^‡^ and small value of Δ*H*^‡^, transmetalation is more likely. Direct participation of the solvent could also be possible. The dependence on [PPh_3_] is consistent with the requirement for sufficient ligand to solubilize the Ag_2_CO_3_ and activate **1**; the concentrations of PPh_3_ are suboptimal at the lower ratio because PPh_3_ is consumed both by reduction of Pd^II^ to Pd^0^ with formation of OPPh_3_ and by coordination to Ag^I^. To understand the variation of KIE with conditions, we recall that the KIE for the stoichiometric reaction of [Ag(Xphos)]_2_(μ-κ*,*^2^κ^2^-CO_3_) with pentafluorobenzene is 3.7 ± 0.3, a value that we may assume is close to that for PPh_3_ analogues. This value would mean that the *k*_1_ term is slowed more than the *k*_2_ term by deuteration, and we postulate that the Ag^I^ route to C–D activation is uncompetitive with the Pd^II^ route. Unfortunately, we cannot monitor the rate of C–H/C–D activation by Ag(PPh_3_)_n_ species because of their lability and the consequent multiple speciation. The similarity of the rate constants for different catalysts Pd(OAc)_2_/nPPh_3_, Pd(PPh_3_)_4_, **Pd-2OAc**, **Pd1-OAc**, and **Pd1-I** points to common mechanisms. We anticipate that the catalytic mechanism involving bond activation by Ag^I^ is similar to that shown in Scheme 1b with the proviso of multiple speciation of both silver and palladium complexes that vary with conditions.

## Conclusions

The direct arylation of iodoarenes by pentafluorobenzene **1** is a prototype reaction that is catalyzed by palladium complexes in the presence of triphenylphosphine and silver carbonate. The reaction is typically performed with 0.75 equiv Ag_2_CO_3_, but the use of Ag_2_CO_3_ may be reduced to catalytic quantities in the presence of Cs_2_CO_3_. The simplicity of the components does not mean, however, that the reaction mechanism is straightforward. Our study has demonstrated a Pandora’s flask of multiple species that can only be interpreted through previous research on the reactivity of analogous systems using phosphines such as XPhos. Both *in situ* IR spectroscopy and high-resolution magic angle spinning NMR spectroscopy proved useful in our analysis, in addition to solution NMR methods. We have shown that the reaction of Ag_2_CO_3_ with PPh_3_ and **1** leads to C–H bond activation with multiple Ag complexes present in solution. The same applies when starting with isolated complexes such as [Ag(PPh_3_)_2_]_2_CO_3_·2H_2_O. The potential for C–H activation by Ag^I^ aligns with our own experiments using XPhos in place of PPh_3_ and the reactions investigated notably by Larrosa, Sanford, and Hartwig with their co-workers.^34−43^ The problem of multiple speciation also applies to the palladium complexes, although it is not so acute. Our HR-MAS experiments point to [Pd(Ar)(μ-OAc)(PPh_3_)]_2_ and Pd(Ar)(κ^1^-OAc)(PPh_3_)_2_ (Ar = Ph, 4-tolyl) as likely resting states that are at equilibrium, with the dimer as the major species. The reaction kinetics studied by *in situ* IR spectroscopy demonstrates that synthetic experiments may be performed with a 1:1 ratio of [ArI]/[C_6_F_5_H] under milder conditions (56 °C, 5 h) than are typically used (70 °C, 24 h). Rates are improved with Pd(OAc)_2_ + 4PPh_3_ or Pd(PPh_3_)_4_ compared with the standard Pd(OAc)_2_ + 2PPh_3_ in keeping with the multiple roles of PPh_3_ for coordination of both Ag and Pd, as well as reduction of Pd(OAc)_2_. Higher [PPh_3_] also favors monomeric Pd complexes over dimeric complexes. Similar rate constants are found with [Pd(Ph)(μ-OAc)(PPh_3_)]_2_ and Pd(Ph)(κ^1^-OAc)(PPh_3_)_2_ as catalysts. A two-term rate law is found with both terms dependent on [Pd_tot_/2PPh_3_]^0.5^, consistent with an off-cycle Pd dimer dissociating to form an on-cycle Pd monomer. The *k*_1_ term is independent of [**1**], whereas the *k*_2_ term is first order in [**1**]. Neither term shows dependence on [ArI]. We interpret the *k*_1_ term as arising from C–H bond activation by Ag^I^, whereas the *k*_2_ term involves C–H activation by Pd^II^. Both processes require base-assistance and may proceed by AMLA/CMD mechanisms. The multiple speciation of both Ag^I^(PPh_3_) and Pd^II^(PPh_3_) complexes leads to a situation where it is no longer appropriate to define a mechanism in full. In particular, the implications of the extraordinary lability of Ag^I^(PPh_3_) species have not been recognized previously. Thus, a reaction with apparently well-behaved kinetics disguises extraordinary complexity. It may be possible to suppress C–H bond activation by one of Pd^II^ or Ag^I^ by adding appropriate inhibitors.^49^

## Supporting Information Available

General information on chemicals and instruments, general procedures, synthetic procedures and compound data, studies on conversion, speciation of silver complexes, analysis of catalytic intermediates by HR-MAS NMR spectroscopy, and reaction kinetics by *in situ* IR spectroscopy (PDF)

## References

[ref1] Constable D. J. C.; Dunn P. J.; Hayler J. D.; Humphrey G. R.; Leazer J. L. Jr.; Linderman R. J.; Lorenz K.; Manley J.; Pearlman B. A.; Wells A.; Zaks A.; Zhang T. Y. Key green chemistry research areas - a perspective from pharmaceutical manufacturers. Green Chem. 2007, 9, 411–420. 10.1039/B703488C.

[ref2] Alberico D.; Scott M. E.; Lautens M. Aryl-aryl bond formation by transition-metal-catalyzed direct arylation. Chem. Rev. 2007, 107, 174–238. 10.1021/cr0509760. 17212475

[ref3] Ishiyama T.; Takagi J.; Ishida K.; Miyaura N.; Anastasi N. R.; Hartwig J. F. Mild iridium-catalyzed borylation of arenes. High turnover numbers, room temperature reactions, and isolation of a potential intermediate. J. Am. Chem. Soc. 2002, 124, 390–391. 10.1021/ja0173019. 11792205

[ref4] Ackermann L. Carboxylate-assisted transition-metal-catalyzed C-H bond functionalizations: mechanism and scope. Chem. Rev. 2011, 111, 1315–1345. 10.1021/cr100412j. 21391562

[ref5] Park C. H.; Ryabova V.; Seregin I. V.; Sromek A. W.; Gevorgyan V. Palladium-catalyzed arylation and heteroarylation of indolizines. Org. Lett. 2004, 6, 1159–1162. 10.1021/ol049866q. 15040747 PMC3708550

[ref6] Lane B. S.; Brown M. A.; Sames D. Direct palladium-catalyzed C-2 and C-3 arylation of indoles: A mechanistic rationale for regioselectivity. J. Am. Chem. Soc. 2005, 127, 8050–8057. 10.1021/ja043273t. 15926829

[ref7] Lafrance M.; Fagnou K. Palladium-catalyzed benzene arylation: Incorporation of catalytic pivalic acid as a proton shuttle and a key element in catalyst design. J. Am. Chem. Soc. 2006, 128, 16496–16497. 10.1021/ja067144j. 17177387

[ref8] Campeau L. C.; Rousseaux S.; Fagnou K. A solution to the 2-pyridyl organometallic cross-coupling problem: Regioselective catalytic direct arylation of pyridine N-oxides. J. Am. Chem. Soc. 2005, 127, 18020–18021. 10.1021/ja056800x. 16366550

[ref9] Lafrance M.; Rowley C. N.; Woo T. K.; Fagnou K. Catalytic intermolecular direct arylation of perfluorobenzenes. J. Am. Chem. Soc. 2006, 128, 8754–8756. 10.1021/ja062509l. 16819868

[ref10] He M.; Soule J.-F.; Doucet H. Synthesis of (Poly)fluorobiphenyls through Metal-catalyzed C-H Bond Activation/Arylation of (Poly)fluorobenzene Derivatives. ChemCatChem 2014, 6, 1824–1859. 10.1002/cctc.201402020.

[ref11] Sato R.; Yasuda T.; Hiroto T.; Kanbara T.; Kuwabara J. Facile Synthesis of Bis-pentafluoroarylated Anthracene Derivatives for N-type Organic-Field-Effect Transistor Applications. Chem. – Eur. J. 2023, 29, e202203816 10.1002/chem.202203816. 36655930

[ref12] Boyaala R.; Peng M.; Tai W.-S.; Touzani R.; Roisnel T.; Dorcet V.; Chi Y.; Guerchais V.; Doucet H.; Soule J.-F. Exploiting the Reactivity of Fluorinated 2-Arylpyridines in Pd-Catalyzed C-H Bond Arylation for the Preparation of Bright Emitting Iridium(III) Complexes. Inorg. Chem. 2020, 59, 13898–13911. 10.1021/acs.inorgchem.0c01528. 32945677

[ref13] Yuen O. Y.; Leung M. P.; So C. M.; Sun R. W.-Y.; Kwong F. Y. Palladium-Catalyzed Direct Arylation of Polyfluoroarenes for Accessing Tetra-ortho-Substituted Biaryls: Buchwald-type Ligand Having Complementary -PPh2 Moiety Exhibits Better Efficiency. J. Org. Chem. 2018, 83, 9008–9017. 10.1021/acs.joc.8b01176. 29882668

[ref14] Shi X. Z.; Mao S. X.; Soule J.-F.; Doucet H. Reactivity of 1,2,3-and 1,2,4-Trifluorobenzenes in Palladium-Catalyzed Direct Arylation. J. Org. Chem. 2018, 83, 4015–4023. 10.1021/acs.joc.8b00412. 29537843

[ref15] Wang J.; Meng G.; Xie K.; Li L.; Sun H.; Huang Z. Mild and Efficient Ni-Catalyzed Biaryl Synthesis with Polyfluoroaryl Magnesium Species: Verification of the Arrest State, Uncovering the Hidden Competitive Second Transmetalation and Ligand-Accelerated Highly Selective Monoarylation. ACS Catal. 2017, 7, 7421–7430. 10.1021/acscatal.7b02618.

[ref16] Yang G.; Jiang X.; Liu Y.; Li N.; Yin G.; Yu C. Palladium-Catalyzed Direct Benzylation of Polyfluoroarenes with Benzyl Carbonates through Selective C-H Functionalization. Asian J. Org. Chem. 2016, 5, 882–885. 10.1002/ajoc.201600204.

[ref17] Maalik A.; Sharif M.; Abbas N.; Spannenberg A.; Villinger A.; Langer P. Synthesis and photophysical properties of tetra and pentaarylated fluorobenzenes. Tetrahedron 2016, 72, 1076–1082. 10.1016/j.tet.2016.01.009.

[ref18] Otsuka S.; Yorimitsu H.; Osuka A. Palladium-Catalyzed Zinc-Amide-Mediated C-H Arylation of Fluoroarenes and Heteroarenes with Aryl Sulfides. Chem. – Eur. J. 2015, 21, 14703–14707. 10.1002/chem.201502101. 26235212

[ref19] Fu Z.; Xiong Q.; Zhang W.; Li Z.; Cai H. Pd-catalyzed direct arylation of electron-deficient polyfluoroarenes with aryliodine(III) diacetates. Tetrahedron Lett. 2015, 56, 123–126. 10.1016/j.tetlet.2014.11.033.

[ref20] Ma X.; Liu Y.; Liu P.; Xie J.; Dai B.; Liu Z. Palladium-catalyzed direct arylation of polyfluoroarene and facile synthesis of liquid crystal compounds. Appl. Organomet. Chem. 2014, 28, 180–185. 10.1002/aoc.3106.

[ref21] Palani V.; Perea M. A.; Sarpong R. Site-Selective Cross-Coupling of Polyhalogenated Arenes and Heteroarenes with Identical Halogen Groups. Chem. Rev. 2022, 122, 10126–10169. 10.1021/acs.chemrev.1c00513. 34402611 PMC9616176

[ref22] Guihaumé J.; Clot E.; Eisenstein O.; Perutz R. N. Importance of palladium-carbon bond energies in direct arylation of polyfluorinated benzenes. Dalton Trans. 2010, 39, 10510–10519. 10.1039/c0dt00296h. 20931129

[ref23] Gorelsky S. I. Origins of regioselectivity of the palladium-catalyzed (aromatic)C-H bond metalation-deprotonation. Coord. Chem. Rev. 2013, 257, 153–164. 10.1016/j.ccr.2012.06.016.

[ref24] Clot E.; Mégret C.; Eisenstein O.; Perutz R. N. Exceptional Sensitivity of Metal-Aryl Bond Energies to ortho-Fluorine Substituents: Influence of the Metal, the Coordination Sphere, and the Spectator Ligands on M-C/H-C Bond Energy Correlations. J. Am. Chem. Soc. 2009, 131, 7817–7827. 10.1021/ja901640m. 19453181

[ref25] Hammarback L. A.; Bishop A. L.; Jordan C.; Athavan G.; Eastwood J. B.; Burden T. J.; Bray J. T. W.; Clarke F.; Robinson A.; Krieger J. P.; Whitwood A.; Clark I. P.; Towrie M.; Lynam J. M.; Fairlamb I. J. S. Manganese-Mediated C-H Bond Activation of Fluorinated Aromatics and the ortho-Fluorine Effect: Kinetic Analysis by In Situ Infrared Spectroscopic Analysis and Time-Resolved Methods. ACS Catal. 2022, 12, 1532–1544. 10.1021/acscatal.1c05477.

[ref26] Pabst T. P.; Chirik P. J. A Tutorial on Selectivity Determination in C(sp^2^)-H Oxidative Addition of Arenes by Transition Metal Complexes. Organometallics 2021, 40, 813–831. 10.1021/acs.organomet.1c00030. 33867622 PMC8045024

[ref27] Lee B.; Pabst T. P.; Chirik P. J. Effect of Pincer Methylation on the Selectivity and Activity in (PNP)Cobalt-Catalyzed C(sp^2^)-H Borylation. Organometallics 2021, 40, 3766–3774. 10.1021/acs.organomet.1c00499. 34898806 PMC8656436

[ref28] Lapointe D.; Fagnou K. Overview of the Mechanistic Work on the Concerted Metallation-Deprotonation Pathway. Chem. Lett. 2010, 39, 1119–1126.

[ref29] Ackermann L.; Vicente R.; Althammer A. Assisted ruthenium-catalyzed C-H bond activation: Carboxylic acids as cocatalysts for generally applicable direct arylations in apolar solvents. Org. Lett. 2008, 10, 2299–2302. 10.1021/ol800773x. 18439021

[ref30] Boutadla Y.; Davies D. L.; Macgregor S. A.; Poblador-Bahamonde A. I. Mechanisms of C-H bond activation: rich synergy between computation and experiment. Dalton Trans. 2009, 5820–5831. 10.1039/b904967c. 19623381

[ref31] Davies D. L.; Macgregor S. A.; McMullin C. L. Computational Studies of Carboxylate-Assisted C-H Activation and Functionalization at Group 8-10 Transition Metal Centers. Chem. Rev. 2017, 117, 8649–8709. 10.1021/acs.chemrev.6b00839. 28530807

[ref32] Wakioka M.; Nakamura Y.; Wang Q.; Ozawa F. Direct Arylation of 2-Methylthiophene with Isolated [PdAr(μ-O_2_CR)(PPh_3_)]_n_ Complexes: Kinetics and Mechanism. Organometallics 2012, 31, 4810–4816. 10.1021/om300367k.

[ref33] Wakioka M.; Nakamura Y.; Hihara Y.; Ozawa F.; Sakaki S. Factors Controlling the Reactivity of Heteroarenes in Direct Arylation with Arylpalladium Acetate Complexes. Organometallics 2013, 32, 4423–4430. 10.1021/om400636r.

[ref34] Whitaker D.; Burés J.; Larrosa I. Ag(I)-Catalyzed C–H Activation: The Role of the Ag(I) Salt in Pd/Ag-Mediated C–H Arylation of Electron-Deficient Arenes. J. Am. Chem. Soc. 2016, 138, 8384–8387. 10.1021/jacs.6b04726. 27303956

[ref35] Lotz M. D.; Camasso N. M.; Canty A. J.; Sanford M. S. Role of Silver Salts in Palladium-Catalyzed Arene and Heteroarene C–H Functionalization Reactions. Organometallics 2017, 36, 165–171.

[ref36] Wilkinson L. A.; Pike J. A.; Walton J. W. C-H Activation of pi-Arene Ruthenium Complexes. Organometallics 2017, 36, 4376–4381. 10.1021/acs.organomet.7b00563.

[ref37] Lee S. Y.; Hartwig J. F. Palladium-Catalyzed, Site-Selective Direct Allylation of Aryl C-H Bonds by Silver-Mediated C-H Activation: A Synthetic and Mechanistic Investigation. J. Am. Chem. Soc. 2016, 138, 15278–15284. 10.1021/jacs.6b10220. 27797512 PMC5161498

[ref38] Panigrahi A.; Whitaker D.; Vitorica-Yrezabal I. J.; Larrosa I. Ag/Pd Cocatalyzed Direct Arylation of Fluoroarene Derivatives with Aryl Bromides. ACS Catal. 2020, 10, 2100–2107. 10.1021/acscatal.9b05334. 32201633 PMC7079724

[ref39] Tlahuext-Aca A.; Lee S. Y.; Sakamoto S.; Hartwig J. F. Direct Arylation of Simple Arenes with Aryl Bromides by Synergistic Silver and Palladium Catalysis. ACS Catal. 2021, 11, 1430–1434. 10.1021/acscatal.0c05254. 34790433 PMC8594911

[ref40] Tlahuext-Aca A.; Hartwig J. F. Site-Selective Silver-Catalyzed C-H Bond Deuteration of Five-Membered Aromatic Heterocycles and Pharmaceuticals. ACS Catal. 2021, 11, 1119–1127. 10.1021/acscatal.0c04917. 35586574 PMC9113407

[ref41] Shimoyama Y.; Kuwabara J.; Kanbara T. Mechanistic Study of Pd/Ag Dual-Catalyzed Cross-Dehydrogenative Coupling of Perfluoroarenes with Thiophenes. ACS Catal. 2020, 10, 3390–3397. 10.1021/acscatal.9b05326.

[ref42] Li W.; Yuan D.; Wang G.; Zhao Y.; Xie J.; Li S.; Zhu C. Cooperative Au/Ag Dual-Catalyzed Cross-Dehydrogenative Biaryl Coupling: Reaction Development and Mechanistic Insight. J. Am. Chem. Soc. 2019, 141, 3187–3197. 10.1021/jacs.8b12929. 30681846

[ref43] Hu G. Q.; Li E. C.; Zhang H. H.; Huang W. Ag(I)-Mediated hydrogen isotope exchange of mono-fluorinated (hetero)arenes. Org. Biomol. Chem. 2020, 18, 6627–6633. 10.1039/D0OB01273D. 32832955

[ref44] Yao J.; Bai J.; Kang X.; Zhu M.; Guo Y.; Wang X. Non-directed C-H arylation of electron-deficient arenes by synergistic silver and Pd-3 cluster catalysis. Nanoscale 2023, 15, 3560–3565. 10.1039/D2NR05825A. 36723135

[ref45] Athavan G.; Tanner T. F. N.; Whitwood A. C.; Fairlamb I. J. S.; Perutz R. N. Direct Evidence for Competitive C-H Activation by a Well-Defined Silver XPhos Complex in Palladium-Catalyzed C-H Functionalization. Organometallics 2022, 41, 3175–3184. 10.1021/acs.organomet.2c00063.

[ref46] Carrow B. P.; Sampson J.; Wang L. Base-Assisted C-H Bond Cleavage in Cross-Coupling: Recent Insights into Mechanism, Speciation, and Cooperativity. Isr. J. Chem. 2020, 60, 230–258. 10.1002/ijch.201900095. 32669731 PMC7363398

[ref47] Bay K. L.; Yang Y. F.; Houk K. N. Multiple roles of silver salts in palladium-catalyzed C-H activations. J. Organomet. Chem. 2018, 864, 19–25. 10.1016/j.jorganchem.2017.12.026.

[ref48] Mudarra Á. L.; de Salinas S. M.; Pérez-Temprano M. H. Beyond the traditional roles of Ag in catalysis: the transmetalating ability of organosilver(I) species in Pd-catalysed reactions. Org. Biomol. Chem. 2019, 17, 1655–1667. 10.1039/C8OB02611D. 30474675

[ref49] Lee J. A.; Luscombe C. K. Dual-Catalytic Ag-Pd System for Direct Arylation Polymerization to Synthesize Poly(3-hexylthiophene). ACS Macro Lett. 2018, 7, 767–771. 10.1021/acsmacrolett.8b00429. 35650765

[ref50] Bhaskararao B.; Singh S.; Anand M.; Verma P.; Prakash P.; Athira C.; Malakar S.; Schaefer H. F.; Sunoj R. B. Is silver a mere terminal oxidant in palladium catalyzed C-H bond activation reactions?. Chem. Sci. 2020, 11, 208–216. 10.1039/C9SC04540F. 32110372 PMC7012060

[ref51] Kozitsyna N. Y.; Nefedov S. E.; Klyagina A. P.; Markov A. A.; Dobrokhotova Z. V.; Velikodny Y. A.; Kochubey D. I.; Zyubina T. S.; Gekhman A. E.; Vargaftik M. N.; Moiseev I. I. Novel heterometallic palladium-silver complex. Inorg. Chim. Acta 2011, 370, 382–387. 10.1016/j.ica.2011.02.003.

[ref52] Amatore C.; Carre E.; Jutand A.; M’barki M. A. Rates and mechanism of the formation of zerovalent palladium complexes from mixtures of Pd(OAc)_2_ and tertiary phosphines and their reactivity in oxidative additions. Organometallics 1995, 14, 1818–1826. 10.1021/om00004a039.

[ref53] Jeddi N.; Scott N. W. J.; Fairlamb I. J. S. Well-Defined Pd-n Clusters for Cross-Coupling and Hydrogenation Catalysis: New Opportunities for Catalyst Design. ACS Catal. 2022, 11615–11638. 10.1021/acscatal.2c03345.

[ref54] Wagschal S.; Perego L. A.; Simon A.; Franco-Espejo A.; Tocqueville C.; Albaneze-Walker J.; Jutand A.; Grimaud L. Formation of XPhos-Ligated Palladium(0) Complexes and Reactivity in Oxidative Additions. Chem. – Eur. J. 2019, 25, 6980–6987. 10.1002/chem.201900451. 30920694

[ref55] Montgomery M.; O’Brien H. M.; Mendez-Galvez C.; Bromfield C. R.; Roberts J. P. M.; Winnicka A. M.; Horner A.; Elorriaga D.; Sparkes H. A.; Bedford R. B. The surprisingly facile formation of Pd(I)-phosphido complexes from ortho-biphenylphosphines and palladium acetate. Dalton Trans. 2019, 48, 3539–3542. 10.1039/C8DT04926B. 30720818

[ref56] Bowmaker G. A.; Effendy; Hanna J. V.; Healy P. C.; King S. P.; Pettinari C.; Skelton B. W.; White A. H. Solution and mechanochemical syntheses, and spectroscopic and structural studies in the silver(I) (bi-)carbonate: triphenylphosphine system. Dalton Trans. 2011, 40, 7210–7218. 10.1039/c1dt10416k. 21660345

[ref57] Muetterties E. L.; Alegranti C. W. Solution Structure and Kinetic Study of Metal-Phosphine and Metal-Phosphite Complexes .1. Silver(I) System. J. Am. Chem. Soc. 1972, 94, 6386–6391. 10.1021/ja00773a022.

[ref58] Bachman R. E.; Andretta D. F. Metal-ligand bonding in coinage metal-phosphine complexes: The synthesis and structure of some low-coordinate silver(I)-phosphine complexes. Inorg. Chem. 1998, 37, 5657–5663. 10.1021/ic980726k. 11670715

[ref59] Patmore N. J.; Hague C.; Cotgreave J. H.; Mahon M. F.; Frost C. G.; Weller A. S. Silver phosphanes partnered with carborane monoanions: Synthesis, structures and use as highly active lewis acid catalysts in a hetero-Diels-Alder reaction. Chem. – Eur. J. 2002, 8, 2088–2098. 10.1002/1521-3765(20020503)8:9<2088::AID-CHEM2088>3.0.CO;2-L. 11981894

[ref60] Keller S.; Camenzind T. N.; Abraham J.; Prescimone A.; Haussinger D.; Constable E. C.; Housecroft C. E. Self-assembly of heteroleptic dinuclear silver(I) complexes bridged by bis(diphenylphosphino)ethyne. Dalton Trans. 2018, 47, 946–957. 10.1039/C7DT03923A. 29260149

[ref61] Camalli M.; Caruso F. Correlation between P-31 NMR Data and Structural Parameters on Ag(PPh_3_)_3_ Series - Crystal and Molecular-Structure of Tris(Triphenylphosphine)Silver(I)Tetrafluoroborate and Tris(Triphenylphosphine)Silver(I)Iodide. Inorg. Chim. Acta 1987, 127, 209–213. 10.1016/S0020-1693(00)82122-2.

[ref62] Effendy; Lobbia G. G.; Pellei M.; Pettinari C.; Santini C.; Skelton B. W.; White A. H. Solution and solid-state structural properties of silver(I) dihydrobis(pyrazolyl)borate compounds with tertiary mono(phosphine) ligands. J. Chem. Soc., Dalton Trans. 2000, 2123–2129. 10.1039/b001168l.

[ref63] Cingolani A.; Effendy; Marchetti F.; Pettinari C.; Skelton B. W.; White A. H. Synthesis and structural systematics of mixed triphenylphosphine/imidazole base adducts of silver(I) oxyanion salts. J. Chem. Soc., Dalton Trans. 1999, 4047–4055. 10.1039/a906624a.

[ref64] Ratai E. M.; Pilkenton S.; Lentz M. R.; Greco J. B.; Fuller R. A.; Kim J. P.; He J.; Cheng L. L.; Gonzalez R. G. Comparisons of brain metabolites observed by HRMAS H-1 NMR of intact tissue and solution H-1 NMR of tissue extracts in SIV-infected macaques. NMR Biomed. 2005, 18, 242–251. 10.1002/nbm.953. 15759297

[ref65] Griffin J. L.; Bollard M.; Nicholson J. K.; Bhakoo K. Spectral profiles of cultured neuronal and glial cells derived from HRMAS H-1 NMR spectroscopy. NMR Biomed. 2002, 15, 375–384. 10.1002/nbm.792. 12357551

[ref66] Chen J. H.; Sambol E. B.; DeCarolis P.; O’Connor R.; Geha R. C.; Wu Y. V.; Singer S. High-resolution MAS NMR spectroscopy detection of the spin magnetization exchange by cross-relaxation and chemical exchange in intact cell lines and human tissue specimens. Magn. Reson. Med. 2006, 55, 1246–1256. 10.1002/mrm.20889. 16676334

[ref67] Mastikhin V. M. Characterization of surface-active sites of catalysts with high-resolution solid-state nmr. Colloids Surf., A 1993, 78, 143–166. 10.1016/0927-7757(93)80320-E.

[ref68] Posset T.; Blumel J. New mechanistic insights regarding Pd/Cu catalysts for the Sonogashira reaction: HRMAS NMR studies of silica-immobilized systems. J. Am. Chem. Soc. 2006, 128, 8394–8395. 10.1021/ja062206r. 16802793

[ref69] Roy A. D.; Jayalakshmi K.; Dasgupta S.; Roy R.; Mukhopadhyay B. Real time HR-MAS NMR: application in reaction optimization, mechanism elucidation and kinetic analysis for heterogeneous reagent catalyzed small molecule chemistry. Magn. Reson. Chem. 2008, 46, 1119–1126. 10.1002/mrc.2321. 18853391

[ref70] Gauniyal H. M.; Gupta S.; Sharma S. K.; Bajpai U. Temperature-Gradient-Directed NMR Monitoring of a 3+3 -Cyclocondensation Reaction Between Alkynone and Ethyl 2-Amino-1H-indole-3-carboxylate Toward the Synthesis of Pyrimido 1,2-a indole Catalyzed by Cs_2_CO_3_. Synth. Commun. 2013, 43, 2090–2099. 10.1080/00397911.2012.687423.

[ref71] Pinoie V.; Poelmans K.; Miltner H. E.; Verbruggen I.; Biesemans M.; Van Assche G.; Van Mele B.; Martins J. C.; Willem R. A polystyrene-supported tin trichloride catalyst with a C11-spacer. Catalysis monitoring using high-resolution magic angle spinning NMR. Organometallics 2007, 26, 6718–6725. 10.1021/om7008638.

[ref72] Chen F.; Min Q. Q.; Zhang X. Pd-catalyzed direct arylation of polyfluoroarenes on water under mild conditions using PPh_3_ ligand. J. Org. Chem. 2012, 77, 2992–2998. 10.1021/jo300036d. 22339158

[ref73] Garcia-Melchor M.; Braga A. A. C.; Lledos A.; Ujaque G.; Maseras F. Computational Perspective on Pd-Catalyzed C-C Cross-Coupling Reaction Mechanisms. Acc. Chem. Res. 2013, 46, 2626–2634. 10.1021/ar400080r. 23848308

[ref74] Salamanca V.; Albéniz A. C. Deuterium Exchange between Arenes and Deuterated Solvents in the Absence of a Transition Metal: Synthesis of D-Labeled Fluoroarenes. Eur. J. Org. Chem. 2020, 2020, 3206–3212. 10.1002/ejoc.202000284.

[ref75] Fischmeister C.; Doucet H. Greener solvents for ruthenium and palladium-catalysed aromatic C-H bond functionalisation. Green Chem. 2011, 13, 741–753. 10.1039/c0gc00885k.

[ref76] Reay A. J.; Fairlamb I. J. S. Catalytic C-H bond functionalisation chemistry: the case for quasi-heterogeneous catalysis. Chem. Commun. 2015, 51, 16289–16307. 10.1039/C5CC06980G. 26439875

[ref77] Cao M.; Wu D.; Su W.; Cao R. Palladium nanocrystals stabilized by cucurbit 6 uril as efficient heterogeneous catalyst for direct C-H functionalization of polyfluoroarenes. J. Catal. 2015, 321, 62–69. 10.1016/j.jcat.2014.10.013.

[ref78] Hansch C.; Leo A.; Taft R. W. A survey of Hammett substituent constants and resonance and field parameters. Chem. Rev. 1991, 91, 165–195. 10.1021/cr00002a004.

[ref79] Yau H. M.; Croft A. K.; Harper J. B. ’One-pot’ Hammett plots: a general method for the rapid acquisition of relative rate data. Chem. Commun. 2012, 48, 8937–8939. 10.1039/c2cc34074g. 22847368

[ref80] Sun H.-Y.; Gorelsky S. I.; Stuart D. R.; Campeau L.-C.; Fagnou K. Mechanistic Analysis of Azine N-Oxide Direct Arylation: Evidence for a Critical Role of Acetate in the Pd(OAc)_2_ Precatalyst. J. Org. Chem. 2010, 75, 8180–8189. 10.1021/jo101821r. 21053903

[ref81] Edwards D. A.; Harker R. M.; Mahon M. F.; Molloy K. C. Aerosol-assisted chemical vapour deposition (AACVD) of silver films from triorganophosphine adducts of silver carboxylates, including the structure of [Ag(O_2_CC_3_F_7_)(PPh_3_)_2_]. Inorg. Chim. Acta 2002, 328, 134–146. 10.1016/S0020-1693(01)00718-6.

[ref82] Soltys-Brzostek K.; Terlecki M.; Sokolowski K.; Lewinski J. Chemical fixation and conversion of CO2 into cyclic and cage-type metal carbonates. Coord. Chem. Rev. 2017, 334, 199–231. 10.1016/j.ccr.2016.10.008.

[ref83] Tyrra W.; Wickleder M. S. Silver compounds in synthetic chemistry. 1 - A facile preparative route for pentafluorophenylsilver, AgC_6_F_5_ and its use as an oxidative pentafluorophenyl group transfer reagent in reactions with group 12 to 16 elements - the single crystal structure of AgC_6_F_5_. EtCN, the first arylsilver derivative crystallising in infinite chains. Z. Anorg. Allg. Chem. 2002, 628, 1841–1847.

[ref84] Edwards D. A.; Harker R. M.; Mahon M. F.; Molloy K. C. Crystallographic analysis of an intramolecularly stabilised organosilver tetramer, [Ag(C_6_H_4_NMe_2_-2)]_4_, and the first structurally characterised silver siloxide, {[Ag(PPh_3_)]_2_-(μ^4^-OMe_2_SiOSiMe_2_O)}_2_. J. Chem. Soc., Dalton Trans. 1997, 3509–3513. 10.1039/a704205a.

[ref85] Meyer E. M.; Gambarotta S.; Floriani C.; Chiesivilla A.; Guastini C. Polynuclear Aryl Derivatives of Group-11 Metals - Synthesis, Solid-State Solution Structural Relationship, and Reactivity with Phosphines. Organometallics 1989, 8, 1067–1079. 10.1021/om00106a031.

[ref86] Voelker H.; Labahn D.; Bohnen F. M.; Herbst-Irmer R.; Roesky H. W.; Stalke D.; Edelmann F. T. Structural diversity in nonafluoromesityl chemistry. New J. Chem. 1999, 23, 905–909. 10.1039/a903798e.

[ref87] Linden H. B. Liquid injection field desorption ionization: a new tool for soft ionization of samples including air-sensitive catalysts and non-polar hydrocarbons. Eur. J. Mass Spectrom. 2004, 10, 459–468. 10.1255/ejms.655. 15302970

[ref88] Dransfield T. A.; Nazir R.; Perutz R. N.; Whitwood A. C. Liquid injection field desorption/ionization of transition metal fluoride complexes. J. Fluorine Chem. 2010, 131, 1213–1217. 10.1016/j.jfluchem.2010.05.008.

[ref89] Wenzel M. N.; Owens P. K.; Bray J. T. W.; Lynam J. M.; Aguiar P. M.; Reed C.; Lee J. D.; Hamilton J. F.; Whitwood A. C.; Fairlamb I. J. S. Redox Couple Involving NO_x_ in Aerobic Pd-Catalyzed Oxidation of sp^3^-C-H Bonds: Direct Evidence for Pd-NO_3_^-^/NO_2_^-^ Interactions Involved in Oxidation and Reductive Elimination. J. Am. Chem. Soc. 2017, 139, 1177–1190. 10.1021/jacs.6b10853. 28075565

[ref90] Scott N. W. J.; Ford M. J.; Husbands D. R.; Whitwood A. C.; Fairlamb I. J. S. Reactivity of a Dinuclear Pd-I Complex [Pd_2_(μ-PPh_2_)(μ^2^-OAc)(PPh_3_)_2_] with PPh_3_: Implications for Cross-Coupling Catalysis Using the Ubiquitous Pd(OAc)_2_/nPPh_3_ Catalyst System. Organometallics 2021, 40, 2995–3002. 10.1021/acs.organomet.1c00347. 34539028 PMC8441971

[ref91] Rosner T.; Le Bars J.; Pfaltz A.; Blackmond D. G. Kinetic studies of Heck coupling reactions using palladacycle catalysts: Experimental and kinetic modeling of the role of dimer species. J. Am. Chem. Soc. 2001, 123, 1848–1855. 10.1021/ja003191e. 11456804

[ref92] Kozuch S.; Shaik S. How to Conceptualize Catalytic Cycles? The Energetic Span Model. Acc. Chem. Res. 2011, 44, 101–110. 10.1021/ar1000956. 21067215

