# Supplementary material for: Opening a Pandora’s Flask on a Prototype Catalytic Direct Arylation Reaction of Pentafluorobenzene: The Ag2CO3/Pd(OAc)2/PPh3 System
Source: Organometallics. 2023 Aug 30;42(17):2378–94. doi: 10.1021/acs.organomet.3c00309 (PMC10498494; doi:10.1021/acs.organomet.3c00309)
Supplement: Supplementary file 1 — om3c00309_si_001.pdf [file om3c00309_si_001.pdf]

Supporting information

Opening a Pandora's flask on a Prototype Catalytic Direct Arylation Reaction of Pentafluorobenzene: the Ag_2CO_3/$Pd(OAc)_2$/PPh_3 system

George M. H. Platt, Pedro M. Aguiar, Gayathri Athavan, Joshua T. W. Bray, Neil W. J. Scott, Ian J. S. Fairlamb* and Robin N. Perutz*

Department of Chemistry, University of York, York YO10 5DD, United Kingdom

Corresponding authors:
ian.fairlamb@york.ac.uk
robin.perutz@york.ac.uk

Contents

Opening a Pandora's flask on a Prototype Catalytic Direct Arylation Reaction of Pentafluorobenzene: the Ag_2CO_3/$Pd(OAc)_2$/PPh_3 system 1

1. General Information on Chemicals and Instruments 3
   1.1 Solvents and Reagents 3
   1.2 Instrumentation 3
2. General Procedures 4
   2.1 Synthesis of Fluorinated Biaryls (General procedure A) 4
   2.2 *In situ* FT-IR Spectroscopy to Monitor the Kinetics of a Reaction Initiated by the Addition of 1 (General procedure C) 4
   2.3 In situ FT-IR Spectroscopy to Monitor the Kinetics of a Reaction Initiated by Mixing the Liquid and Solid Reagents of the Reaction (General procedure D) 4
3. Synthetic Procedures and Compound Data 5
4. Studies of conversions for coupling of 1 and 2a 16
5. Speciation of silver complexes 19
   5.1 General Procedure for reactions of Ag^I salts in acetonitrile 19
   5.2 The reaction of Ag_2CO_3 and PPh_3 with C_6F_5H in DMF at 60 °C 20
   5.3 Reactions with Phosphine-coordinated Silver(I) complexes 20
   5.4 Reactions with Catalytic Amounts of Ag^I salt 22
6. Analysis of Catalytic Intermediates by in situ HR-MAS NMR Spectroscopy 23
   6.1 Direct Arylation of 4-Iodotoluene 2a with 1 catalysed by $Pd(OAc)_2$ 23
   6.2 Direct Arylation of Iodobenzene 2b with 1 catalysed by $Pd(OAc)_2$ 24
   6.3 Direct Arylation of Iodobenzene 2b with 1 Catalysed by $[Pd(Ph)(\mu\text{-}OAc)(PPh_3)]_2$ 24
   6.4 Characterisation of Pd intermediates by HR-MAS NMR and by LIFDI mass spectometry 24
7. Reaction kinetics by in-situ IR spectroscopy 33
   7.1 Variable Time Normalisation Analysis of the Direct Arylation of 4-Iodotoluene 2a with 1 35

7.2 Variable Time Normalisation Analysis 36

7.3 Kinetic Analysis of the Direct Arylation of 4-Iodotoluene 2a with 1 by Isolation Method39

7.4 Kinetic Analysis of the Direct Arylation of Iodobenzene 2b with 1 by Isolation Method .45

7.5 Kinetic Analysis of the Direct Arylation of 4-Iodotoluene 2b with 1 using Different Quantity of PPh_3 47

7.6 Kinetic Analysis of the Direct Arylation of 4-Substituted-Iodobenzenes with 1 48

7.7 Kinetic analysis with alternative catalyst 49

7.8 Kinetic Isotope Effect of the Direct Arylation of 4-Iodotoluene 2b with Deuteropentafluorobenzene 1-d...52

7.9 Temperature Dependence of the Direct Arylation of 4-Iodotoluene 2a with 1 52

8. References 53

1. General Information on Chemicals and Instruments

1.1 Solvents and Reagents

Unless stated otherwise, all reagents were purchased from commercial sources (Acros Organics, Alfa Aesar, Fischer Scientific, Fluorochem, Sigma-Aldrich or TCI) and used without further purification. $Pd(OAc)_2$ was purchased from Precious Metals Online. PPh_3 was recrystallized from ethanol and dried over P_2O_5. Iodobenzene, pentafluorobenzene and pentafluorobromobenzene were degassed by three cycles of freeze-pump-thaw and stored over 3 Å molecular sieves. Extra dry DMF (99.8%, water < 50 ppm) purchased from Acros Organics was degassed by bubbling with nitrogen gas under sonication for 30 min and stored in an ampoule over 3 Å molecular sieves. The water content of the DMF was determined using Mettler Toledo DL32 Karl Fischer Coulometer (from Fischer Scientific, UK) with double platinum pin electrodes. Other dry solvents (*e.g.* benzene, methanol) were collected from the solvent purification system and distilled under argon. Chloroform-d was dried by stirring over CaH_2 for 24 h, degassed by three cycles of freeze-pump-thaw and distilled. The synthesised Pd complexes were stored at −20 °C in the freezer inside the glove box.

Air and moisture sensitive procedures were carried out using oven- or flame-dried glassware, inside dry argon filled glove box (< 0.5 ppm O_2) or using standard Schlenk line (10^{-2} mbar) techniques connected to argon or nitrogen gas. Nitrogen gas from the liquid nitrogen tank was dried by passing through a column of sodium hydroxide pellets and silica prior to use.

Merck aluminium backed thin-layer chromatography (TLC) plates with silica gel 60 F254 were used for TLC. Retention factors (R_f) are reported to two decimal places Fluka 60 silica gel (35–75 µm particle size) was used for flash column chromatography.

1.2 Instrumentation

Solution NMR spectra were recorded using Jeol ECX400 MHz or ECS400 MHz (^{1}H 400 MHz; ^{13}C 101 MHz; ^{19}F 376 MHz; ^{31}P 162 MHz) spectrometers or Bruker AMX 500 (^{1}H 500 MHz; ^{2}H 77 MHz; ^{13}C 126 MHz; ^{19}F 471 MHz; ^{31}P 202 MHz) spectrometer. Solution phase NMR of suspensions (*i.e.* HR-MAS NMR) were recorded using a Bruker Avance III HD spectrometer equipped with 4 mm MAS BB/BB/1H/19F H8906–20/0003. The spectra were measured at rotation frequencies of 3 kHz. Residual protio solvent resonances of DMF-d_7 (δ_H 8.03), benzene-d_6 (δ_H 7.16), chloroform-d (δ_H 7.26) and dichloromethane-d_2 (δ_H 5.32) were used as internal reference. The residual deutero solvent resonance of benzene (δ_H 7.26) was used as internal reference for deuterium (^{2}H) NMR spectras. Carbon-13 (^{13}C) NMR spectra were internally referenced to residual solvent resonances of chloroform-d (δ_C 77.2) and benzene-d_6 (δ_C 128.1). Fluorine-19 (^{19}F{^{1}H}) NMR spectra were externally referenced to $CFCl_3$. Phosphorus-31 (^{31}P{^{1}H}) NMR spectra were externally referenced to H_3PO_4

Electrospray ionisation (ESI) mass spectra were measured using a Bruker micrOTOF spectrometer with *m/z* values accurate to four decimal places. Electrospray spectrometry (EI) mass spectrometry and liquid injected field desorption ionization (LIFDI) mass spectrometry were measured using Waters GCT Premier mass spectrometer with *m/z* values were accurate to two decimal places. For compounds with multiple isotopic peaks, the most abundant ion was reported. Isotope distribution plots were made using OriginPro™ 2016.

Solid phase IR spectra were recorded by means of a KBr disc, and liquid phase IR spectra in a CaF_2 solution cell using a Unicam Research Series FT-IR spectrometer. ATR spectra were recorded using an ALPHA FT-IR spectrometer. *In situ* FT-IR spectra were recorded using ReactIR™ iC10 (from Mettler-Toledo) fitted with a K6 Conduit (16 mm probe) and a SiComp silicon ATR sensor. Spectra were

collected every minute between 4000–650 cm^{-1} at resolution of 4 cm^{-1} with 167 scans min^{-1}. Data were analysed using MC-IR™ version 4.0 and Microsoft Excel 2010. The results were presented using Origin Pro 2016.

Reaction temperatures were monitored with a Tenma 72-7715 (from Farnell, UK) thermometer connected to a K-type thermocouple with 0.2% ± 0.6 °C accuracy calibrated in an ice bath at 0 °C. The thermocouple was submerged in the solvent.

Elemental (CHN) analysis was run on Exeter Analytical CE-440 Elemental Analyser. The percentages were an average of three runs and recorded to two decimal places.

2. General Procedures

2.1 Synthesis of Fluorinated Biaryls (General procedure A)

A light-protected Schlenk tube with Ag_2CO_3 (0.186 g, 0.68 mmol, 0.75 equiv.), PPh_3 (24 mg, 0.092 mmol, 10 mol%) and $Pd(OAc)_2$ (10 mg, 0.045 mmol, 5 mol%) was evacuated and backfilled with atmosphere of nitrogen three times. To this, haloarene (0.9 mmol, 1 equiv.) was added against the flow of nitrogen, followed by the fluoroarene (1.35–2.70 mmol, 1.5–3 equiv.) and 2.5 cm^3 DMF. The mixture was heated at 70 °C for 24 h under nitrogen. After the crude mixture had cooled to ambient temperature, it was extracted with EtOAc (80 cm^3) and deionized water (40 cm^3). The aqueous phase was back-extracted with EtOAc (2 × 40 cm^3), dried over Na_2SO_4, filtered and solvent removed under vacuum to give a crude product.

2.2 *In situ* FT-IR Spectroscopy to Monitor the Kinetics of a Reaction Initiated by the Addition of 1 (General procedure C)

A light-protected three-necked 100 cm^3, round-bottom flask equipped with a stir bar was fitted to the IR probe with the second neck connected to the Schlenk line and the third neck sealed with a rubber septum. The flask was evacuated and refilled with nitrogen (× 3) before configuring the probe under nitrogen. The IR instrument was set up by collecting second atmosphere background, followed by the solvent background of DMF. Data collection was started immediately after the set up. Reagents were added in the order of iodoarene, PPh_3, $Pd(OAc)_2$, and Ag_2CO_3 with 30 min gap between the addition of $Pd(OAc)_2$ and Ag_2CO_3. The mixture was heated in a pre-heated oil bath at 60 °C. The reaction was initiated by the addition of pentafluorobenzene **56**. The total volume of the mixture was maintained at 9.7 cm^3. The reaction mixture was stabilised at 56 ± 1 °C using the digital thermometer, and stirred at a constant rate under a continuous flow of nitrogen. The reaction progress was monitored following the IR signals of iodobenzene **2b** (1016 cm^{-1} and 998 cm^{-1}), 4-iodotoluene **2a** (1009 cm^{-1}), product **3a** (989 cm^{-1}) and pentafluorobenzene **1** (957 cm^{-1} and 944 cm^{-1}).

2.3 In situ FT-IR Spectroscopy to Monitor the Kinetics of a Reaction Initiated by Mixing the Liquid and Solid Reagents of the Reaction (General procedure D)

A light-protected three-necked 100 cm^3, round-bottom flask equipped with a stir bar was fitted to the IR probe with the second neck connected to the Schlenk line and the third neck sealed with a rubber septum. The round-bottom flask was charged with the solid reagents (*e.g.* pre-catalyst, Ag_2CO_3 and ligand). The flask was evacuated and refilled with nitrogen (× 3) before configuring the probe and starting the data collection. A DMF (8.0 cm^3) solution of iodobenzene **2b** and

pentafluorobenzene **1** in a Schlenk tube was transferred into the round-bottom flask using a cannula. The Schlenk tube and the needle were rinsed with DMF, making the total volume of the mixture to 9.76 cm^3. The reaction was heated in a pre-heated oil bath at 60 °C and stirred at a constant rate under continuous flow of nitrogen. The temperature of the reaction mixture was stabilised at 56 ± 1 °C after *ca.* 10 min monitoring using the digital thermometer. The reaction progress was monitored following the IR signals as above.

3. Synthetic Procedures and Compound Data

The following palladium complexes were synthesised by literature procedures: bis(triphenylphosphine)palladium(dichloride),[1] tetrakis(triphenylphosphine)palladium),[2] di(μ-hydroxo)-bis(phenyl)-di(triphenylphosphine)palladium(II), $[Pd(C_6H_5)(\mu\text{-}OH)(PPh_3)]_2$ a 1:3 mixture of *cis* and *trans* isomers,[3] di(μ-hydroxo)-bis(4-tolyl)-di(triphenylphosphine)palladium(II) dimer, $[Pd(4\text{-}tolyl)(\mu\text{-}OH)(PPh_3)]_2$ 1:4 mixture of *cis-trans* isomers,[3] di(μ-acetato)-bis(phenyl)-di(triphenylphosphine)palladium(II) dimer, $[Pd(Ph)(\mu\text{-}OAc)(PPh_3)]_2$,[4] di($\mu$-acetato)-bis(4-tolyl)-di(triphenylphosphine)palladium(II) dimer, $[Pd(4\text{-}tolyl)(\mu\text{-}OAc)(PPh_3)]_2$,[4] di($\mu$-iodo)-bis(phenyl)-di(triphenylphosphine)palladium(II) dimer, $[Pd(Ph)(\mu\text{-}I)(PPh_3)]_2$,[3] iodo-bis(triphenylphosphine)-(phenyl)palladium(II), $Pd(Ph)(I)(PPh_3)_2)$,[5] iodo-bis(triphenylphosphine)-(4-tolyl)palladium(II), $Pd(4\text{-}tolyl)(I)(PPh_3)_2$,[6] bis(triphenylphosphine)-(phenyl)palladium(II)acetate, $Pd(Ph)(\kappa^1\text{-}OAc)(PPh_3)_2$.[4]

The following silver complexes were synthesised by literature procedures: $Ag(C_6F_5)$,[7] $Ag(PPh_3)_2(\kappa^2\text{-}OAc)$,[8] $\{Ag(PPh_3)_2(\kappa^2\text{-}HCO_3)\}_2$,[9] $\{Ag(PPh_3)_2\}_2(\mu\text{-}\kappa^2,\kappa^1\text{-}CO_3)$.[9]

2,3,4,5,6-pentafluoro-4′-(methyl)biphenyl (3a)

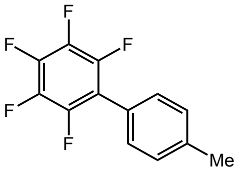

Pentafluorobenzene **1** (150 µL, 227 mg, 1.35 mmol, 1.5 equiv.) and 4-iodotoluene **2a** (196 mg, 0.899 mmol, equiv.) were reacted following the general procedure A. Purification by flash chromatography (SiO_2, R_f = 0.53, petroleum-ether 40–60 °C) gave the product **58** (195 mg, 84%) as a white crystalline solid; m.p. 118.9–119.4 °C; ν_{max}/cm^{-1} (disc, KBr) 3043 (C–H aromatic str.), 2927 (CH_3 str.), 1514 (–CH=CH– str.), 1491 (C=C aromatic str.), 1062 (C–F str.), 988 (=C–H in-plane bend), 821 (=C–H oop bend); ^1H NMR (400 MHz, 16 scans, C_6D_6): δ = 7.10 (2 H, d, *J* = 8.0 Hz, 2′,6′-H), 6.97 (2 H, d, *J* = 8.0 Hz, 3′,5′-H), 2.06 (3 H, s, 4′-CH_3); ^{13}C NMR (100 MHz, 1024 scans, $CDCl_3$): δ = 144.3 (dm, *J* = 246.5 Hz, CF), 140.4 (dm, *J* = 233.5, 4-CF), 139.6 (s), 138.0 (dm, *J* = 250.5 Hz, CF), 130.1 (dt, *J* = 2.0, 1.0 Hz, 2′,6′-C), 129.6 (s, 3′,5′-C), 123.6–123.5 (m), 116.1 (dt, *J* = 16.5, 3.5 Hz), 21.5 (s, CH_3); ^{19}F NMR (376 MHz, 64 scans, C_6D_6): δ = −143.50 (2F, dd, *J* = 23.5, 8.0 Hz, 2,6-F), −156.20 (1 F, t, *J* = 21.5 Hz, 4-F), −162.27 (2 H, ddd, *J* = 23.5, 21.5, 8.0 Hz, 3,5-F); MS (EI) *m/z* (%) 258.0461 ($[M]^+$, 100) ($C_{13}H_7F_5$ requires 258.0468).

The analytical data obtained were in agreement with the literature.[10]

(*Lab book reference number*: GMHP-1-10)

4,4′-(dimethyl)biphenyl

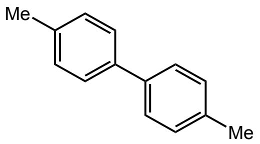

A mixture of 4-iodotoluene **2a** (437 mg, 2.0 mmol, 1 equiv.), $Pd(OAc)_2$ (22 mg, 5 mol%) PPh_3 (52 mg, 10 mol%), Ag_2CO_3 (607 mg, 2.2 mmol, 1.1 equiv.) and benzoin (255 mg, 1.2 mmol, 0.6 equiv.) in 3 cm^3 DMF was heated at 120 °C for 5 hours under atmosphere of argon. The reaction mixture rapidly changed colour from yellow to black upon heating. The crude mixture was worked-up as described in the general procedure. Purification by flash chromatography (SiO_2, R_f = 0.21, petroleum-ether 40–60 °C) gave the product **55** (77 mg, 42%) as a white solid; m.p. 104.6–106.0 °C (lit.[11] 119–120 °C); ν_{max}/cm^{-1} (disc, KBr) 3029 (C–H aromatic str.), 2918 (CH_3 str.), 1510 (–CH=CH– str.), 808 (=C–H oop bend); ^1H NMR (400 MHz, 32 scans, CD_2Cl_2): δ = 7.49 (4 H, d, *J* = 8.0 Hz, 2,2′,6,6′-H), 7.25 (4 H, d, *J* = 8.0 Hz, 3,3′,5,5′-H), 2.39 (6 H, s, CH_3); ^{13}C NMR (100 MHz, 1024 scans, CD_2Cl_2): δ =138.6 (s), 137.4 (s), 130.0 (s, 3,3′,5,5′-H), 127.1 (s, 2,2′,6,6′-CH), 21.3 (s, CH_3); MS (EI) *m/z* (%) 182.1102 ($[M]^+$, 100) ($C_{14}H_{14}$ requires 182.1096).

A literature procedure was followed for the synthesis of this compound.[12] The analytical data obtained were in agreement with the literature.

(*Lab book reference number*: GMHP-2-69, GMHP-2-71)

2,3,4,5,6-pentafluorobiphenyl (3b)

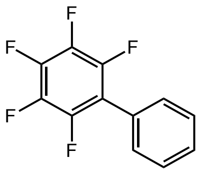

Pentafluorobenzene **1** (150 µL, 227 mg, 1.35 mmol, 1.5 equiv.) and iodobenzene **22b** (100 µL, 0.894 mmol, 1 equiv.) were reacted following the general procedure A. Purification by flash chromatography (SiO_2, R_f = 0.51, petroleum-ether 40–60 °C) gave the product **3b** (182 mg, 84%) as a white crystalline solid; m.p. 111.6–112.3 °C (lit.[13] 111–112 °C); ν_{max}/cm^{-1} (disc, KBr) 3054 (C–H aromatic str.), 2987 (CH_3 str.), 1526 (–CH=CH– str.), 1496 (C=C aromatic str.), 1063 (C–F str.), 984 (=C–H in-plane bend), 853 (=C–H oop bend); ^1H NMR (400 MHz, 8 scans, $CDCl_3$): δ = 7.53–7.45 (3 H, m, 2′,4′,6′-H), 7.44–7.41 (2H, m, 3′,5′-H); ^{13}C NMR (100 MHz, 1024 scans, $CDCl_3$): δ = 144.3 (dm, *J* = 247.5 Hz, CF), 140.5 (dm, *J* = 248.5 Hz, 4-CF), 138.0 (dm, *J* =250.5 Hz, CF), 130.3 (t, *J* = 1.5 Hz, 3′,5′-CH), 129.5 (s, 4′-H), 128.9 (s, 2′,6′-H), 126.5 (q, *J* = 2.0 Hz), 116.1 (td, *J* = 17.5, 4.0 Hz); ^{19}F NMR (376 MHz, 64 scans, $CDCl_3$): δ = −143.17 (2 F, dd, *J* = 23.0, 8.0 Hz, 2,6-F), −155.53 (1 F, t, *J* = 21.0 Hz, 4-F), −162.09 to −162.24 (2 F, m, 3,5-F); MS (EI) *m/z* (%) 244.0311 ($[M]^+$, 100) ($C_{12}H_5F_5$ requires 244.0311).

The analytical data obtained were in agreement with the literature.[14]

(*Lab book reference number*: GMHP-3-158)

2,3,5,6-tetrafluoro-4′-(methyl)biphenyl

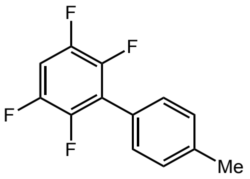

The reaction of 1,2,4,5-tetrafluorobenzene (300 µL, 403 mg, 2.69 mmol, 3 equiv.) and 4-iodotoluene **2b** (197 mg, 0.904 mmol, 1 equiv.) was prepared following the general procedure A. Purification by flash chromatography (SiO_2, R_f =0.33, petroleum-ether 40–60 °C) gave the major product (126 mg, 58%) as a white solid; m.p. 85.2–86.6 °C (lit.[15] 88–89 °C); ν_{max}/cm^{-1} (disc, KBr) 3078 (C–H aromatic str.), 2926 (CH_3 str.), 1502 (–CH=CH– str.), 1490 (C=C aromatic str.), 1175 (C–F str.), 930 (=C–H in-plane bend), 814 (=C–H oop bend); ^{1}H NMR (400 MHz, 8 scans, $CDCl_3$): δ = 7.36 (2 H, d *J* = 8.5 Hz, 2′,6′-H), 7.31 (2 H, d, *J* = 8.0 Hz, 3′,5′-H), 7.05 (1 H, tt, *J* = 9.5, 7.5 Hz, 4-H), 2.43 (3 H, s, CH_3); ^{13}C NMR (100 MHz, 1024 scans, $CDCl_3$): δ = 146.4 (dm, *J* = 247.0 Hz, CF), 144.0 (dm, *J* = 242.0 Hz, CF), 139.4 (s), 130.1 (t, *J* = 2.0 Hz, 2′,6′-C), 129.5 (s, 3′,5′-C), 124.6 (t, *J* = 2.5 Hz), 121.7 (t, *J* = 17.0 Hz), 104.4 (tt, *J* = 22.5, 0.5 Hz), 21.5 (s, CH_3); ^{19}F NMR (376 MHz, 64 scans, $CDCl_3$): δ = −139.25 (2 F, ddd, *J* = 22.5, 13.0, 9.5 Hz, 2,6-F), −143.86 to −143.97 (2 F, m, 3,5-F); MS (EI) *m/z* (%) 240.0568 ([M]$^+$, 100) ($C_{13}H_8F_4$ requires 240.0562).

The analytical data obtained were in agreement with the literature.[15]

(*Lab book reference number*: GMHP-3-128)

1,4-di(4′,4′′-tolyl)-2,3,5,6-tetrafluorobenzene

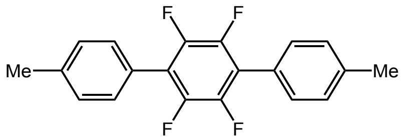

The compound was obtained as the minor product in the 2,3,5,6-tetrafluoro-4′-(methyl)biphenyl reaction. Purification by flash column chromatography (SiO_2, R_f = 0.17, petroleum-ether 40–60 °C) gave the product (33 mg, 11%) as a white solid; m.p. 221.3–222.9 °C (lit.[15] 210–212 °C); ν_{max}/cm^{-1} (disc, KBr) 3041 (C–H aromatic str.), 2926 (CH_3 str.), 1475 (C=C aromatic str.), 1193 (C–F str.), 971 (=C–H in-plane bend), 818 (=C–H oop bend); ^{1}H NMR (400 MHz, 16 scans, CD_2Cl_2): δ = 7.41 (4 H, d, *J* = 8.0 Hz, 2′,2′′,6′,6′′-H), 7.34 (4 H, d, *J* = 8.0 Hz, 3′,3′′,5′,5′′-H), 2.43 (6 H, s, CH_3); ^{19}F NMR (376 MHz, 64 scans, CD_2Cl_2): δ = −145.35 (4 F, s, 2,3,5,6-F); MS (EI) *m/z* (%) 330.1032 ([M]$^+$, 100) ($C_{20}H_{14}F_4$ requires 330.1032).

The analytical data obtained were in agreement with the literature.[15]

(*Lab book reference number*: GMHP-3-128)

2,3,5,6-tetrafluoro-4,4′-(dimethyl)biphenyl

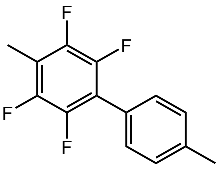

The reaction of 2,3,5,6-tetrafluorotoluene (165 µL, 222 mg, 1.35 mmol, 1.5 equiv.) and 4-iodotoluene **2b** (198 mg, 0.908 mmol, 1 equiv.) was prepared following the general procedure A. Purification by flash chromatography (SiO_2, R_f =0.38, petroleum-ether 40–60 °C) gave the product (214 mg, 93%) as a white crystalline solid; m.p. 65.3–66.6 °C (lit.[15] 67–68 °C); ν_{max}/cm^{-1} (ATR) 2924 (CH_3 str.), 1473 (C=C aromatic str.), 1062 (C–F str.), 921 (=C–H in-plane bend), 813 (=C–H oop bend); ^1H NMR (400 MHz, 16 scans, C_6D_6): δ = 7.27 (2 H, dt, *J* = 6.5, 1.4 Hz, 2′,6′-H), 6.99 (2 H, d, *J* = 8.0 Hz 3′,5′-H), 2.07 (3 H, s, 4′-CH_3), 1.81 (3 H, t, *J* = 2.0 Hz, 4-CH_3); ^{13}C NMR (100 MHz, 1024 scans, $CDCl_3$): δ = 145. 4 (dddd, *J* = 244.0, 14.5, 7.5, 4.0 Hz, CF), 143.7 (dddd, *J* = 245.5, 14.5, 5.5, 4.0 Hz, CF), 139.1 (s), 130.1 (t, *J* = 2.0 Hz, 2′,6′-C), 129.4 (s, 3′,5′-C), 124.9 (t, *J* = 2.5 Hz), 118.1 (t, *J* = 17.0 Hz), 114.9 (t, *J* = 19.5 Hz), 21.4 (s, 4′-CH_3), 7.6 (tt, *J* = 3.5, 2.0 Hz, 4-CH_3); ^{19}F NMR (376 MHz, 64 scans, C_6D_6): δ = −144.45 (2 H, ddq, *J* = 23.0, 13.0, 2.0 Hz, 3,5-F), −145.81 (2 H, dd, *J* = 22.5, 12.5 Hz, 2,6-F); MS (EI) *m/z* (%) 254.0720 ($[M]^+$, 100) ($C_{14}H_{10}F_4$ requires 254.0719).

The analytical data obtained were in agreement with the literature. [15]

(*Lab book reference number*: GMHP-6-364, GMHP-7-441)

2,3,5,6-tetrafluoromethoxy-4′-(methyl)biphenyl

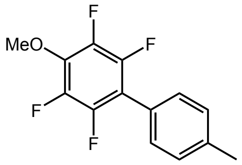

The reaction of 2,3,5,6-tetrafluoroanisole (190 µL, 246 mg, 1.36 mmol, 1.5 equiv.) and 4-iodotoluene **2b** (198 mg, 0.908 mmol) was prepared following the general procedure A. Purification by flash chromatography (SiO_2, R_f =0.16, petroleum-ether 40–60 °C) gave the product (227 mg, 92%) as a white solid; m.p. 43.6–45.0 °C (lit.[15] 49–51 °C); ν_{max}/cm^{-1} (disc, KBr) 2953 and 2925 (CH_3 str.), 1501 (–CH=CH- str.), 1487 (C=C aromatic str.), 1095 (C–F str.), 981 (=C–H in-plane bend), 825 (=C–H oop bend); ^1H NMR (400 MHz, 16 scans, C_6D_6): δ = 7.22 (2 H, d, *J* = 8.0 Hz, 2′,6′-H), 6.99 (2 H, d, *J* = 8.0 Hz, 3′,5′-H), 3.50 (3 H, t, *J* = 1.0 Hz, OCH_3), 2.07 (3 H, s, CH_3); ^{13}C NMR (100 MHz, 1024 scans, $CDCl_3$): δ = 144.5 (dm, *J* = 245.5 Hz, CF), 141.3 (ddt, *J* = 246.5, 16.0, 4.5 Hz, CF), 139.0 (s), 137.4 (tt, *J* = 12.0, 3.5 Hz, 4-C), 130.2 (t, *J* = 2.0 Hz, 2′,6′-C), 129.4 (s, 3′,5′-C), 124.4 (t, *J* = 2.0 Hz), 114.4 (t, *J* = 17.5 Hz), 62.3 (t, *J* = 3.5 Hz, OCH_3), 21.4 (s, CH_3); ^{19}F NMR (376 MHz, 64 scans, C_6D_6): δ = −145.47 (2 F, dd, *J* = 22.5, 9.0 Hz, 2,6-F), −158.45 (2 F, dd, *J* = 22.5, 8.5 Hz, 3,5-F); MS (EI) *m/z* (%) 270.0679 ($[M]^+$, 100) ($C_{14}H_{10}F_4O$ requires 270.0668).

The analytical data obtained were in agreement with the literature[15]

(*Lab book reference number*: GMHP-5-253, GMHP-7-442)

2,3,5,6-tetrafluorochloro-4′-(methyl)biphenyl

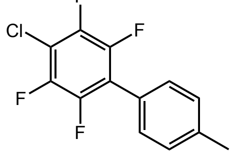

The reaction of 1-chloro-2,3,5,6-tetrafluorobenzene (165 µL, 254 mg, 1.38 mmol, 1.5 equiv.) with 4-iodotoluene **2b** (198 mg, 0.909 mmol, 1 equiv.) was prepared following the general procedure A. Purification by flash chromatography (SiO_2, R_f =0.40, petroleum-ether 40–60 °C) gave the product as a white crystalline solid (237 mg, 95%); m.p. 126.5–128.0 °C; ν_{max}/cm^{-1} (ATR) 3033 (C–H aromatic str.), 2926 (CH_3 str.), 1466 (C=C aromatic str.), 967 (=C–H in-plane bend), 819 (=C–H oop bend); ^1H NMR (400 MHz, 16 scans, C_6D_6): δ = 7.13 (2 H, dt, *J* = 6.5, 1.5 Hz, 2′,6′-H), 6.97 (2 H, d, *J* = 8.0 Hz, 3′,5′-H), 2.06 (3 H, s, CH_3); ^{13}C NMR (100 MHz, 1024 scans, $CDCl_3$): δ = 144.5 (dddd, *J* = 249.0, 16.5, 4.5, 2.5 Hz, CF), 144.3 (dddd, *J* = 248.0, 12.5, 6.0, 5.0 Hz, CF), 139.7 (s), 130.1 (t, *J* = 2.0 Hz, 2′,6′-C), 129.6 (s, 3′,5′-C), 123.8 (t, *J* = 2.0 Hz), 119.7 (t, *J* = 17.0 Hz), 110.9 (tt, *J* = 19.0, 3.0 Hz), 21.5 (s, CH_3); ^{19}F NMR (376 MHz, 64 scans, C_6D_6): δ = −141.89 (2 F, td, *J* = 12.0, 4.0 Hz, 2,6-F), −143.12 to −143.22 (2 F, m, 3,5-F); MS (EI) *m/z* (%) 274. 0172 ($[M]^+$, 100) ($C_{13}H_7ClF_4$ requires 274.01722).

(*Lab book reference number*: GMHP-5-270, GMHP-7-455)

2,3,5,6-tetrafluoro(trifluoromethyl)-4′-(methyl)biphenyl

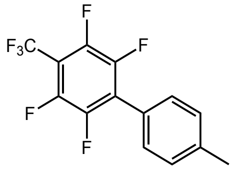

The reaction of 2,3,5,6-tetrafluorobenzotrifluoride (185 µL, 296 mg, 1.36 mmol, 1.5 equiv.) with 4-iodotoluene **2b** (198 mg, 0.908 mmol, 1 equiv.) was prepared following the general procedure A. Purification by flash chromatography (SiO_2, R_f =0.38, petroleum-ether 40–60 °C) gave the major product (259 mg, 93 %) as a white crystalline solid; m.p. 116.5–118.0 °C (lit.[16] 111–114 °C); ν_{max}/cm^{-1} (disc, KBr) 3044 (C–H aromatic str.), 2927 (CH_3 str.), 1477 (C=C aromatic str.), 1339, 1146 (C–F str.), 988 (=C–H in-plane bend), 825 (=C–H oop bend); ^1H NMR (400 MHz, 16 scans, C_6D_6): δ = 7.09 (2 H, d, *J* = 8.1 Hz, 2′,6′-H), 6.95 (2 H, d, *J* = 8.0 Hz, 3′,5′-H), 2.05 (3 H, s, 4-H); ^{13}C NMR (100 MHz, 1024 scans, $CDCl_3$): δ = 144.6 (dm, *J* = 258.5 Hz, CF), 144.3 (ddt, *J* = 248.5, 13.0, 4.5 Hz, CF), 140.4 (s), 130.0 (t, *J* = 2.0 Hz, 2′,6′-C), 129.7 (s, 3′,5′-C), 125.1 (t, *J* = 16.5 Hz), 123.2 (s), 122.5–119.7 (dm, *J* = 275.0 Hz, CF_3), 108.9–107.8 (m), 21.5 (CH_3); ^{19}F NMR (376 MHz, 64 scans, C_6D_6): δ = −55.94 (3 F, t, *J* = 21.5 Hz, CF_3), −141.35 to −141.49 (2 F, m, 3,5-F), −142.17 (2 F, td, *J* = 15.0, 5.5 Hz, 2,6-F); MS (EI) *m/z* (%) 308.0416 ($[M]^+$, 100) ($C_{14}H_7F_7$ requires 308.0436).

The analytical data obtained were in agreement with the literature.[10]

(*Lab book reference number*: GMHP-5-256, GMHP-7-443)

2,3,5,6-tetrafluoro(dimethylamino)-4′-(methyl)biphenyl

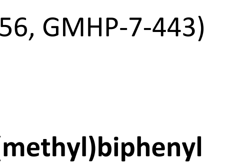

The reaction of 2,3,5,6-tetrafluoro-*N*,*N*-dimethylaniline (200 µL, 266 mg, 1.38 mmol, 1.5 equiv.) and 4-iodotoluene **2b** (197 mg, 0.904 mmol, 1 equiv.) was prepared following the general procedure A. Purification by flash chromatography (SiO_2, R_f =0.07 petroleum-ether 40–60 °C) gave the major

product (232 mg, 91%) as a white solid; m.p. 116.5−118.0 °C (lit.[16] 111–113 °C); ν_{max}/cm^{-1} (ATR) 2923 (CH_3 str.), 1640, 1512 (–CH=CH– str.), 1480 (C=C aromatic str.), 1221 (C–F str.), 1056 (C–H str.), 972 (=C–H in-plane bend), 827 (=C–H oop bend); ^{1}H NMR (400 MHz, 16 scans, C_6D_6): δ = 7.32 (2 H, dt, *J* = 8.0, 1.5 Hz, 2′,6′–H), 7.00 (2 H, dt, *J* = 8.0, 0.5 Hz, 3′,5′-H), 2.59 (6 H, t, *J* = 2.0 Hz, $N(CH_3)_2$), 2.07 (3 H, s, CH_3); ^{13}C NMR (100 MHz, 1024 scans, $CDCl_3$): δ = 144.9 (dddd, *J* = 198.0, 18.0, 9.0, 5.0 Hz), 142.4 (dddd, *J* = 194.0, 15.5, 6.5, 4.0 Hz, CF), 138.6 (s), 130.2 (t, *J* = 2.0 Hz, 2′,6′-C), 129.4 (s, 3′,5′-C), 128.6 (s), 125.1–125.0 (m), 112.9 (t , *J* = 17.5 Hz), 43.5 (t, *J* = 4.0 Hz, $N(CH_3)_2$), 21.5 (s, CH_3); ^{19}F NMR (376 MHz, 64 scans, C_6D_6): δ = −146.27 to −146.35 (2 F, m, 2,6-F), −151.96 to −152.05 (2 F, m, 3,5-F); MS (EI) *m/z* (%) 283.0986 ($[M]^+$, 100) ($C_{15}H_{13}F_4N$ requires 283.0984).

The analytical data obtained were in agreement with the literature.[10]

(*Lab book reference number*: GMHP-6-365, GMHP-7-469)

Pentafluorodeuterobenzene (1-d)

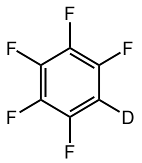

Magnesium-turnings (5.50 g, 0.23 mol, 1.4 equiv.), and 160 cm^3 of dry diethylether were sequentially added to a three-neck round-bottom flask equipped with condenser under atmosphere of nitrogen. The reaction was initiated by quick addition of 6 cm^3 dry pentafluorobromobenzene (20 cm^3, 40 g, 0.16 mol, 1 equiv.) followed by slow, dropwise addition. After two hours of stirring, D_2O (14 cm^3, 15 g, 0.77 mol, 4.8 equiv.) was added to the brown reaction mixture and stirred for 30 min. The yellow organic phase was dried over Mg_2SO_4, solvent removed on the rotary evaporator and purified by distillation under continuous flow of nitrogen at 100 °C. The product **1-d** was obtained as colourless oil (7 cm^3, 11 g, 40%); b.p. 84 °C; density 1.532 g cm^3; ν_{max}/cm^{-1} (CaF_2 cell, DCM) 1642 and 1516 (–CH=CH– str.), 1069 (C–F str.), 1018 and 1006 (=C–H in-plane bend); ^{2}H NMR (76 MHz, 16 scans, C_6H_6): δ = 5.83 (s); ^{13}C NMR (126 MHz, 6729 scans, $CDCl_3$): δ = 146.4 (dm, *J* = 249.5 Hz, CF), 142.0 (dtt, *J* = 254.0, 13.5, 5.0 Hz, CF), 137.8 (dm, *J* = 250.5 Hz, CF), 101.1–100.3 (m, CD); ^{19}F NMR (471 MHz, 16 scans, $CDCl_3$): δ = −136.44 (2 F, dd, *J* = 21.5, 8.5 Hz, 2,6-F), −151.15 (1 F, t, *J* = 20.0 Hz, 4-F), −159.55 (2 F, td, *J* = 21.0, 8.5 Hz, 3,5-F).

A literature procedure was followed for the synthesis of this compound.[17] The analytical data obtained were in agreement with the literature.

(*Lab book reference number*: GMHP-5-254, GMHP- 6-341)

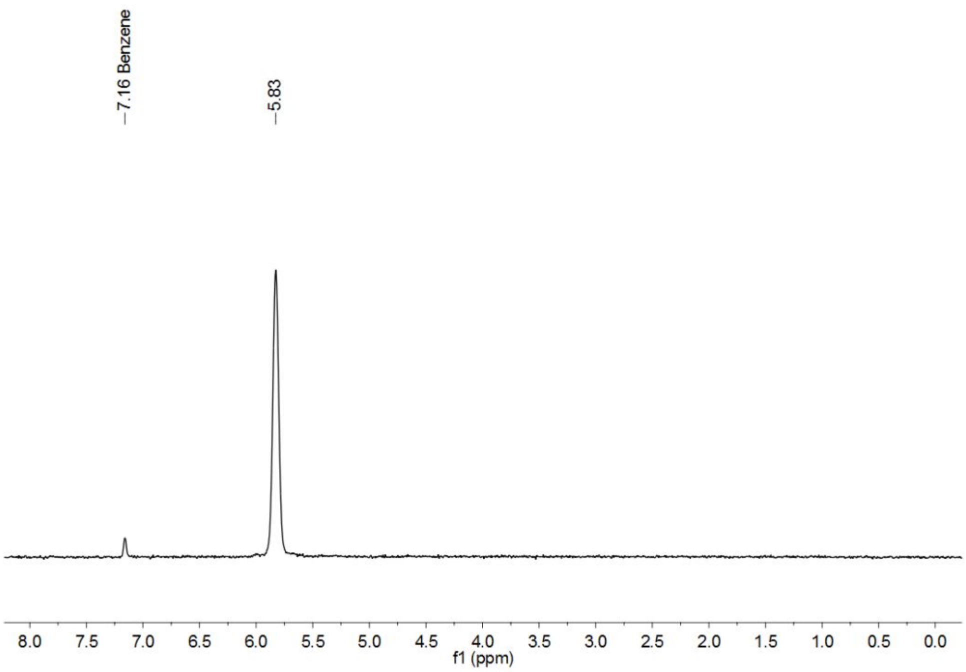

Figure S1. ^{2}H NMR spectrum of deuteropentafluorobenzene **1-*d*** in benzene.

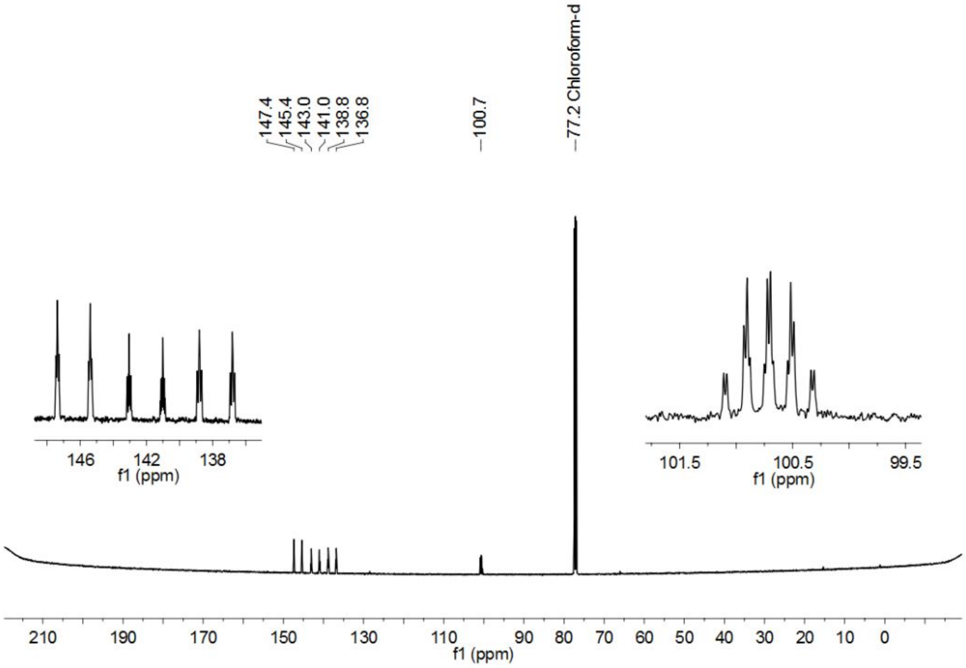

Figure S2. ^{13}C NMR spectrum of deuteropentafluorobenzene **1-d** in chloroform-d

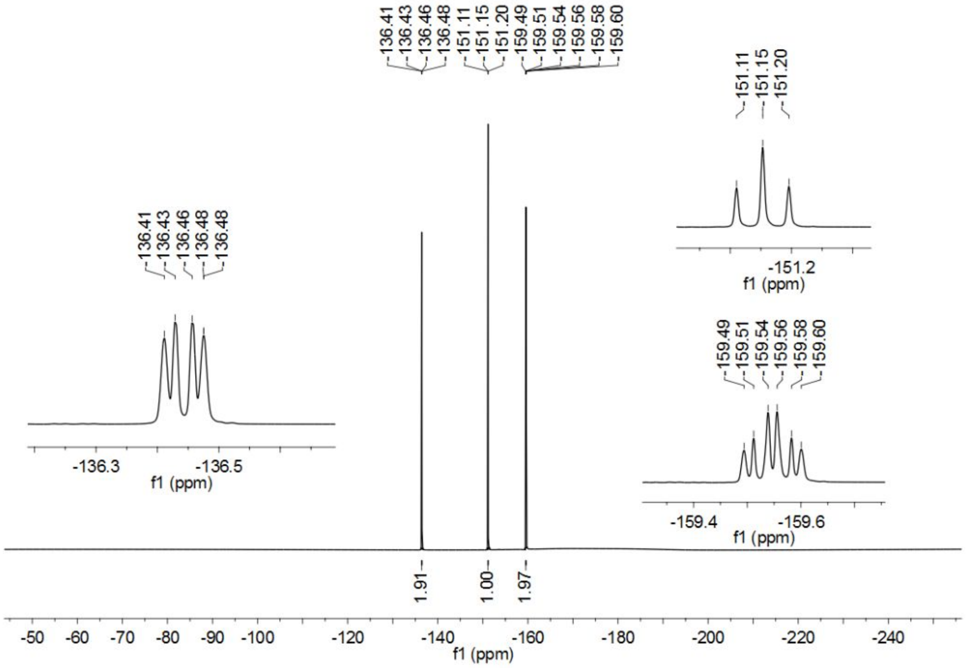

Figure S3 ^{19}F NMR spectrum of deuteropentafluorobenzene **1-d** in chloroform-d.

2,3,5,6-tetrafluoro-*N*,*N*-dimethylaniline (121)

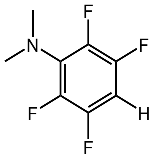

A mixture of pentafluorobenzene **1** (2.6 cm^3, 3.9 g, 0.023 mol) and 33% w/w ethanolic dimethylamine (5.0 cm^3) were heated in a pressure tube at 75 °C for 20 hours. Once the crude mixture was cooled to room temperature, 200 cm^3 Et_2O and 40 cm^3 deionized water were added for liquid-liquid extraction. The aqueous phase was back-extracted with Et_2O (2 × 200 cm^3). The combined organic phase was dried over Na_2SO_4, filtered and solvent removed under vacuum to give the product **121** as clear yellow oil (1.9 g, 43%). The product contained 10% of 2,3,4,5-tetrafluoro-*N*,*N*-dimethylaniline; ν_{max}/cm^{-1} (ATR) 2981 (C–H aromatic str.), 2889 (CH_3 str.), 1641 (–CH=CH– str.), 1499 (C=C aromatic str.), 1055 (C–F str.), 933 (=C–H in-plane bend), 813 (=C–H oop bend); ^{1}H NMR (400 MHz, 32 scans, $CDCl_3$): δ =6.63 (1 H, tt, *J* = 10.0, 7.0 Hz, 1-CH), 2.95 (6H, t, *J* = 2.0 Hz, $N(CH_3)_2$); ^{13}C NMR (100 MHz, 1024 scans, $CDCl_3$): δ = 146.8 (dm, *J* = 245.5 Hz, CF), 142.3 (dm, *J* = 244.0 Hz, CF), 132.2 (tt, *J* = 10.5, 2.5 Hz), 97.9 (tm, *J* = 23.0 Hz), 43.3–43.2 (m, Me); ^{19}F NMR (376 MHz, 128 scans, $CDCl_3$): δ = −141.06 to –141.17 (2 F, m, CF), −151.59 to −151.68 (2 F, m, CF).

A literature procedure was followed for the synthesis of this compound.[18] The analytical data obtained were in agreement with the literature.

(*Lab book reference number*: GMHP-6-361, GMHP-7-467)

Synthesis of Ag (C_6F_5)(PPh_3)$_n$

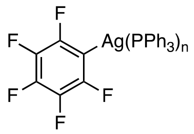

In an argon glovebox, AgC_6F_5 (100 mg, 0.36 mmol, 1 equiv.) and PPh_3 (189 mg, 0.72 mmol, 2 equiv.) were weighed out and transferred to a foil wrapped Schlenk tube. A suba seal was fitted to the mouth of the Schlenk tube, the side-arm was closed, and then the Schlenk tube was removed from the glovebox. The Schlenk tube was connect to a Schlenk line, and the side arm was evacuated and refilled three time. Under a flow of nitrogen, CH_3CN (2 mL) was added and the Schlenk tube was submerged in a pre-heated oil bath (80°C). The reaction mixture was heated at 80 °C for 2 h before being filter canula transferred to another hot Schlenk tube (80 °C) while the reaction mixture was still hot. The reaction mixture was slowly cooled to room temperature to yield the product as dark brown crystals (42.2 mg, 15 %). The crystals were filtered and dried under reduced pressure. The filtrate wa transferred into another Schlenk tube and slowly cooled to – 18 °C, to yield more product as brown crystals (167.8 mg, 58%). The total yield of product is 210 mg, 73 %.

NMR: $^{31}P\{^1H\}$ NMR (202 MHz, Tol:CD_2Cl_2 (20:80), 25 °C) δ = 4.9 (s)

$^{19}F\{^1H\}$ NMR (471 MHz, Tol:CD_2Cl_2 (20:80), 25 °C) δ –107.5 (2 *F*, m, 2,6-F), –159.6 (1 *F*, br s, 4-F), –161.9 (2 *F*, m, 3,5-F);

$^{31}P\{^1H\}$ NMR (202 MHz, Tol:CD_2Cl_2 (20:80), –100 °C): spectra complex, discussed in chapter 2.

$^{19}F\{^1H\}$ NMR (471 MHz, Tol:CD_2Cl_2 (20:80), –100 °C): –107.0 (2 *F*, m, 2,6-F), –159.6 (1 *F*, t, *J* = 23.5 Hz, 4-F), –161.0 (2 *F*, m, 3,5-F)

MS (LIFDI) m/z (%) 262.09055 ($[PPh_3]^+$, 100) (calcd for $C_{18}H_{15}P$ 262.0911, diff 0.00055), 631.08782 ($[Ag(PPh_3)_2]^+$, 98.5) (calcd for $C_{36}H_{30}AgP$ 631.0874, diff 0.00042), 798.07929 ($[M]^+$, 0.68) (calcd for $C_{42}H_{30}AgF_5P_2$ 798.07883, diff 0.00046), 893.18357 ($[Ag(PPh_3)_3]^+$, 5.46) (calcd for $C_{59}H_{95}AgP_3$ 893.1785, diff 0.00507), 906.98600 ($[Ag_2C_6F_5(PPh_3)_2]^+$, 6.34) (calcd for $C_{42}H_{30}Ag_2F_5P_2$ 906.9841, diff 0.0019).

(*Lab book reference number:* GA-4-268)

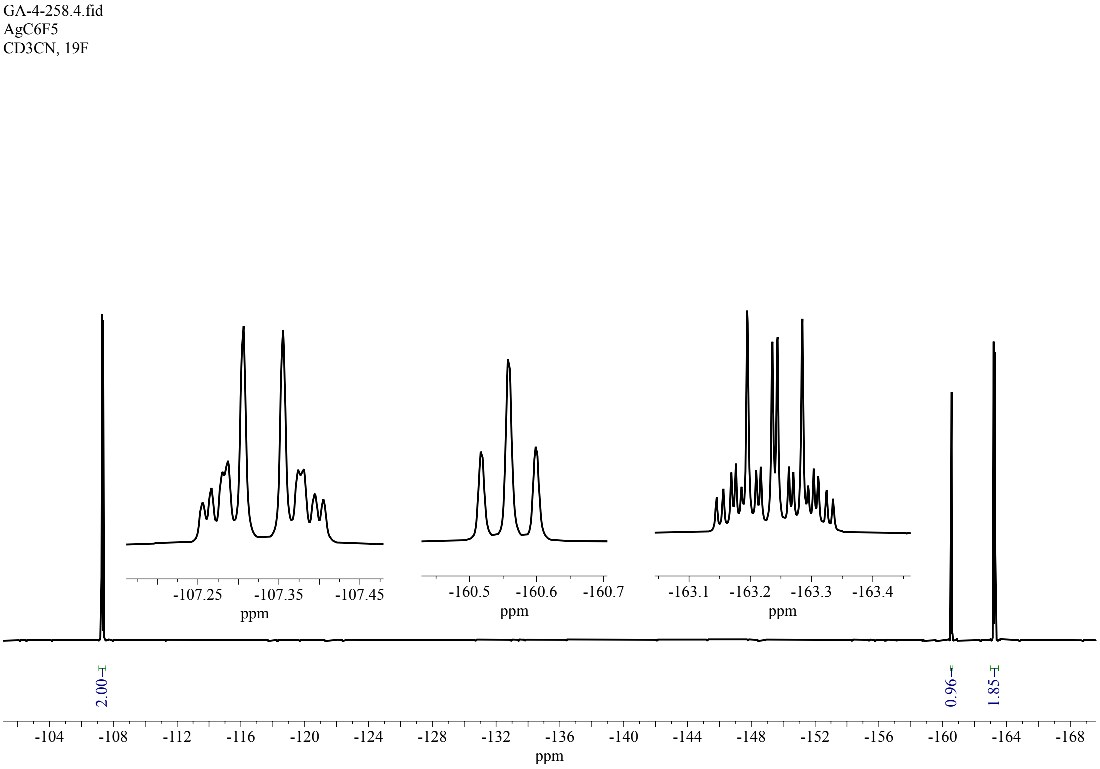

Figure S4: The $^{19}F\{^{1}H\}$ NMR (470.6 MHz, CD_3CN, 25 °C) spectrum of AgC_6F_5 shown for comparison to $Ag(C_6F_5)(PPh_3)_n$

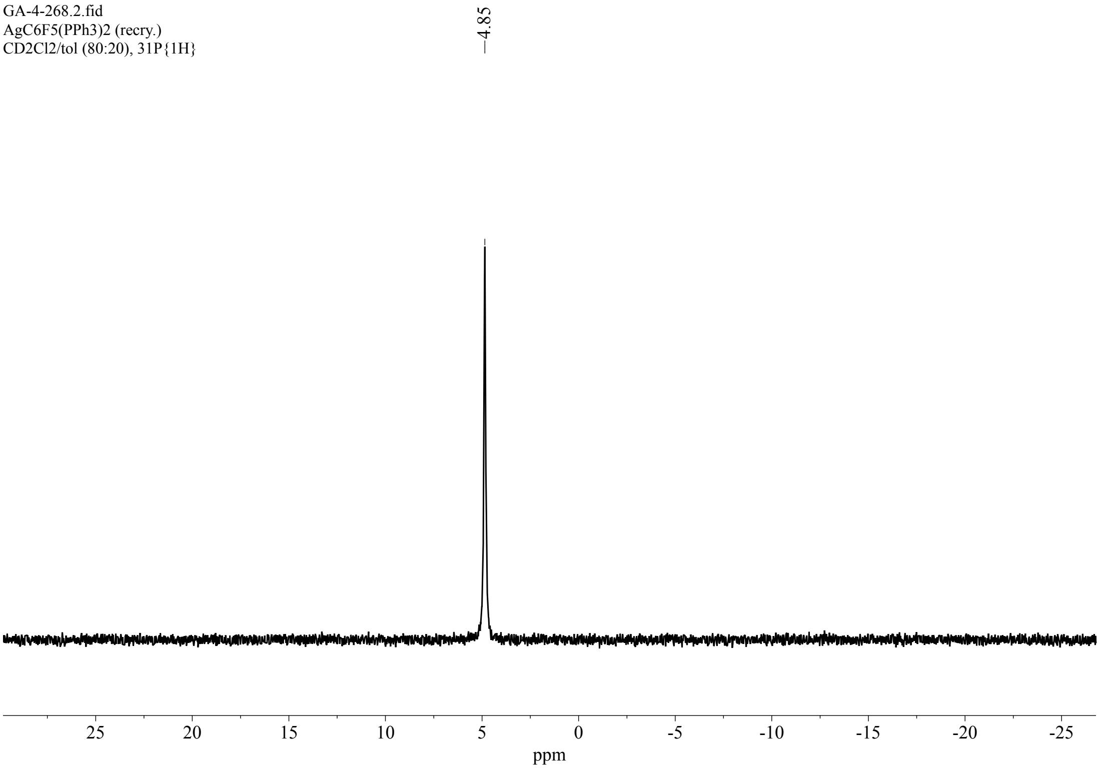

Figure S5 $^{31}P\{^{1}H\}$ NMR (202 MHz, tol-d_8:dichloromethane-d_2 (20:80), 25 °C) spectrum of $Ag(PPh_3)_n(C_6F_5)$

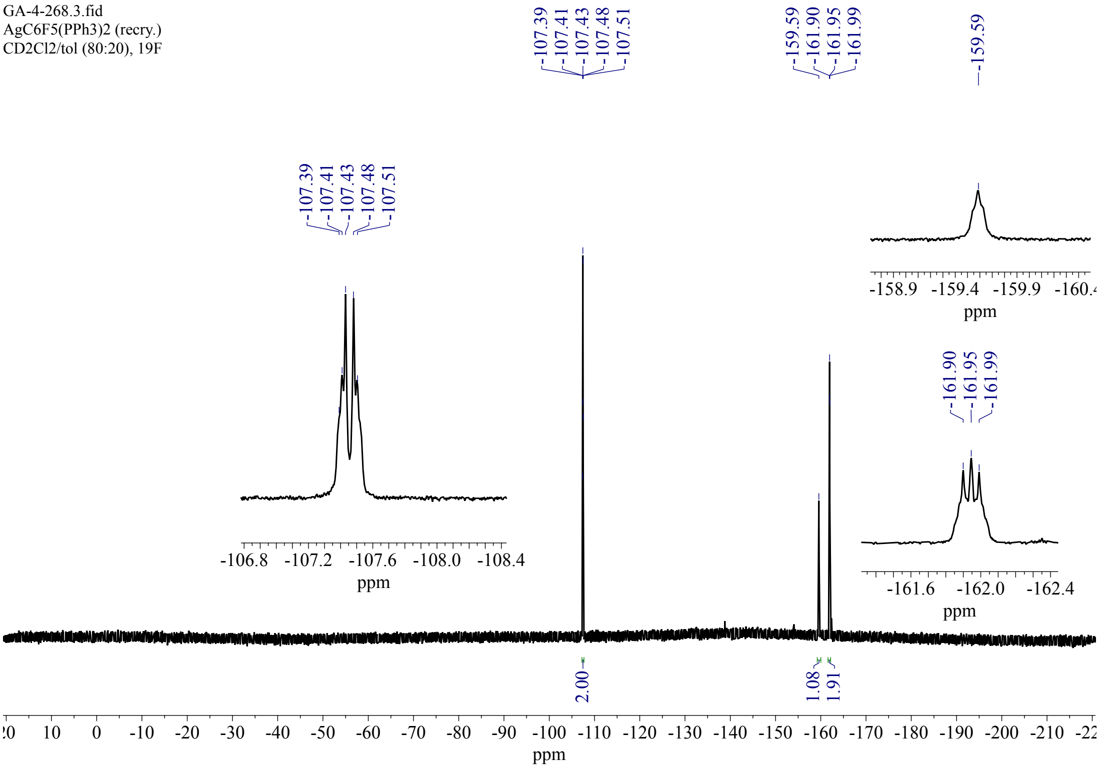

Figure S6: $^{19}F\{^{1}H\}$ NMR (470 MHz, tol-d_8:dichloromethane-d_2 (20:80), 25 °C) spectrum of $Ag(PPh_3)_n(C_6F_5)$

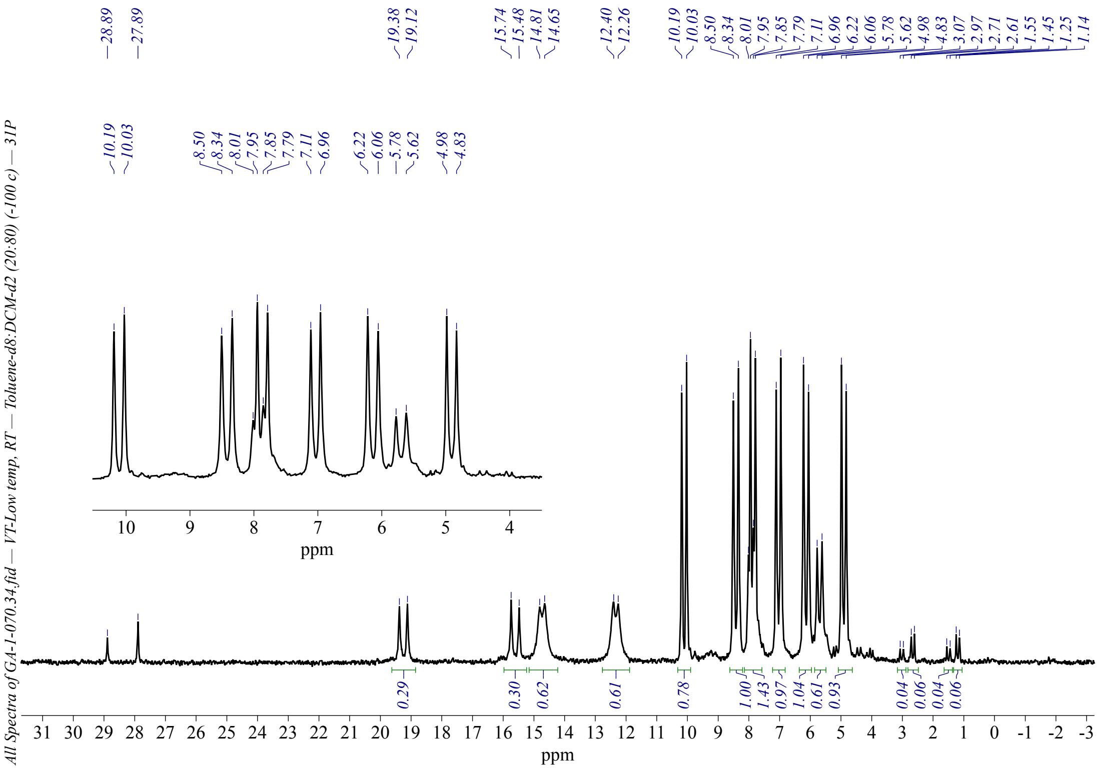

Figure S7: $^{31}P\{^{1}H\}$ NMR (202 MHz, tol-d_8:dichloromethane-d_2 (20:80), –100 °C) spectrum of $Ag(PPh_3)_n(C_6F_5)$

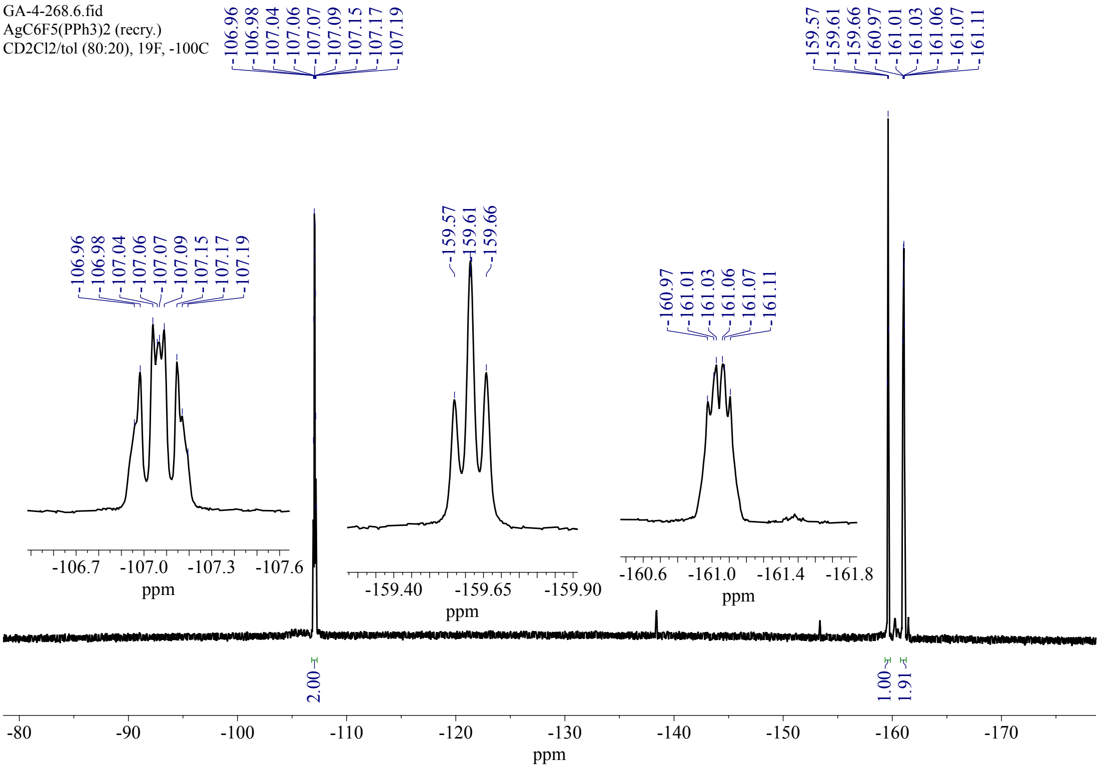

Figure S8:$^{19}F\{^1H\}$ NMR (470 MHz, tol-d_8:dichloromethane-d_2 (20:80), −100 °C) spectrum of $Ag(C_6F_5)(PPh_3)_n$

4. Studies of conversions for coupling of 1 and 2a

Standard reaction conditions for results for Tables S1, S2, S3 and S5:

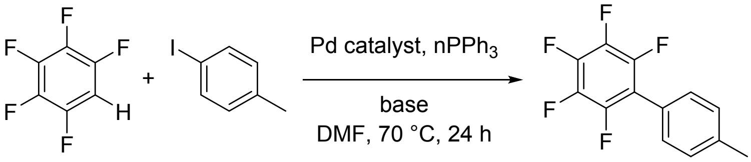

Table S1. Conversions of **2a** and yields of isolated **3a** using different bases.[a]

Entry	Base	Equiv base	Catalyst	%Conversion[b] (%Yield)[c]
1	Ag_2CO_3	0.75	$Pd(OAc)_2$	quant. (84)
2	K_2CO_3	0.75	$Pd(OAc)_2$	40 (26)
3	Cs_2CO_3	0.75	$Pd(OAc)_2$	70 (31)
4	AgOAc	1.5	$Pd(OAc)_2$	31 (23)
5	AgOAc,	1.5	$Pd(PPh_3)_4$	38 (24)
6	Ag_2O	0.75	$Pd(OAc)_2$	quant. (72)
7	Ag_2O,	0.75	$Pd(PPh_3)_4$, no PPh_3	(75)
8	$[Me_4N]OAc$	1.5	$Pd(OAc)_2$	quant. (82)
9	$[Me_4N]Br$	1.5	$Pd(OAc)_2$	0
10	$[Me_4N]Cl$	1.5	$Pd(OAc)_2$	0
11	$[^nBu_4N]OAc$	1.5	$Pd(OAc)_2$	48 (21)

[a] Catalyst concentration 5 mol% with 10 mol% PPh_3 unless stated otherwise.

[b] Standard reaction time 24 h. Determined from integration of methyl ^1H NMR peaks of **2a** and **3a**.

[c] After purification by flash chromatography.

Table S2. Effect of solvent on conversions and yields of isolated **3a.**[a]

Entry	Variable	%Conversion[b] (%Yield)[c]
1	DMF	quant. (84)
2	DMAc	quant. (79)
3	NMP	(67)
4	Toluene	0
5	1,4-dioxane	28 (15)
6	ethylene carbonate	(50)
7	propylene carbonate	87 (51)

[a] Conditions 5 mol% $Pd(OAc)_2$, 10 mol% PPh_3, 0.75 equiv Ag_2CO_3, 70 °C, 24 h

[b] Determined from integration of methyl ^1H NMR peaks of the reagent **2a** and the product **3a**.

[c] After purification by flash chromatography.

Standard conditions for results of changing phosphine (Table S4)

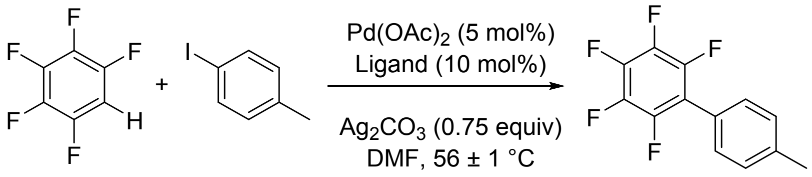

1 (180 mM) **2a (18 mM)** **3a**

Table S3. Effect of phosphine on conversion.

Entry	Ligand	%Conversion [a]
1	PPh_3	quant.
2	$P(4\text{-}FC_6H_4)_3$	quant.
3	$P(3,5\text{-}(CF_3)_2C_6H_3)_3$	quant.
4	P(2-Furyl)$_3$	quant.
5	$P(2\text{-}MeOC_6H_4)_3$	89
6	$P(4\text{-}MeOC_6H_4)_3$	69
7	$P(2\text{-}MeC_6H_4)_3$	24
8	PCy_3	11

[a] Determined by integration of the methyl-peaks of the starting material and the product by ^{1}H NMR spectroscopic analysis of the reaction mixture.

Table S4. Yields of isolated **3a** using Pd-NPs on PVP support as pre-catalyst

**Entry**	**Condition**				**%Yield** [a]
	**Pd-NP Catalyst**	**Wt% Pd-NP**	**Ligand**	**T / °C**	
1	29K-PVP	2	-	70	Trace
2	29K-PVP	8	-	70	Trace
3	10K-PVP	2	PPh_3	70	55
4	29K-PVP	2	PPh_3	70	67
5	29K-PVP	8	PPh_3	70	59
6	55K-PVP	2	PPh_3	70	48
7	29K-PVP	8	-	100	21
8	55K-PVP	2	-	100	10
9	$Pd(OAc)_2$	5 mol%	-	100	25

[a] Yield of the isolated product **3a** after purification by flash chromatography.

Table S5. Product ratios (P^X/P^F) and substituent constants (σ^+) for X in 1-X-2,3,5,6-tetrafluorobenzenes.

**X**	**σ^+ for X**	**P^X/P^F** [a]
NMe_2	−1.70	0.33
OMe	−0.78	0.56
F	−0.07	1.00
Cl	+0.11	1.17
CF_3	+0.61	1.38

[a] Calculated from integration of ^{19}F NMR signals of the crude reaction mixture in chloroform-d with 512 scans and 30 s relaxation delay.

5. Speciation of silver complexes

5.1 General Procedure for reactions of Ag[I] salts in acetonitrile

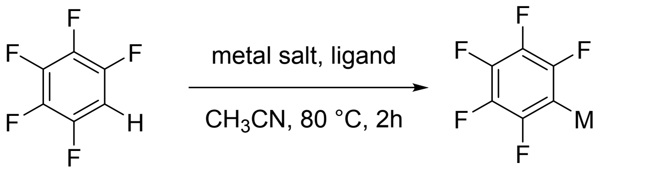

1 (10 equiv) **M = Ag, Ag(PPh$_3$)$_n$**

In a light-protected Schlenk tube, the desired Ag[I] salt, and additives (if required) were added and the Schlenk tube was evacuated and refilled three times with nitrogen. Under an atmosphere of nitrogen, dry acetonitrile (1 mL) was added and the Schlenk tube was submerged in a pre-heated oil bath (80 °C). Using a gas-tight microsyringe (250 µL), pentafluorobenzene (168 mg, 1 mmol, 10 equiv., 111 µL) was added and the reaction was stirred at 80 °C for 2 h. Then, 0.6 mL of the reaction mixture was transferred to a J-Young NMR tube, under as atmosphere of nitrogen and the mixture was immediately analysed by ^{1}H and ^{19}F{^{1}H} NMR spectroscopy. If phosphine was added, the reaction mixture was also analysed by ^{31}P{^{1}H} NMR spectroscopy. Results presented in **Table S1**.

GA-1-049 (*Entry 1*): AgOAc (17 mg, 0.1 mmol, 1 equiv.) and no phosphine was added.
GA-1-046 (*Entry 2*): T Ag_2CO_3 (28 mg, 0.1 mmol, 1 equiv.) and no phosphine was added.
GA-1-043 (*Entry 3*): AgOAc (17 mg, 0.1 mmol, 1 equiv.) and Ag_2CO_3 (28 mg, 0.1 mmol, 1 equiv.) and no phosphine was added.
GA-1-052 (*Entry 4*): AgOAc (17 mg, 0.1 mmol, 1 equiv.), followed by PPh_3 (52 mg, 0.2 mmol, 2 equiv.).
GA-1-044 (*Entry 5*): AgOAc (17 mg, 0.1 mmol, 1 equiv.) and Ag_2CO_3 (28 mg, 0.1 mmol, 1 equiv.), followed by PPh_3 (52 mg, 0.2 mmol, 2 equiv.).
GA-1-047 (*Entry 6*): Ag_2CO_3 (28 mg, 0.1 mmol, 1 equiv.), followed by PPh_3 (52 mg, 0.2 mmol, 2 equiv.).
GA-1-083 (*Entry 7*): AgOAc (17 mg, 0.1 mmol, 1 equiv.), followed by Cs_2CO_3 (33 mg, 0.1 mmol, 1 equiv.).
GA-1-084 (*Entry 8*): s AgOAc (33 mg, 0.2 mmol, 1 equiv.), followed by Cs_2CO_3 (163 mg, 0.5 mmol, 2.5 equiv.). 5 equiv of C_6F_5H used with respect to AgOAc.

Table S6 Screening reaction of different silver salts and additives with **1** for conversion to Ag(C_6F_5) or Ag(C_6F_5)(PPh_3)$_n$

Entry	Metal salt	Ligand	Conversion[a] (%)
1	AgOAc (1 equiv.)	-	0
2	Ag_2CO_3 (1 equiv.)	-	Trace
3	Ag_2CO_3 (1 equiv.) + AgOAc (1 equiv.)	-	Trace
4	AgOAc (1 equiv.)	PPh_3 (2 equiv.)	0
5	Ag_2CO_3 (1 equiv.) + AgOAc (1 equiv.)	PPh_3 (2 equiv.)	25
6	Ag_2CO_3 (1 equiv.)	PPh_3 (2 equiv.)	100
7	AgOAc (1 equiv.) + Cs_2CO_3 (1 equiv.)	-	83
8[b]	AgOAc (1 equiv.) + Cs_2CO_3 (2.5 equiv.)		45

[a] The reaction was performed in CH_3CN at 80 °C for 2 h. The % conversion was calculated by integrating the *ortho*-F resonance of the starting material C_6F_5H (δ –139) with respect to $Ag(C_6F_5)$ (δ –107.4). For reactions with PPh_3, the *ortho*-F resonance of $Ag(C_6F_5)(PPh_3)_n$ (δ –106.4) is used.
[b] 5 equivalents of pentafluorobenzene were used in this reaction

5.2 The reaction of Ag_2CO_3 and PPh_3 with C_6F_5H in DMF at 60 °C

In a light-protected Schlenk tube, Ag_2CO_3 (82 mg, 0.3 mmol, 1 equiv.) and PPh_3 (157 mg, 0.6 mmol, 2 equiv.) were added and the Schlenk tube was evacuated and refilled three times with nitrogen. Dry DMF (3 mL) and C_6F_5H were added and the Schlenk tube was submerged in a pre-heated oil bath (60 °C) and allowed to react for 24 h. The progress of the reaction was monitored using reaction aliquots. The reaction aliquots were prepared by transferring 0.5 mL of the reaction mixture into a J-Young NMR tube using a syringe, under an atmosphere of nitrogen. The reaction aliquots were collected at 2 h, 4 h, 24 h, and 48 h analysed using ^1H, $^{31}P\{^1H\}$ and $^{19}F\{^1H\}$ NMR spectroscopy. Results presented in Table S7.
GA-1-050 (*Entry 1*): 10 equiv C_6F_5H with respect to the Ag_2CO_3. The C_6F_5H (504 mg, 3 mmol, 10 equiv. 330 µL) was added with a 500 µL gas-tight syringe.
GA-1-053 (*Entry 2*): 1 equiv C_6F_5H with respect to the Ag_2CO_3. The C_6F_5H (50 mg, 0.3 mmol, 1 equiv. 35 µL) was added with a 50 µL gas-tight syringe.

Table S7 The reaction of Ag_2CO_3 and PPh_3 with **1** in DMF at 60 °C to form $Ag(C_6F_5)(PPh_3)_n$

Entry	Equivalents of C_6F_5H	Time (h)	Conversion[a] (%)
1	10	2	80
		4	90
		24	100
2	1	2	77
		4	83
		24	90
		48	90

[a] The % conversion was calculated by integrating the *ortho*-F resonance of the starting material C_6F_5H (δ –139.2) with respect to the AgC_6F_5 complex (δ –106.2) in DMF.

5.3 Reactions with Phosphine-coordinated Silver(I) complexes

To a light-protected Schlenk tube, the desired silver complex (0.1 mmol, 1 equiv.) and any required additives were added and the Schlenk tube as evacuated and refilled three times with nitrogen. Against a flow of nitrogen, dry DMF (3 mL) and C_6F_5H **1** (168 mg, 1.0 mmol, 10 equiv. 110 µL) were added mixture was allowed to react for 24 h at the desired temperature. The reaction was monitored using a reaction aliquot which was prepared by transferring 0.5 mL of the reaction mixture into a J Young tube after 24 h. The reaction mixture was monitored by $^{31}P\{^1H\}$ and $^{19}F\{^1H\}$ NMR spectroscopy. Results presented in Table S8.

GA-6-505 (*Entry 1*): The reaction of $Ag(PPh_3)_2(\kappa^2\text{-}OAc)$ (69 mg, 0.1 mmol, 1 equiv.) with 10 equiv **1** in DMF heated to 60 °C for 24 h. No formation of an AgC_6F_5 containing product was detected in the $^{19}F\{^1H\}$ NMR spectrum.

GA-6-504 (*Entry 2*): The reaction of $Ag(PPh_3)_2(\kappa^2\text{-}HCO_3)$ (69 mg, 0.1 mmol, 1 equiv.) with 10 equiv **1** in DMF heated to 60 °C for 24 h. No formation of an AgC_6F_5 containing product.
GA-6-501 (*Entry 3*): The reaction of $Ag(PPh_3)_2(\kappa^2\text{-}HCO_3)$ (69 mg, 0.1 mmol, 1 equiv.) and Cs_2CO_3 (32 mg, 0.1 mmol, 1 equiv.) with 10 equiv of **1** in DMF heated to 60 °C for 24 h. The formation of an AgC_6F_5 containing product was detected, but a % conversion was not calculated due to the ill-defined nature of the product in solution.
GA-6-499 (*Entry 4*): The reaction between $[Ag(PPh_3)_2]_2CO_3$•2 H_2O (132 mg, 0.1 mmol, 1 equiv.) with 10 equiv of **1** in DMF heated to 60 °C in DMF. After the addition of **1**, the reaction mixture was submerged in a pre-heated oil bath (60 °C) for 24 h. The formation of an AgC_6F_5 containing product was detected, but a % conversion was not calculated due to the ill-defined nature of reactant and product in solution.
GA-6-500 (*Entry 5*): The reaction between $[Ag(PPh_3)_2]_2CO_3$•2 H_2O (132 mg, 0.1 mmol, 1 equiv.) with 10 equiv **1** in DMF was stirred at room temperature for 24 h. The formation of a AgC_6F_5 containing product was detected, but a % conversion was not calculated due to the ill-defined nature of both reactant and product in solution. However, from the relative integration with respect to C_6F_5H, the reaction at room temperature is more sluggish than at 60 °C.

Reaction between $Ag(PPh_3)_2(\kappa^2\text{-}OAc)$ with 1 equiv C_6F_5H (1) in DMF
In a light-protected Schlenk tube, $Ag(PPh_3)_2(\kappa^2\text{-}OAc)$ (138.3 mg, 0.2 mmol, 1 equiv.) was added and the Schlenk tube was evacuated and refilled three times with nitrogen. Dry DMF (2 mL) was added, and
the reaction mixture was submerged in a pre-heated oil bath (60 °C). C_6F_5H **1** (35 mg, 0.2 mmol, 1 equiv., 25 µL) was added and the mixture was stirred at 60 °C for 24 h. After 24 h, 0.5 mL of the reaction mixture was transferred into a J Young tube under nitrogen and analysed by monitored by $^{31}P\{^1H\}$ and $^{19}F\{^1H\}$ NMR spectroscopy.

Table S8. Low temperature $^{31}P\{^1H\}$ NMR data (–100 °C in toluene/CD_2Cl_2 80:20) of the product from reaction of Ag_2CO_3 with PPh_3 (2 equiv) and C_6F_5H (10 equiv) in CH_3CN at 80 °C

Entry[a]	Peak position of $^{31}P – ^{107}Ag$ peaks		Peak position of $^{31}P – ^{109}Ag$ peaks		Resonance centered at: (δ)	Integration		Coupling constant $^{31}P – ^{107}Ag$ (Hz)	Coupling constant $^{31}P – ^{109}Ag$ (Hz)
	Peak 1	Peak 2	Peak 1	Peak 2		Peak 1	Peak 2		
1	2.61	1.25	2.71	1.14	1.9	0.06	0.06	275	317
2	2.97	1.55	3.07	1.45	2.3	0.04	0.04	287	327
3	6.96	4.98	7.11	4.83	6.0	0.97	0.93	400	461
4	7.85	5.78	8.01	5.62	6.8	1.43	0.61	418	483
5	8.35	6.22	8.50	6.06	7.4	1.00	1.04	430	493
6	10.03	7.95	10.19	7.79	9.0	0.78	1.43	420	485
7	14.65	12.4	14.81	12.26	13.5	0.62	0.61	455	515
8	19.12	15.74	19.38	15.48	17.4	0.29	0.30	683	788

[a]] The coupling constants of **Entry 1**, **2**, **4**, **7** and **8**, can be assigned easily as these doublets do not overlap with each other and the integrations aid in easily distinguishing the peaks. However, with **Entry 3**, **5** and **6**, there is some uncertainty when assigning the peaks as their integration values are very similar and the doublets tend to overlap with each other.

Table S9. Low temperature $^{31}P\{^{1}H\}$ NMR data (–100 °C in toluene/CD_2Cl_2 80:20) of $[Ag(PPh_3)_2]_2CO_3$•2 H_2O

Entry	Peak position of $^{31}P - ^{107}Ag$ (δ)		Peak position of $^{31}P - ^{109}Ag$ (δ)		Resonance Centered at (δ)	Integration		Coupling constant $^{1}J_{^{31}P - ^{107}Ag}$ (Hz)	Coupling Constant $^{1}J_{^{31}P - ^{109}Ag}$ (Hz)
	Peak 1	Peak 2	Peak 1	Peak 2		Peak 1	Peak 2		
1	0.09	–1.76	0.22	–1.90	–0.8	0.07	0.06	374	428
2	2.63	1.27	2.74	1.17	2.0	1.00	1.00	275	317
3	2.98	1.57	3.09	1.46	2.3	0.15	0.14	285	329
4[a]	6.88	4.85	7.02	4.70	5.9	1.69	1.65	~410	~469
5[a]	7.02	4.98	7.14	4.85	6.0			~412	~463
6	8.66	6.48	8.83	6.31	7.6	1.42	1.42	440	509
7	8.93	6.72	9.1	6.54	7.8			446	517
8	19.16	15.81	19.39	15.54	17.5	0.12	0.12	677	778

[a] These peaks overlap and appear as pairs of pseudo-triplets. The analysis assumes that they are overlapping pairs of doublets but there us some ambiguity in the assignments

Table S10. Low temperature $^{31}P\{^{1}H\}$ NMR data (–100 °C in toluene/CD_2Cl_2 80:20) of the Ag-P resonances of from reaction of $Ag(C_6F_5)$ with 2 PPh_3 in CH_3CN at 80 °C

Entry	Peak position of $^{31}P - ^{107}Ag$ (δ)		Peak position of $^{31}P - ^{109}Ag$ (δ)		Resonance Centered at (δ)	Integration		Coupling constant $^{1}J_{^{31}P - ^{107}Ag}$ (Hz)	Coupling Constant $^{1}J_{^{31}P - ^{109}Ag}$ (Hz)
	Peak 1	Peak 2	Peak 1	Peak 2		Peak 1	Peak 2		
1	1.98	0.64	2.08	0.53	1.3	0.41	0.36	271	313
2	2.32	0.91	2.43	0.80	1.6	0.27	0.25	285	329
3	4.40	3.28	4.44	3.19	3.8		1.47	226	253
4	4.48	3.06	4.54	2.96	3.8	2.89	1.38	287	319
5	6.04	4.94	6.13	4.81	5.5	1.00	1.04	222	267

Table S11. Comparison of the closest matches for the peaks of $Ag_2(PPh_3)_2(C_6F_5)$(**μ2**-HCO_3) and $Ag(C_6F_5)(PPh_3)_n$ and $[Ag(PPh_3)_2]_2CO_3$•2 H_2O

Entry	Source Complex	Chemical shift (δ)	Coupling constant (Hz) $^{1}J_{^{31}P - ^{107}Ag}$	Coupling constant (Hz) $^{1}J_{^{31}P - ^{109}Ag}$
1	$Ag_2CO_3/PPh_3/C_6F_5H$	1.9	275	317
	$Ag(C_6F_5)(PPh_3)_n$	1.3	271	313
	$[Ag(PPh_3)_2]_2CO_3$•2 H_2O	2.0	275	317
2	$Ag_2(PPh_3)_2(C_6F_5)$(μ2-HCO_3)	2.3	287	327
	$Ag(C_6F_5)(PPh_3)_n$	1.6	285	329
	$[Ag(PPh_3)_2]_2CO_3$•2 H_2O	2.3	285	329
3	$Ag_2CO_3/PPh_3/C_6F_5H$	17.4	683	788
	$[Ag(PPh_3)_2]_2CO_3$•2 H_2O	17.5	677	778

5.4 Reactions with Catalytic Amounts of Ag^{I} salt

Direct arylation reaction with Cs_2CO_3 as the base and catalytic amounts of Ag^{I} salts and complexes

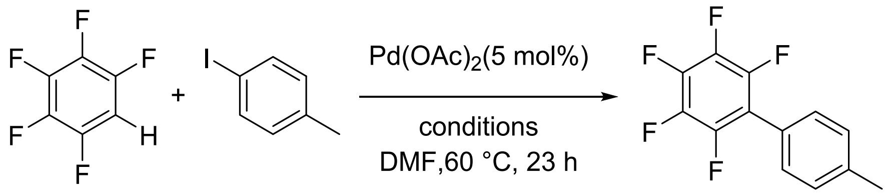

1 (1 equiv) **2a (1.5 equiv)** **3a**

In a light-protected Schlenk tube $Pd(OAc)_2$ (10 mg, 0.045 mmol, 5 mol%), PPh_3 (as required), Ag additive, 1,3,5-trimethoxybenzene (44 mg, 0.26 mmol, 0.28 equiv., internal standard) and Cs_2CO_3 (221 mg, 0.68 mmol, 0.75 equiv.) was added and the Schlenk tube was evacuated and refilled three times with nitrogen. Against a strong flow of nitrogen, 4-iodotoluene (196 mg, 0.9 mmol, 1 equiv.) was added followed by DMF (2.6 mL) and pentafluorobenzene (227 mg, 1.35 mmol, 1.5 equiv., 150 μ L). The reaction mixture was then left stirring at the required temperature and reaction aliquots were collected to monitor the reaction using ^{1}H NMR spectroscopy. The reaction aliquots were prepared by collecting approximately 0.1 mL of the reaction mixture and filtering it through celite using $CDCl_3$ as the eluent. The sample was locked to $CDCl_3$ and the ^{1}H NMR spectrum of the reaction mixture was collected using a d1 = 1 sec, sw = 16, O1P = 6.175, 32 scans.

GA-6-509 (*Entry 2*): PPh_3 (12 mg, 0.045 mmol, 5 mol%), $[Ag(PPh_3)_2]_2CO_3$•2 H_2O (60 mg, 0.045 mmol, 5 mol%) was added along with the other reagents, and the Schlenk tube was evacuated are refilled three times with nitrogen. After the addition of all the remaining reagents, the reaction mixture was submerged in a preheated oil bath (60 °C) and stirred for 23 h. The following reactions were performed similarly.

GA-6-510 (*Entry 3*): PPh_3 (12 mg, 0.045 mmol, 5 mol%), $[Ag(PPh_3)_2(\kappa^2\text{-}HCO_3)]_2$ (62 mg, 0.045 mmol, 5 mol%)
GA-6-511 (*Entry 1*): Only PPh_3 (23.6 mg, 0.09 mmol, 10 mol%) with no added Ag additive.
GA-6-514 (*Entry 6*): PPh_3 (12 mg, 0.045 mmol, 5 mol%), $[Ag(PPh_3)_2]_2CO_3$•2 H_2O (30 mg, 0.023 mmol, 2.5 mol%)
GA-6-515 (*Entry 5*): PPh_3 (12 mg, 0.045 mmol, 5 mol%), $[Ag(PPh_3)_2(\kappa^2\text{-}HCO_3)]_2$ (31 mg, 0.023 mmol, 2.5 mol%)
GA-6-516 (*Entry 4*): PPh_3 (23 mg, 0.09 mmol, 10 mol%), Ag_2CO_3 (12 mg, 0.045 mmol, 5 mol%)
GA-6-521 (*Entry 7*): PPh_3 (23 mg, 0.09 mmol, 10 mol%), Ag_2CO_3 (6.2 mg, 0.023 mmol, 2.5 mol%)
GA-6-521 (*Entry 8*): PPh_3 (23 mg, 0.09 mmol, 10 mol%), Ag_2CO_3 (2.5 mg, 0.009 mmol, 1 mol%)
GA-7-533 (*Entry 9*): PPh_3 (23.6 mg, 0.09 mmol, 10 mol%), AgC_6F_5 (25 mg, 0.09 mmol, 10 mol%)

6. Analysis of Catalytic Intermediates by in situ HR-MAS NMR Spectroscopy

6.1 Direct Arylation of 4-Iodotoluene 2a with 1 catalysed by $Pd(OAc)_2$

The rotor for the solid-state NMR spectroscopy was charged with $Pd(OAc)_2$ (1.1 mg, 4.9×10^{-6} mol, 50 mol%), PPh_3 (2.6 mg, 9.9×10^{-6} mol, 100 mol%), Ag_2CO_3 (2.4 mg, 8.7×10^{-6} mol, 1 equiv.), 4-iodotoluene **2a** (2.1 mg, 9.6×10^{-6} mol, 1 equiv.), DMF-d_7 (50 µL) and **1** (1.5 µL, 2.3 mg, 1.4×10^{-5} mol, 1.6 equiv.) respectively. The mixture was heated at 328.5 K inside the spectrometer and the progress was monitored by ^{1}H and ^{31}P NMR spectroscopic analysis at *ca.* 17 min intervals until reaction completion without decreasing the temperature.

(*Lab book reference number*: GMHP-6-386)

A second sample with the same mixture, initially at 285.0 K, was heated at 328.5 K inside the spectrometer until *ca.* 20% conversion of the starting material was achieved. At this point the reaction was cooled to 308.5 K, 288.5 K, 268.5 K and 248.5 K in sequential order. The mixture was studied by 1D (^{1}H, ^{19}F and ^{31}P) and 2D (^{1}H-^{1}H COSY and ^{1}H-^{31}P HMQC) NMR at each temperature.

(*Lab book reference number*: GMHP-6-417)

6.2 Direct Arylation of Iodobenzene 2b with 1 catalysed by $Pd(OAc)_2$

The rotor for the solid-state NMR spectroscopy was charged with $Pd(OAc)_2$ (1.1 mg, 4.9×10^{-6} mol, 50 mol%), PPh_3 (2.5 mg, 9.5×10^{-6} mol, 100 mol%), Ag_2CO_3 (2.4 mg, 8.7×10^{-6} mol, 1 equiv.), iodobenzene **2b** (1 µL, 1.8 mg, 8.9×10^{-6} mol, 1 equiv.), DMF-d_7 (50 µL) and **1** (1.5 µL, 2.3 mg, 1.4×10^{-5} mol, 1.6 equiv.) respectively. The mixture, initially at 285.0 K, was heated at 328.5 K inside the spectrometer until *ca.* 20% conversion of the starting material was achieved. At this point the reaction was cooled to 308.5 K, 288.5 K, 268.5 K and 248.5 K in sequential order. The mixture was studied by 1D (^{1}H, ^{19}F and ^{31}P) and 2D (^{1}H-^{1}H COSY and ^{1}H-^{31}P HMQC) NMR at each temperature.

(*Lab book reference number*: GMHP-7-449)

6.3 Direct Arylation of Iodobenzene 2b with 1 Catalysed by $[Pd(Ph)(\mu\text{-}OAc)(PPh_3)]_2$

The rotor for the solid-state NMR spectroscopy was charged with $[Pd(Ph)(\mu\text{-}OAc)(PPh_3)]_2$ **84** (2.4 mg, 2.4×10^{-6} mol, 50 mol% Pd atom), Ag_2CO_3 (2.4 mg, 8.7×10^{-6} mol, 1 equiv.), iodobenzene **81** (1 µL, 1.8 mg, 8.9×10^{-6} mol, 1 equiv.), DMF-d_7 (50 µL) and **1** (1.5 µL, 2.3 mg, 1.4×10^{-5} mol, 1.6 equiv.) respectively. The mixture, initially at 285.0 K, was heated at 328.5 K inside the spectrometer until *ca.* 20% conversion of the starting material was achieved. At this point the reaction was cooled to 308.5 K, 288.5 K, 268.5 K and 248.5 K in sequential order. The mixture was studied by 1D (^{1}H, ^{19}F and ^{31}P) and 2D (^{1}H-^{1}H COSY and ^{1}H-^{31}P HMQC) NMR at each temperature.

(*Lab book reference number*: GMHP-7-461)

Reaction catalysed by $[Pd(Ph)(\mu\text{-}OAc)(PPh_3)]_2$ with added phosphine ligand

1. $[Pd(Ph)(\mu\text{-}OAc)(PPh_3)]_2$ (2.4 mg, 2.4×10^{-6} mol, 50 mol% Pd atom), PPh_3 (0.6 mg, 2.3×10^{-6} mol, 25 mol%) (*Lab book reference number*: GMHP-7-466)
2. $[Pd(Ph)(\mu\text{-}OAc)(PPh_3)]_2$ (2.4 mg, 2.4×10^{-6} mol, 50 mol% Pd atom), PPh_3 (1.7 mg, 6.6×10^{-6} mol, 75 mol%) (*Lab book reference number*: GMHP-7-471)
3. $[Pd(Ph)(\mu\text{-}OAc)(PPh_3)]_2$ (2.4 mg, 2.4×10^{-6} mol, 50 mol% Pd atom), $O{=}PPh_3$ (1.6 mg, 5.7×10^{-6} mol, 65 mol%) (*Lab book reference number*: GMHP-7-470)

6.4 Characterisation of Pd intermediates by HR-MAS NMR and by LIFDI mass spectometry

The rotor for the solid-state NMR spectroscopy was charged with pre-synthesised Pd complexes (listed below) and DMF-d_7 (50 µL). The samples were analysed at 285.0 K, 328.5 K, 308.5 K, 288.5 K, 268.5 K and 248.5 K in sequential order.

1. $[Pd(Ph)(\mu\text{-}OAc)(PPh_3)]_2$ (*Lab book reference number*: GMHP-7-445-P)
2. $[Pd(Ph)(\mu\text{-}OH)(PPh_3)]_2$ (*Lab book reference number*: GMHP-5-311-P)
3. $[Pd(Ph)(\mu\text{-}I)(PPh_3)]_2$ (*Lab book reference number*: GMHP-5-312-P)
4. $Pd(Ph)(\kappa^1\text{-}OAc)(PPh_3)_2$ (*Lab book reference number*: GMHP-7-446-P)
5. $Pd(Ph)(I)(PPh_3)_2$ (*Lab book reference number*: GMHP-7-444-P)

Table S12. ^{1}**H and** ^{31}**P NMR characterization of Pd species (following heating to 56 °C and cooling to ambient temperature), measured in DMF-d$_7$ at –24 °C**

Entry	Complex	Nucleus	
		^{1}**H** / δ (J/Hz)	^{31}**P** / δ
**1**[a]	Pd(Ph)(κ^1-OAc)(PPh_3)$_2$ **Pd1-OAc**	7.50–7.37 (30 H, m, PPh_3), 6.55 (1 H, t, J = 7.3, 4-CH), 6.49 (2 H, d, J = 7.5 Hz, Ph), 6.28 (2 H, t, J = 7.4, Ph), 0.81 (3 H, s, CH_3)	20.6 (s)
**2**	[Pd(Ph)(μ-OAc)(PPh_3)]$_2$ **Pd2-OAc**	7.46 (8 H, t, J = 7.0, PPh_3), 7.28–7.18 (23 H, m, PPh_3+Ph), 6.89 (2 H, t, J = 7.1, 4-CH), 6.74 (4 H, t = 7.1, Ph), 1.13 (6 H, s, CH_3)	29.3 (s)
**3**[a]	Pd(Ph)(I)(PPh_3)$_2$ **Pd1-I**	7.57–7.34 (30 H, m, PPh_3), 6.62 (2 H, d, J = 7.6, Ph), 6.32 (1 H, t, J = 7.0, 4-CH), 6.21 (1 H, t, J = 7.3, Ph)	33.2 (s), 22.9 (s)
**4**[a, b]	[Pd(Ph)(μ-I)(PPh_3)]$_2$ **Pd2-I**	7.46–7.38 (30 H, m, PPh_3), 6.91–6.87 (2 H, m, Ph), 6.62–6.53 (4 H, m, Ph), 5.91–5.90 (2 H, m , Ph)	23.9 (s)
**5**[a]	[Pd(Ph)(μ-OH)(PPh_3)]$_2$ **Pd2-OH**	7.53–7.38 (30 H, m, PPh_3), 7.01–6.94 (4 H, m, Ph), 6.65–6.60 (6 H, m, Ph), −0.66 to −0.67 (0.6 H, m, *cis*-OH), −1.88 (1.5 H, d, J = 2.96 Hz, *trans*-OH), −3.17 to −3.19 (0.6 H, m, *cis*-OH)	33.2 (s), 32.7 (s)
**6**	product **3b**	7.77 (2H, d, J = 7.0, 2,6-CH), 7.43 (1H, t, J = 7.5, 4-CH), 7.22 (2H, t, J = 7.5, 3,5-CH)	N/A
**7**	pentafluorobenzene **1**	7.91 (m, CH)	N/A
**8**	iodobenzene **2b**	7.64–7.55 (m, Ph)	N/A

6. [a] Decomposition observed after heating to 56 °C with formation of $OPPh_3$. [b] Complex was poorly soluble in DMF-d$_7$.

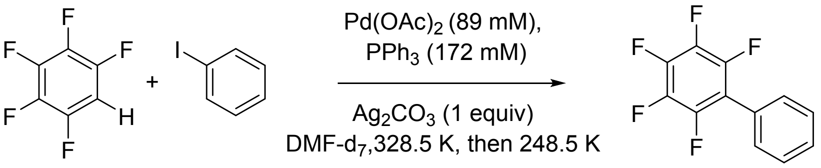

1 (244 mM) **2b** (162 mM) **3b**

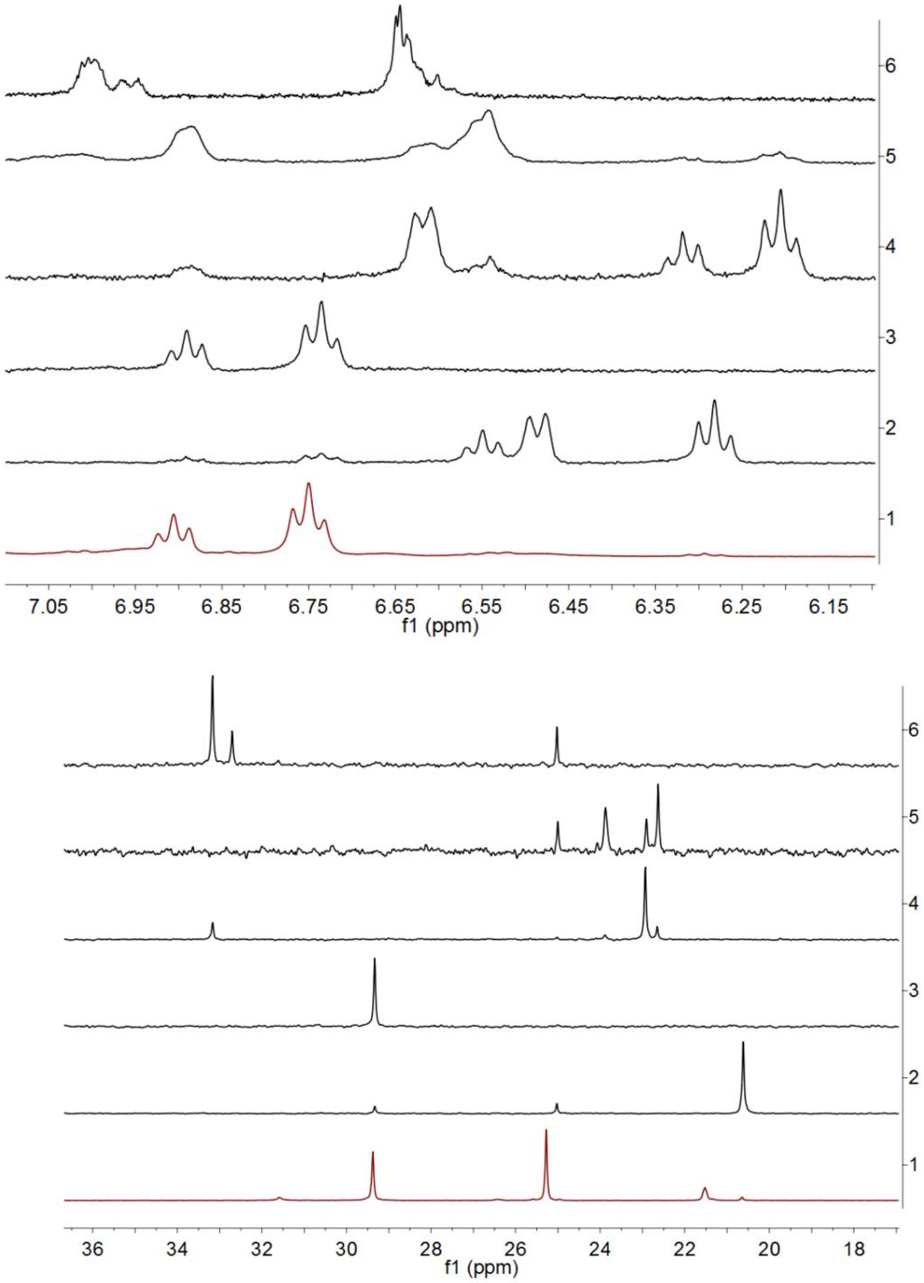

Figure S9. Above: stacked ^{1}H, Below: corresponding ^{31}P{^{1}H} NMR spectra at 248.5 K of (1) the reaction shown in equation above, (2) Pd(Ph)(κ^1-OAc)(PPh$_3$)$_2$ in DMF-d$_7$, (3) [Pd(Ph)(μ-OAc)(PPh$_3$)]$_2$ in DMF-d$_7$, (4) Pd(Ph)(I)(PPh$_3$)$_2$ in DMF-d$_7$, (5) [Pd(Ph)(μ-I)(PPh$_3$)]$_2$ in DMF-d$_7$ and (6) [Pd(Ph)(μ-OH)(PPh$_3$)]$_2$ in DMF-d$_7$.

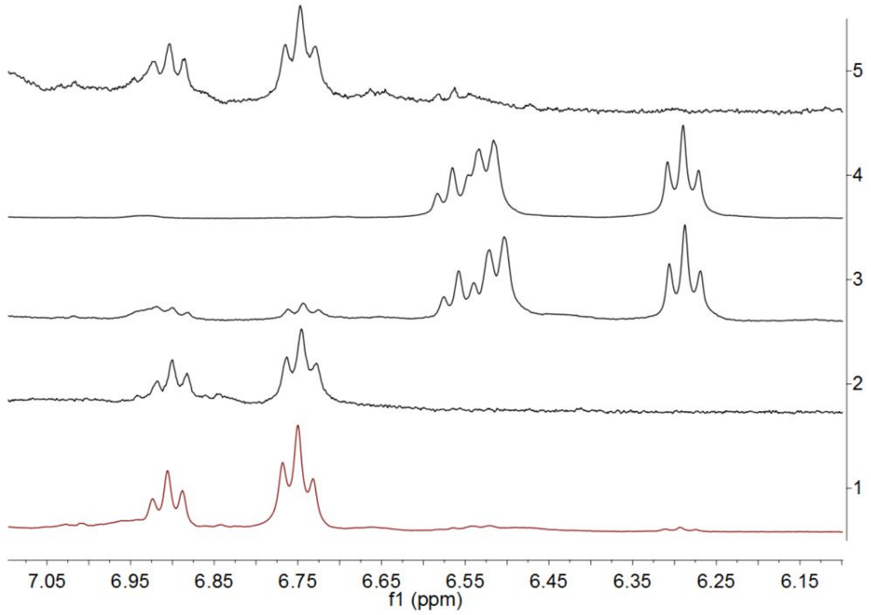

Figure S10. Stacked ^{1}H NMR spectra of the reaction mixtures at 248.5 K in DMF-d_7. Reaction catalysed by (1) 50 mol% $Pd(OAc)_2$ + 100 mol% PPh_3, (2) 25 mol% $[Pd(Ph)(\mu\text{-}OAc)(PPh_3)]_2$, (3) 25 mol% $[Pd(Ph)(\mu\text{-}OAc)(PPh_3)]_2$ + 25 mol% PPh_3, (4) 25 mol% $[Pd(Ph)(\mu\text{-}OAc)(PPh_3)]_2$ + 75 mol% PPh_3 and (5) 25 mol% $[Pd(Ph)(\mu\text{-}OAc)(PPh_3)]_2$ + 65 mol% O=PPh_3.

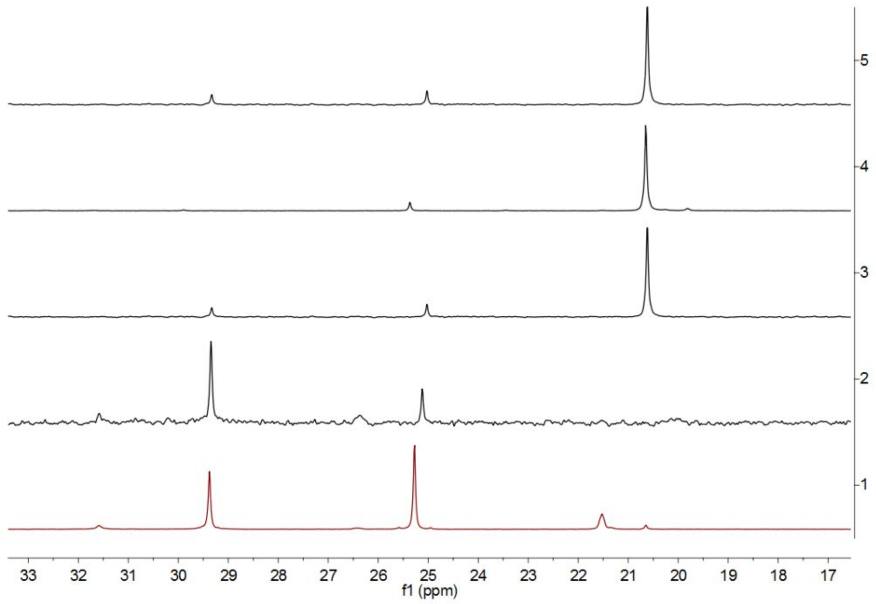

Figure S11. Stacked $^{31}P\{^{1}H\}$ NMR spectra of the reaction mixtures at 248.5 K in DMF-d_7. Reactions catalyzed by (1) 50 mol% $Pd(OAc)_2$ + 100 mol% PPh_3, (2) 25 mol% $[Pd(Ph)(\mu\text{-}OAc)(PPh_3)]_2$, (3) 25 mol% $[Pd(Ph)(\mu\text{-}OAc)(PPh_3)]_2$ + 25 mol% PPh_3 and (4) 25 mol% $[Pd(Ph)(\mu\text{-}OAc)(PPh_3)]_2$ + 75 mol% PPh_3. (5) $Pd(Ph)(\kappa^1\text{-}OAc)(PPh_3)_2$.

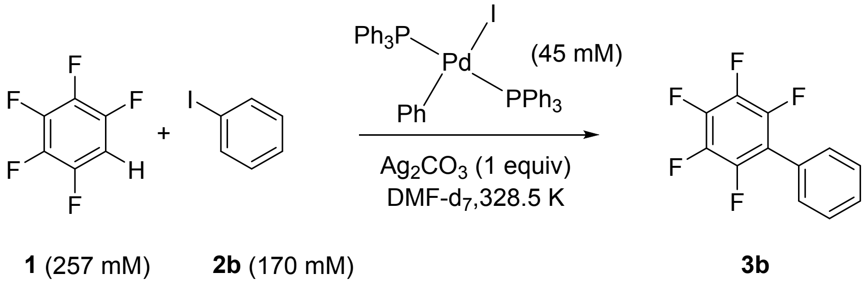

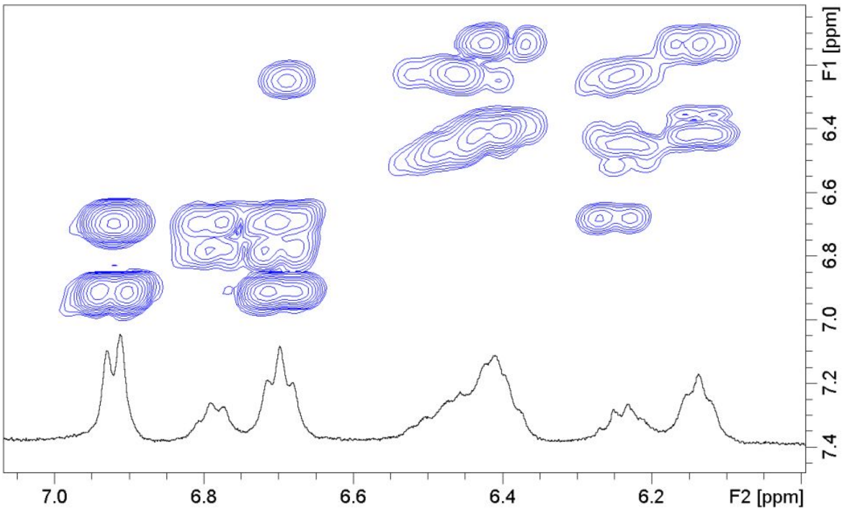

Figure S12. 2D ^1H-^1H COSY NMR spectrum of the reaction shown in equation above at 248.5 K.

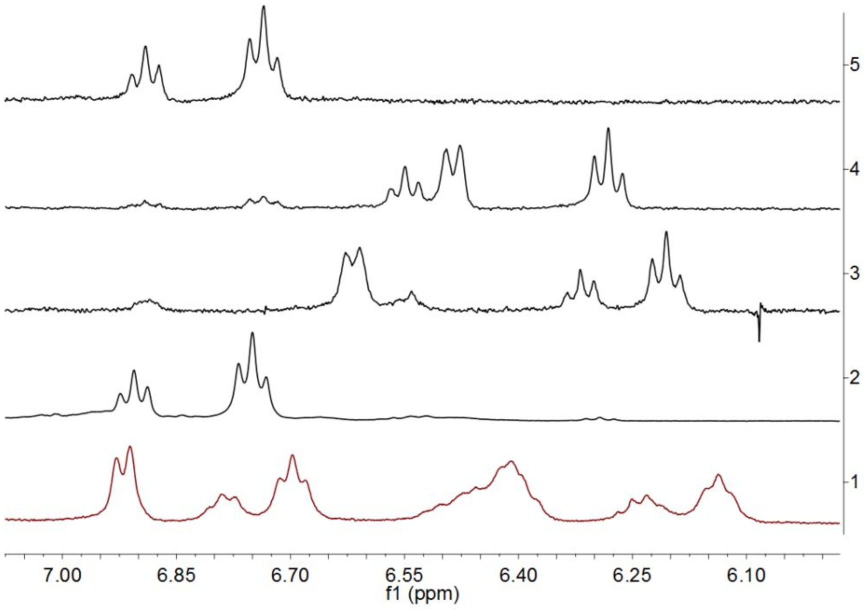

Figure S13. Stacked ^1H NMR spectra in DMF-d7 at 248.5 K. (1) Reaction catalysed by $Pd(Ph)(I)(PPh_3)_2$, (2) reaction catalysed by $Pd(OAc)_2$ and $2PPh_3$, (3) $Pd(Ph)(I)(PPh_3)_2$, (4) $Pd(Ph)(\kappa^1\text{-}OAc)(PPh_3)_2$ and (5) $[Pd(Ph)(\mu\text{-}OAc)(PPh_3)]_2$.

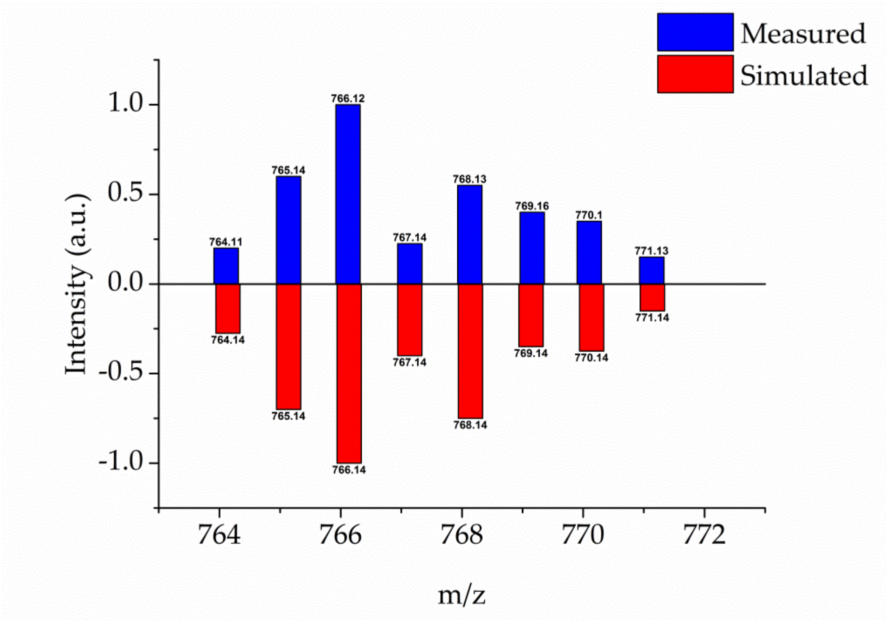

Figure S14. LIFDI-MS spectrum of Pd(Ph)(κ^1-OAc)(PPh_3)$_2$ Pd1-OAc ($PdC_{44}H_{38}O_2P_2$ requires 766.14).

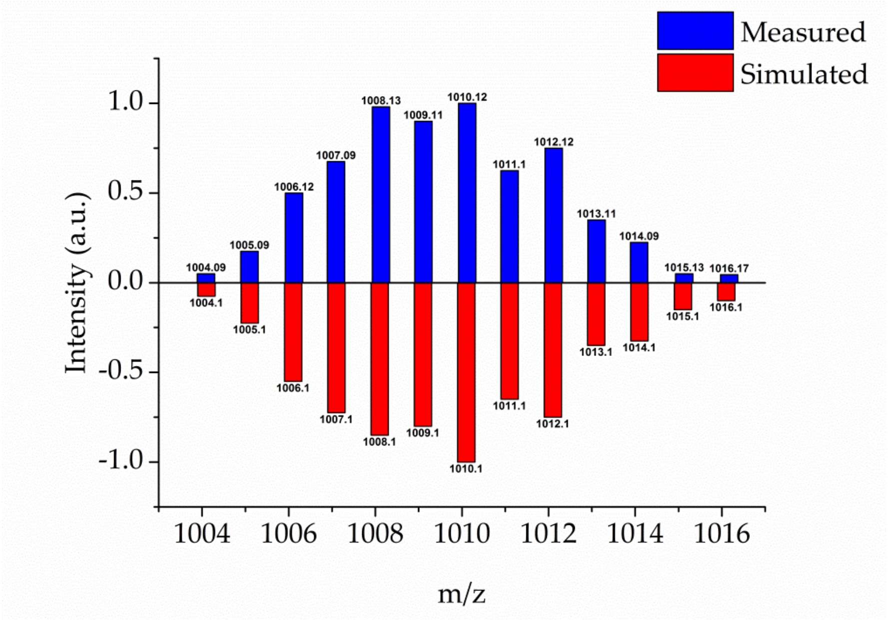

Figure S15. LIFDI-MS spectrum of [Pd(Ph)(μ-OAc)(PPh_3)]$_2$ Pd2-OAc ($Pd_2C_{52}H_{46}O_4P_2$ requires 1010.10**).**

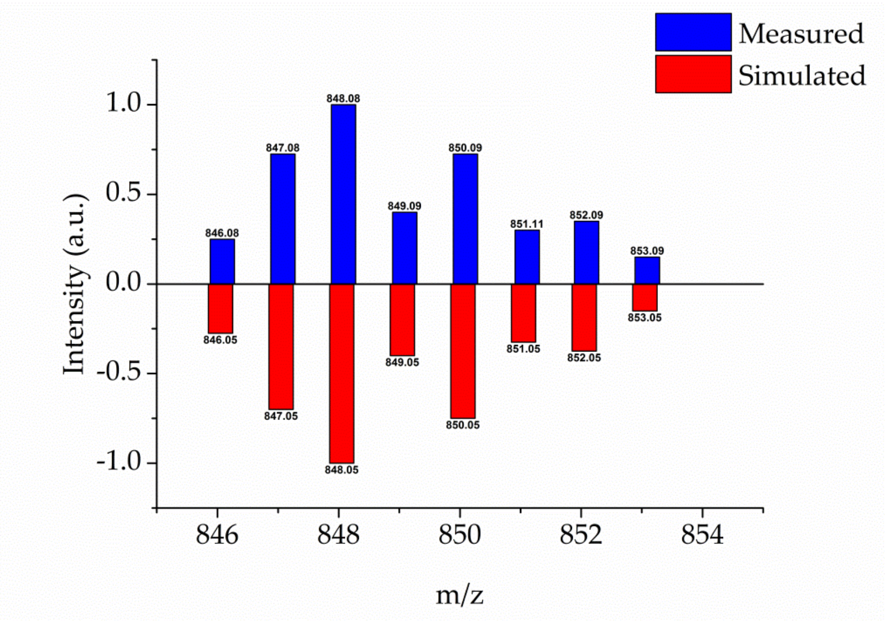

Figure S16. LIFDI-MS spectrum of Pd(4-tolyl)(I)$(PPh_3)_2$ ($PdC_{43}H_{37}IP_2$ requires 848.05).

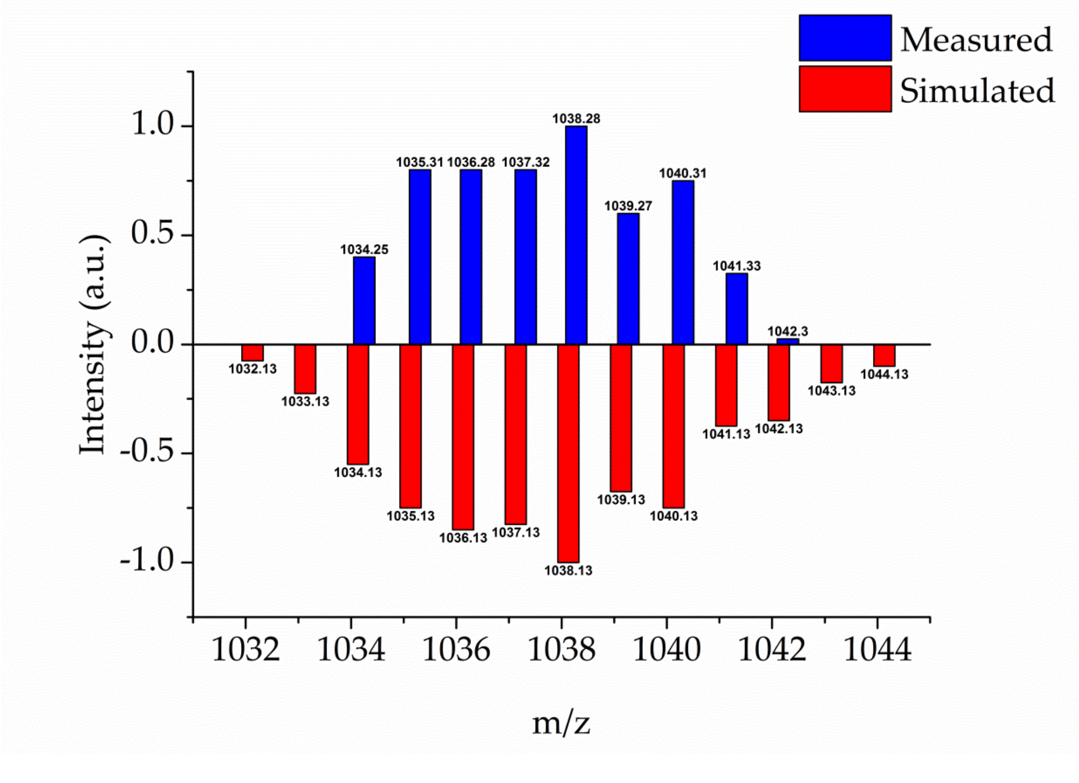

Figure S17. LIFDI-MS spectrum of [Pd(4-tolyl)(μ-OAc)$(PPh_3)]_2$ ($Pd_2C_{54}H_{50}O_4P_2$ requires 1038.13).

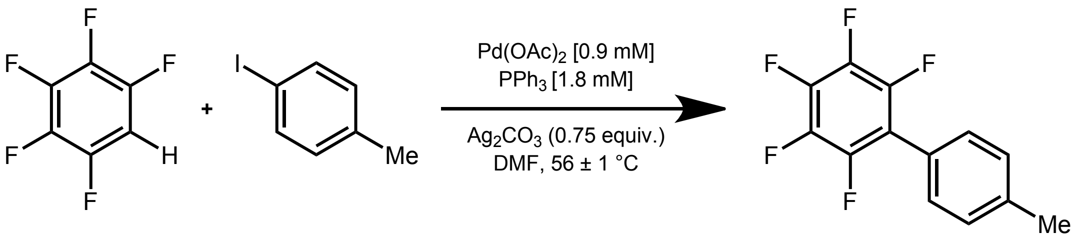

56 [180 mM] **57** [18 mM] **58**

Scheme S1. Direct arylation reaction condition selected for study by LIFDI-MS.

Table S13. Aliquot collection time and species identified by LIFDI-MS of reaction shown in Scheme S1

Entry	Collection Timing	Observed *m/z*	Assignment
**1**	T = 19 ± 1 °C<br>before heating	339.05<br>353.05<br>367.08<br>381.11	$[PPh_4]^+$<br>$[P(4\text{-tolyl})Ph_3]^+$<br>$[P(4\text{-tolyl})_2Ph_2]^+$<br>$[P(4\text{-tolyl})_3Ph]^+$
**2**	T = 56 ± 1 °C<br>t = 0<br>before addition of<br>**1**	339.05<br>353.07<br>367.09<br>381.11<br>518.03<br>689.03<br>885.95<br>1038.11	$[PPh_4]^+$<br>$[P(4\text{-tolyl})Ph_3]^+$<br>$[P(4\text{-tolyl})_2Ph_2]^+$<br>$[P(4\text{-tolyl})_3Ph]^+$<br>$Pd(4\text{-tolyl})(OAc)(PPh_3)$<br>$Pd(OAc)(PPh_3)_2$<br>$Pd(4\text{-tolyl})(\kappa^1\text{-OAc})(PPh_3)–$<br>$Pd(PPh_3)$<br>$[Pd(4\text{-tolyl})(\mu\text{-OAc})(PPh_3)]_2$
**3**	T = 56 ± 1 °C<br>t = 6 min<br>5% conversion of<br>**2a**	353.12<br>518.05<br>886.94<br>1038.16	$[P(4\text{-tolyl})Ph_3]^+$<br>$Pd(4\text{-tolyl})(OAc)(PPh_3)$<br>$Pd(4\text{-tolyl})(\kappa^1\text{-OAc})(PPh_3)–$<br>$Pd(PPh_3)$<br>$[Pd(4\text{-tolyl})(\mu\text{-OAc})(PPh_3)]_2$
**4**	T = 56 ± 1 °C<br>t = 47 min<br>60% conversion of<br>**2a**	277.98<br>353.02<br>517.96<br>887.88	$P(4\text{-tolyl})Ph_2$<br>$[P(4\text{-tolyl})Ph_3]^+$<br>$Pd(4\text{-tolyl})(OAc)(PPh_3)$<br>$Pd(4\text{-tolyl})(\kappa^1\text{-OAc})(PPh_3)–$<br>$Pd(PPh_3)$
**5**	T = 56 ± 1 °C<br>t = 86 min<br>quant. conversion<br>of 4-iodotoluene<br>**57**	277.98<br>353.04<br>593.90<br>856.02	$P(4\text{-tolyl})Ph_2$<br>$[P(4\text{-tolyl})Ph_3]^+$<br>$Pd(C_6F_5)(OAc)(PPh_3)$<br>$Pd(C_6F_5)(\kappa^1\text{-OAc})(PPh_3)_2$
**6**	T = 56 ± 1 °C<br>t = 126 min<br>quant. conversion<br>of **2a**	277.98<br>353.01<br>593.92<br>963.96	$P(4\text{-tolyl})Ph_2$<br>$[P(4\text{-tolyl})Ph_3]^+$<br>$Pd(C_6F_5)(OAc)(PPh_3)$<br>$Pd(C_6F_5)_2(PPh_3)_2$

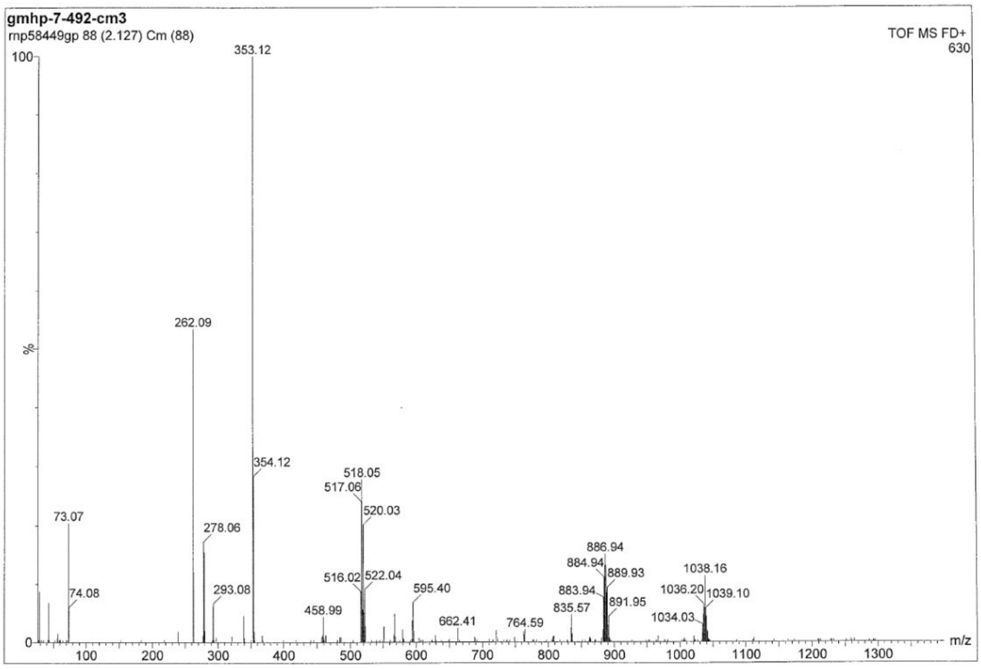

Figure S18. LIFDI-MS of a reaction aliquot collected from Scheme S1 after 6 min (5% conversion) **of** heating a mixture of **1**, **2a**, $Pd(OAc)_2$, PPh_3 and Ag_2CO_3 in DMF at 56 ± 1 °C.

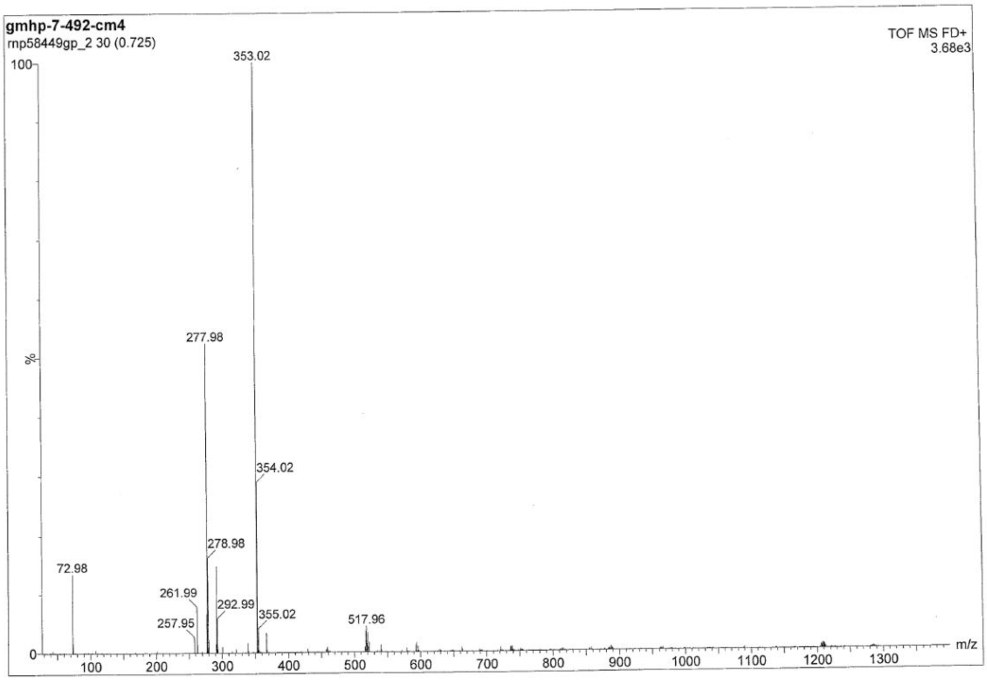

Figure S19. LIFDI-MS of an reaction aliquot collected from Scheme S1 after 47 min (60% conversion) of heating a mixture of **1**, **2a**, $Pd(OAc)_2$, PPh_3 and Ag_2CO_3 in DMF at 56 ± 1 °C**.**

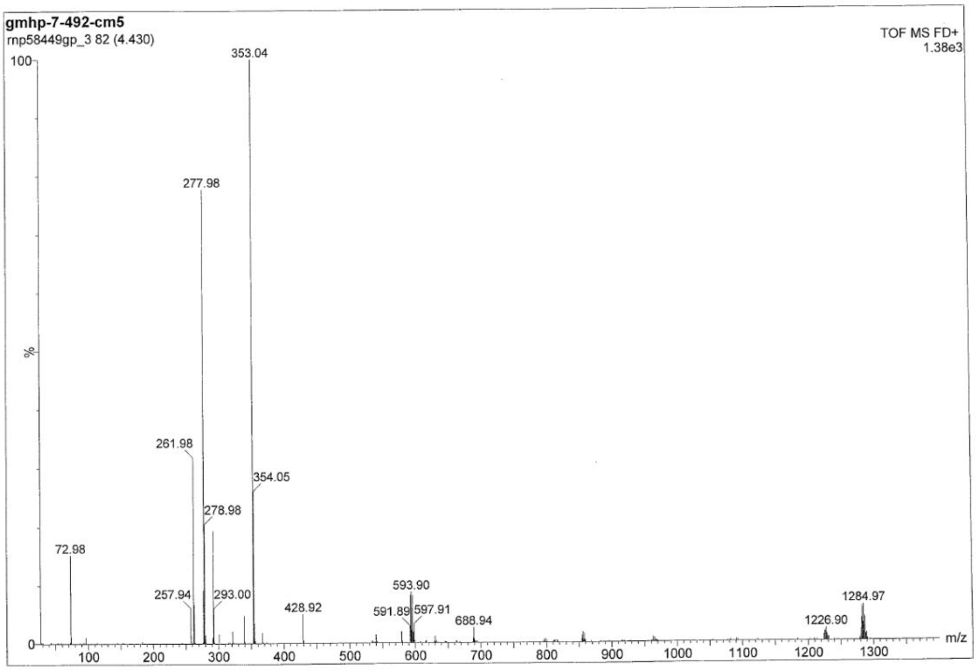

Figure S20. LIFDI-MS of an reaction aliquot collected from Scheme S1 after 86 min (quant. conversion) of heating a mixture of **1**, **2a**, $Pd(OAc)_2$, PPh_3 and Ag_2CO_3 in DMF at 56 ± 1 °C.

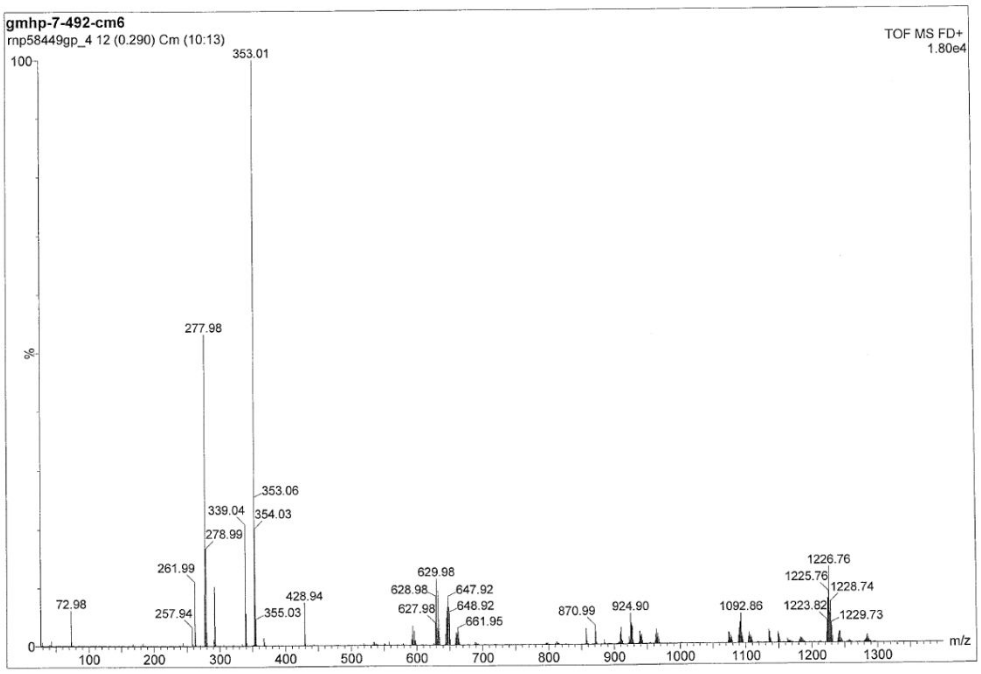

Figure S21. LIFDI-MS of an reaction aliquot collected from Scheme S1 after 126 min (quant. conversion) of heating a mixture of **1**, **2a**, $Pd(OAc)_2$, PPh_3 and Ag_2CO_3 in DMF at 56 ± 1 °C.

7. Reaction kinetics by in-situ IR spectroscopy

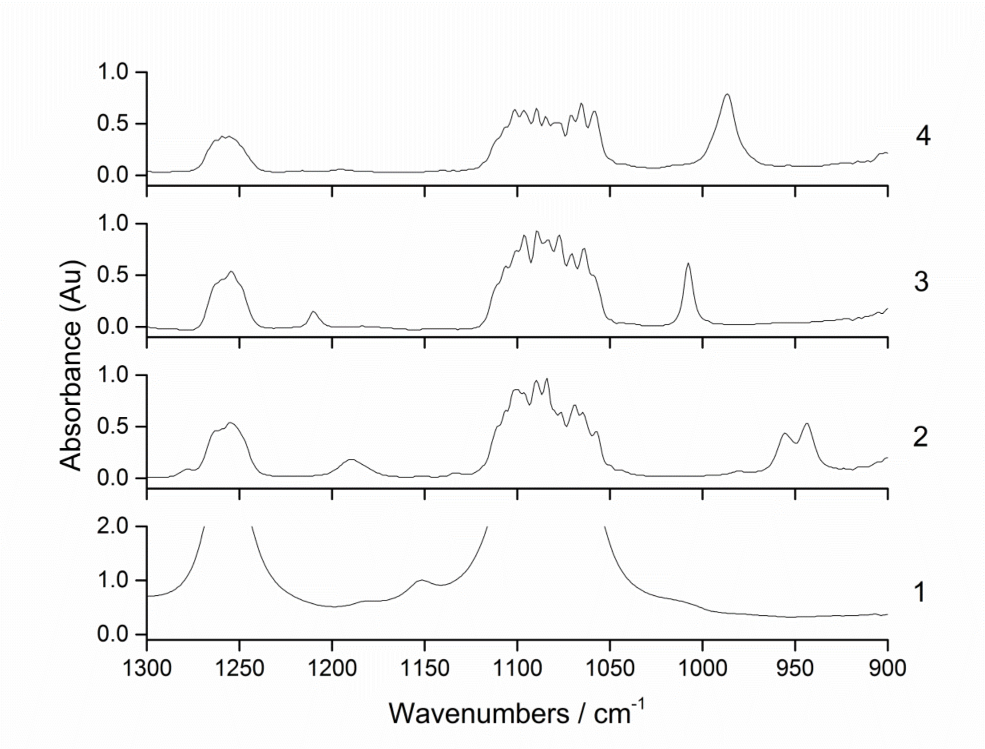

Figure S22. Overlaid FTIR spectra of (1) DMF, and DMF solutions of (2) pentafluorobenzene **1**, (3) 4-iodotoluene **2a** and (4) the product **3a** in the solution cell.

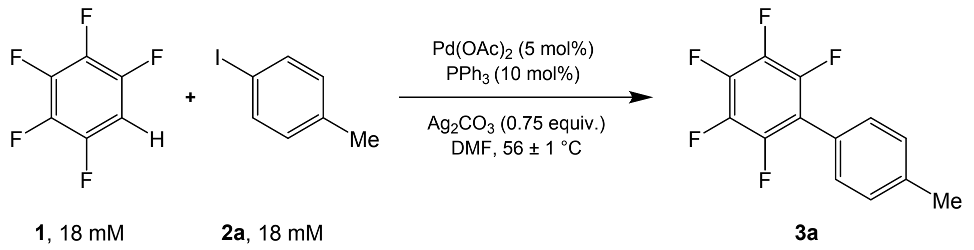

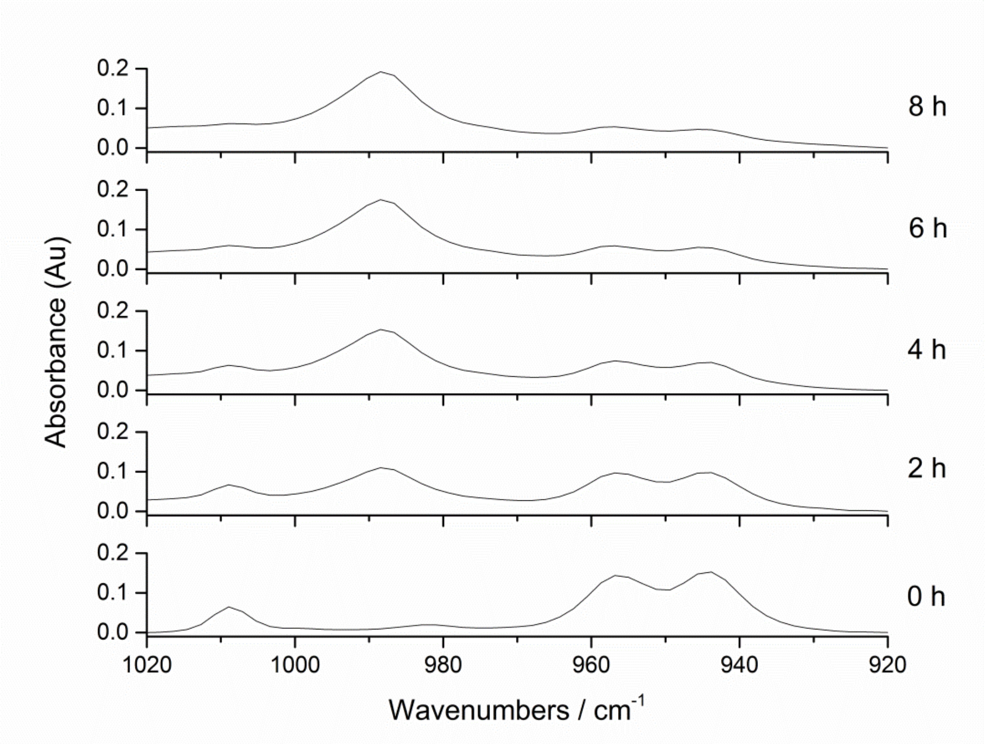

Figure S23. Stacked FTIR spectra of the reaction mixture shown in equation above above between 0–8 h reaction time generated by *in situ* FT-IR spectroscopic analysis. Absorbance observed for 4-iodotoluene **2a** (1009 cm^{-1}), the biaryl product **3a** (989 cm^{-1}) and pentafluorobenzene **1** (957 cm^{-1} and 944 cm^{-1}).

7.1 Variable Time Normalisation Analysis of the Direct Arylation of 4-Iodotoluene 2a with 1

Reaction under standard conditions

A mixture of 4-iodotoluene **2a** (197 mg, 117µL, 0.904 mmol, 1 equiv.), pentafluorobenzene **1** (150 µL, 227 mg, 1.35 mmol, 1.5 equiv.), $Pd(OAc)_2$ (10 mg [a], 0.045 mmol, 5 mol%), PPh_3 (24 mg [b], 0.091 mmol, 10 mol%) and Ag_2CO_3 (185 mg, 0.671 mmol, 0.74 equiv.) in DMF (9.4 cm^3 total volume) was prepared following general procedure C. The reaction progress was monitored by *in situ* FT-IR spectroscopy. Quantitative conversion of starting material **2a** was achieved.

[a] Using 500 µL of 0.09 M $Pd(OAc)_2$ stock solution made from stirring 20 mg $Pd(OAc)_2$ (0.089 mmol) in 1 cm^3 DMF for 10 min.

[b] Using 500 µL of 0.18 M PPh_3 stock solution made from stirring 47 mg PPh_3 (0.18 mmol) in 1 cm^3 DMF for 5 min.

(*Lab book reference number*: GMHP-7-474)

Example of reaction at different "excess" condition 1

A mixture of 4-iodotoluene **1a** (197 mg, 117 µL, 0.904 mmol, 1 equiv.), pentafluorobenzene **56** (100 µL, 151 mg, 0.901 mmol, 1 equiv.), $Pd(OAc)_2$ (10 mg [a], 0.045 mmol, 5 mol%), PPh_3 (24 mg [b], 0.091 mmol, 10 mol%) and Ag_2CO_3 (187 mg, 0.678 mmol, 0.75 equiv.) in DMF (9.45 cm^3 total volume) was prepared following general procedure C. The reaction progress was monitored by *in situ* FT-IR spectroscopy. Quantitative conversion of starting material **2a** was achieved.

[a] Using 500 µL of 0.09 M $Pd(OAc)_2$ stock solution made from stirring 20 mg $Pd(OAc)_2$ (0.089 mmol) in 1 cm^3 DMF for 10 min.

[b] Using 500 µL of 0.18 M PPh_3 stock solution made from stirring 47 mg PPh_3 (0.18 mmol) in 1 cm^3 DMF for 5 min.

(*Lab book reference number*: GMHP-7-477)

Example of reaction at different catalyst concentration 1

A mixture of 4-iodotoluene **2a** (197 mg, 117 µL, 0.904 mmol, 1 equiv.), pentafluorobenzene **56** (150 µL, 227 mg, 1.35 mmol, 1.5 equiv.), $Pd(OAc)_2$ (20 mg, 9 µL, 0.089 mmol, 10 mol%), PPh_3 (47 mg, 43 µL, 0.18 mmol, 20 mol%) and Ag_2CO_3 (186 mg, 0.675 mmol, 0.75 equiv.) in DMF (9.35 cm^3 total volume) was prepared following general procedure C. The reaction progress was monitored by *in situ* FT-IR spectroscopy. Quantitative conversion of starting material **2a** was achieved.

(*Lab book reference number*: GMHP-7-475)

Example of reaction at the same "excess"

A mixture of 4-iodotoluene **2a** (98 mg, 58 µL, 0.45 mmol, 1 equiv.), **1** (100 µL, 151 mg, 0.90 mmol, 2 equiv.), $Pd(OAc)_2$ (10 mg [a], 0.045 mmol, 5 mol%), PPh_3 (24 mg [b], 0.091 mmol, 10 mol%) and Ag_2CO_3 (186 mg, 0.675 mmol, 1.5 equiv.) in DMF (9.51 cm^3 total volume) was prepared following general procedure C. The reaction progress was monitored by *in situ* FT-IR spectroscopy. Quantitative conversion of starting material **2a** was achieved.

[a] Using 500 µL of 0.09 M $Pd(OAc)_2$ stock solution made from stirring 20 mg $Pd(OAc)_2$ (0.089 mmol) in 1 cm^3 DMF for 10 min.

[b] Using 500 µL of 0.18 M PPh_3 stock solution made from stirring 47 mg PPh_3 (0.18 mmol) in 1 cm^3 DMF for 5 min.

(*Lab book reference number*: GMHP-7-478)

7.2 Variable Time Normalisation Analysis

A more suitable method for analysing the conversion reaction profiles obtained by *in situ* FT-IR spectroscopy is the variable time normalisation analysis (VTNA). The kinetic data was treated by the normalised time scale method, where the time axis was replaced with the multiple of the reaction time and the initial concentration of the reaction component of interest raised to the nth-power. This allowed the kinetic contribution of the respective reaction components to be removed from the kinetic profiles. The overlapping kinetic profiles were achieved for first-order kinetics in pentafluorobenzene **56** (Figure S24), zeroth-order kinetics in 4-iodotoluene **57** (Figure S25) and 0.5-order kinetics in the Pd catalyst (**Figure S26**).

The VTNA approach, carried out with two different excess concentrations of **1**, showed the data overlapping best for zero-order with respect to [iodotoluene] and first order with respect to [pentafluorobenzene] (Figure S24). The best fit for the order in palladium catalyst (defined by the total palladium concentration) was investigated with two different catalyst concentrations (4.6 and 9.2 mM with corresponding changes in [PPh_3]) according to VTNA is 0.5, with a significantly poorer fit for an order of 0.75. The kinetics were followed with three same excess sets of concentrations: (a) the standard condition reaction with initial concentrations of 93 mM [Itol **2a**] and 140 mM [C_6F_5H **1**], (b) initial concentrations of 47 mM [**2a**] and 93 mM [**1**], (c) excess product added at the start, initial concentrations of 47 mM [**2a**], 93 mM [**1**], 47 mM [**3a**]. The resulting overall rate law is:

$$v(t) = +d[\mathbf{3a}]/dt = k_r[\mathbf{1}][Pd]_{TOT}^{1/2}$$

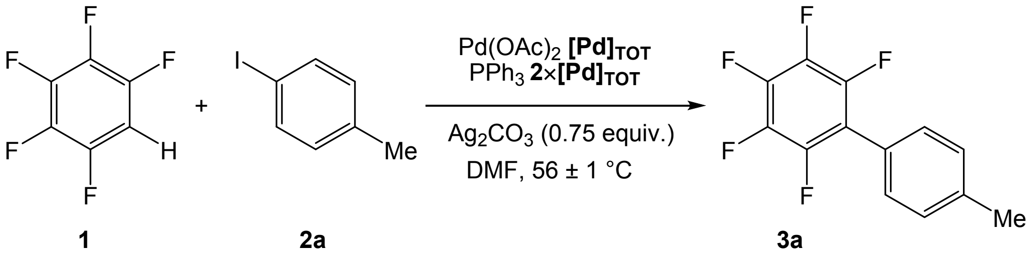

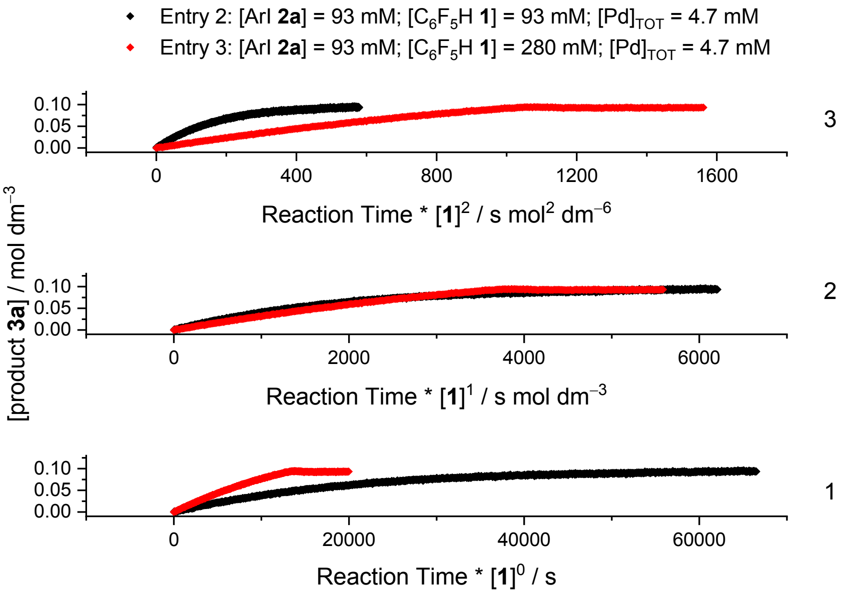

Figure S24. Kinetic profiles of the product **2a** formation for the reaction shown in scheme above, normalised to the concentration of pentafluorobenzene **1** for (1) zeroth-order, (2) first-order, and (3) second-order kinetics.

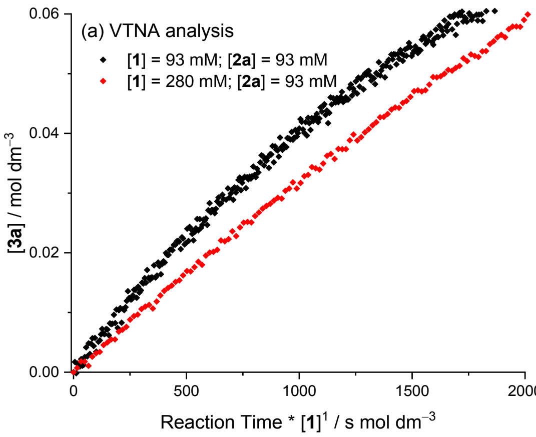

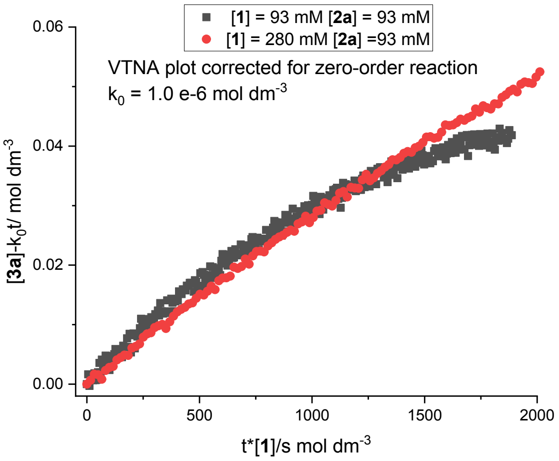

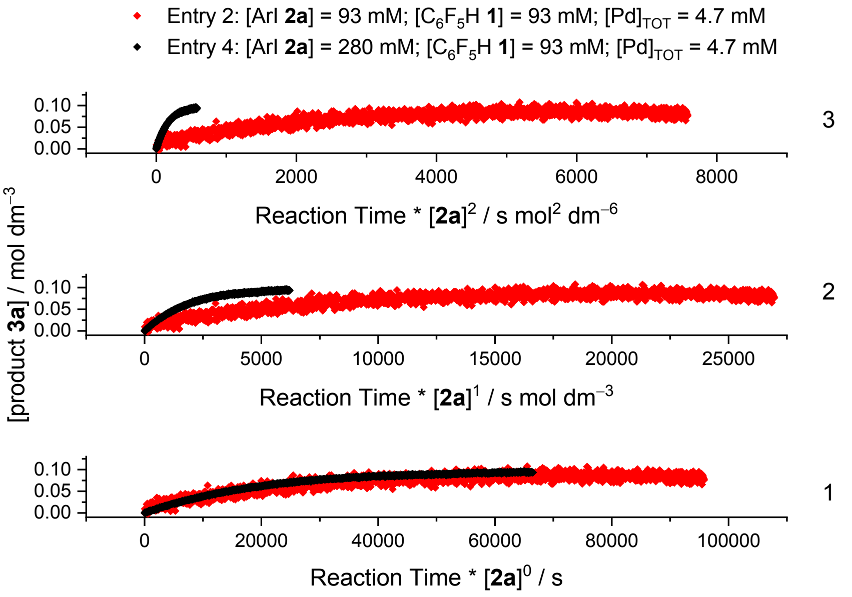

Figure S25. Kinetic profiles of the product **3a** formation for the reaction shown in scheme above, normalised to the concentration of 4-iodotoluene **2a** for (1) zeroth-order, (2) first-order, and (3) second-order kinetics.

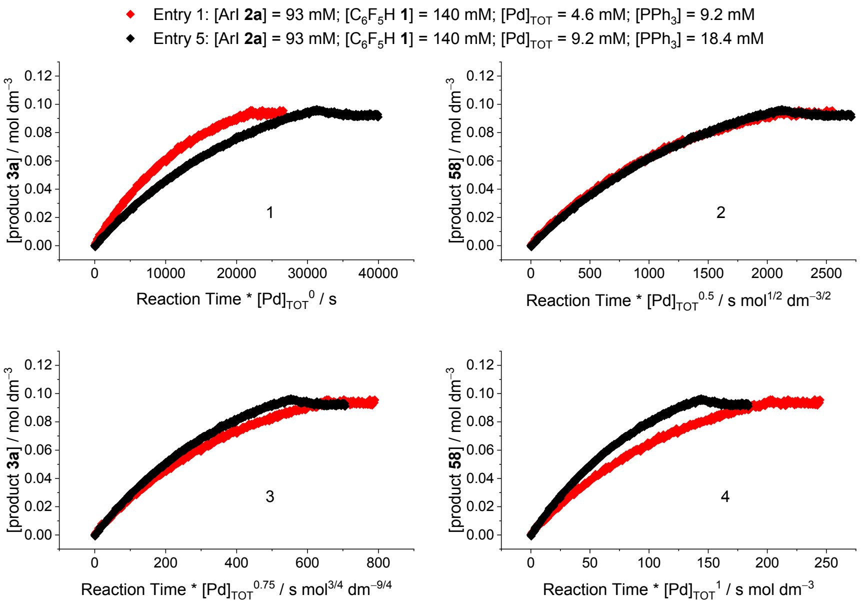

Figure S26. Kinetic profiles of the product **3a** formation for the reaction shown in scheme above, normalised to the concentration of the catalyst for **(1)** zeroth-order, **(2)** 0.5-order, **(3)** 0.75-order, and **(4)** first-order kinetics.

7.3 Kinetic Analysis of the Direct Arylation of 4-Iodotoluene 2a with 1 by Isolation Method

The traditional isolation method requires a large excess of reagent (either **1** or **2a**). With the same concentration of **2a** as that used for the VTNA analysis, a 10-fold excess of **1** would lead to a solution with 9:1 v/v of DMF to **1**. For this reason, the concentrations were reduced compared to the VTNA

experiments with the limiting reagent set at 18 mM, leading to reduced signal-to-noise compared to that for VTNA.

Order with respect to pentafluorobenzene 1 and 2a for $Pd(OAc)_2/PPh_3$ 1:2 pre-catalyst

A mixture of 4-iodotoluene **2a** (39 mg, 0.18 mmol, 1 equiv.), **1** (see below), $Pd(OAc)_2$ (2 mg [a], 8.9 × 10^{-6} mol, 5 mol%), PPh_3 (4.7 mg [b], 0.018 mmol, 10 mol%) and Ag_2CO_3 (37 mg, 0.13 mmol, 0.75 equiv.) in DMF (see below) was prepared following general procedure C. The reaction progress was monitored by *in situ* FT-IR spectroscopy.

1. Pentafluorobenzene **1** (0.2 cm^3, 1.8 mmol, 0.3 g, 10 equiv.), DMF (9.5 cm^3 total volume); Quantitative conversion of starting material **2a** was achieved with k_{obs} = (1.68 ± 0.02) × 10^{-6} mol dm^{-3} s^{-1}. (*Lab book reference number*: GMHP-4-233. The method was repeated with:
2. **1** (0.4 cm^3, 0.6 g, 3.6 mmol, 20 equiv.), DMF (9.3 cm^3 total volume)
3. **1** (0.6 cm^3, 0.9 g, 5.4 mmol, 30 equiv.), DMF (9.1 cm^3 total volume)
4. **1** (0.8 cm^3, 1.2 g, 7.2 mmol, 40 equiv.), DMF (8.9 cm^3 total volume)
5. **1** (1 cm^3, 1.5 g, 9 mmol, 50 equiv.), DMF (8.7 cm^3 total volume)

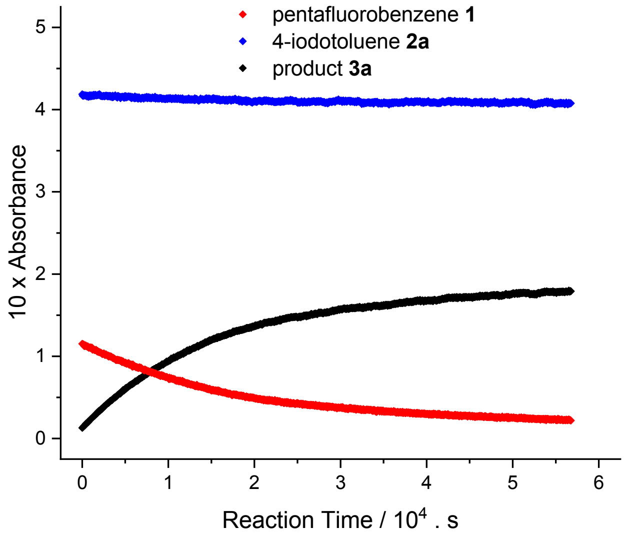

Figure S27. Isolation method of kinetic analysis with 10-fold excess of reagent **2a.**

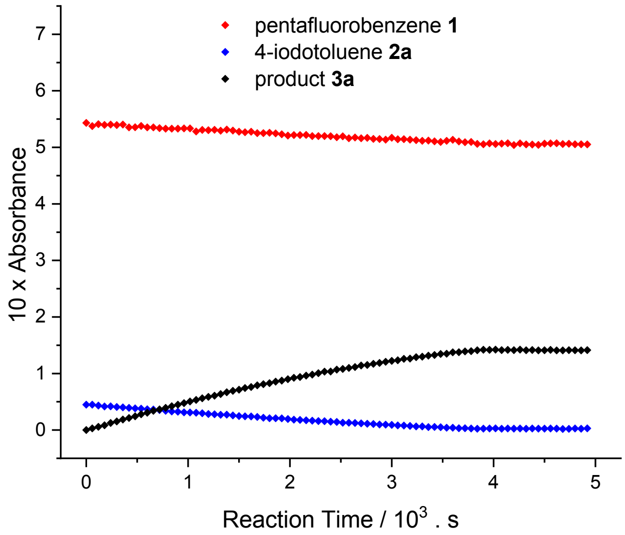

Figure S28. Isolation method of kinetic analysis with 10-fold excess of reagent **1.**

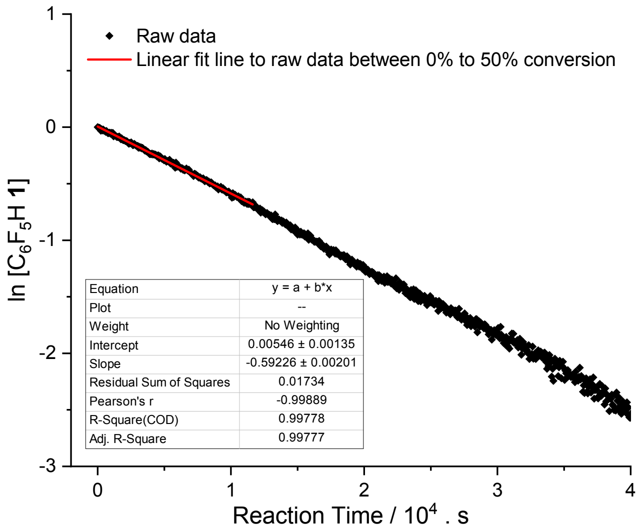

Figure S29. Absorbance decay of pentafluorobenzene **1** under pseudo-first-order conditions.

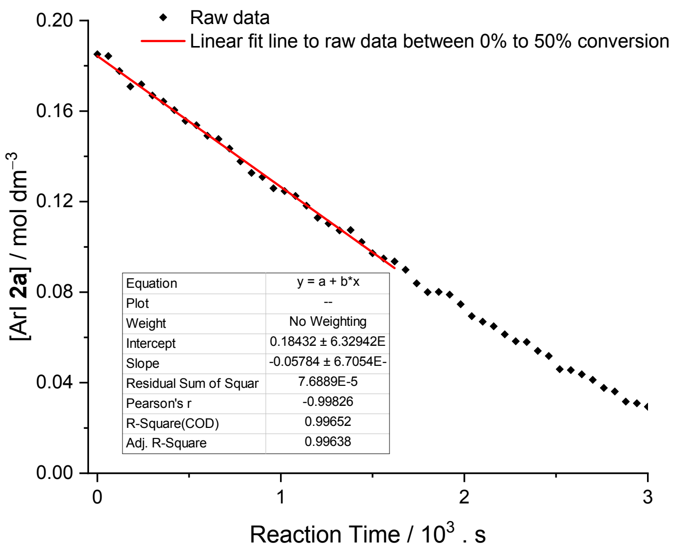

Figure S30. Absorbance decay of **2a** under pseudo-zeroth-order conditions

Table S14. Variation of k_{obs} with **[1]** for the direct arylation reaction of **2a** at 56 ± 1 °C.

Entry	Equivalents	[1] / mol dm^{-3}	k_{obs} / 10^{-6} . mol dm^{-3} s^{-1}
**1**	10	0.18	1.68 ± 0.02
**2**	20	0.36	2.06 ± 0.03
**3**	30	0.55	2.56 ± 0.04
**4**	40	0.73	3.19 ± 0.06
**5**	50	0.92	4.04 ± 0.07

Order with respect to Pd catalyst with $Pd(OAc)_2/PPh_3$ 1:2 pre-catalyst

A mixture of 4-iodotoluene **2a** (39 mg, 0.18 mmol, 1 equiv.), **1** (0.2 cm^3, 0.3 g, 1.8 mmol, 10 equiv.), $Pd(OAc)_2$ (see below), PPh_3 (see below) and Ag_2CO_3 (37 mg, 0.13 mmol, 0.75 equiv.) in DMF (9.5 cm^3 total volume) were prepared following the general procedure C. The reaction progress was monitored by *in situ* FT-IR spectroscopy.

1. $Pd(OAc)_2$ (0.4 mg [a], 1.8 × 10^{-6} mol, 1.0 mol%), PPh_3 (0.94 mg [b], 3.6 × 10^{-6} mol, 2.0 mol%). [a] Using 120 µL of 0.015 M $Pd(OAc)_2$ stock solution made from stirring 20 mg $Pd(OAc)_2$ (0.089 mmol) in 6 cm^3 DMF for 10 min. [b] Using 120 µL of 0.03 M PPh_3 stock solution made from stirring 47 mg PPh_3 (0.18 mmol) in 6 cm^3 DMF for 5 min; 77% conversion of starting material **2a** was achieved with k_{obs} = (3.52 ± 0.03) × 10^{-7} mol dm^{-3} s^{-1}. (*Lab book reference number*: GMHP-5-304). The method was repeated with:
2. $Pd(OAc)_2$ (1.0 mg, 4.5 × 10^{-6} mol, 2.5 mol%), PPh_3 (2.4 mg, 9.0 × 10^{-6} mol, 5.0 mol%).
3. $Pd(OAc)_2$ (2.0 mg, 9 × 10^{-6} mol, 5.0 mol%), PPh_3 (4.7 mg, 1.8 × 10^{-5} mol, 10 mol%)
4. $Pd(OAc)_2$ (4.0 mg, 0.018 mmol, 10 mol%), PPh_3 (9.4 mg, 0.036 mmol, 20 mol%).
5. $Pd(OAc)_2$ (8.1 mg, 0.036 mmol, 20 mol%), PPh_3 (18.9 mg, 0.072 mmol, 40 mol%).

Table S15. Variation of k_{obs} with $[Pd_{tot}/2PPh_3]$ for direct arylation of **2a** at 56 ± 1 °C (with **[1]** = 180 mM).

Entry	mol% Equiv.	$[Pd]_{TOT}$ / 10^{-3} . mol dm^{-3}	k_{obs} / 10^{-6} . mol dm^{-3} s^{-1}
**1**	1.0	0.19	0.352 ± 0.003
**2**	2.5	0.46	0.790 ± 0.006
**3**	5.0	0.92	1.69 ± 0.02
**4**	10	1.86	1.84 ± 0.04
**5**	20	3.70	3.61 ± 0.13

Table S16. Variation with k_{obs} for the direct arylation reaction of **2a** with **1** at varying ratio of $[Pd]:[PPh_3]$at 56 ± 1 °C.

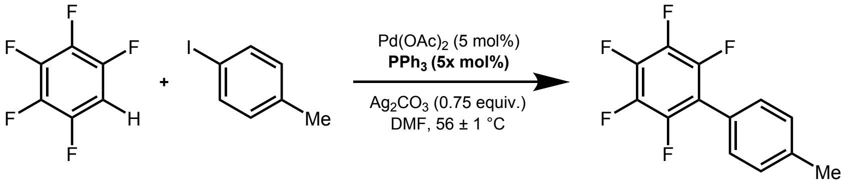

1, [0.18 M] 2a, [0.018 M] **3a**

Entry	PPh_3 quantity / mol%	$[PPh_3]$ / $[Pd(OAc)_2]$ [a]	k_{obs} / 10^{-6} . mol dm^{-3}
**1**	5	1.01	0.604 ± 0.007
**2**	10	2.00	1.68 ± 0.02
**3**	15	3.04	2.78 ± 0.03
**4**	20	4.00	3.73 ± 0.08
**5**	25	5.01	2.72 ± 0.05
**6**	30	6.12	2.67 ± 0.04
**7**	40	8.03	2.71 ± 0.06

[a] $[Pd(OAc)_2]$ = 0.93 mM

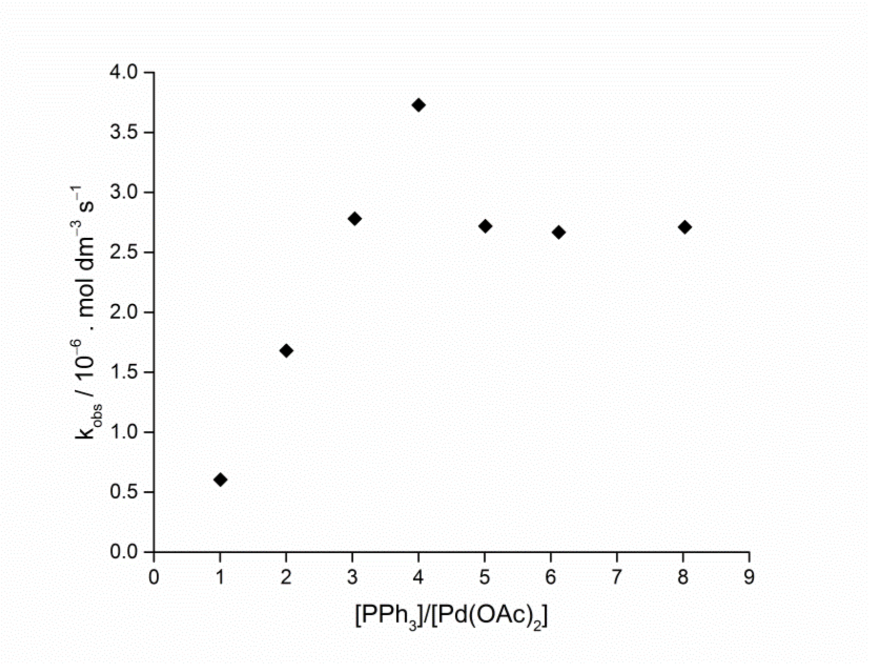

Figure S31. Plot of k_{obs} against $[PPh_3]/[Pd(OAc)_2]$ ratio for the reaction shown in Table S16 at 56 ± 1 °C.

Order with respect to 1 for $Pd(OAc)_2/PPh_3$ 1:4 pre-catalyst

A mixture of 4-iodotoluene **2a** (39 mg, 0.18 mmol, 1 equiv.), pentafluorobenzene **1** (see below), $Pd(OAc)_2$ (2 mg [a], 8.9 × 10^{-6} mol, 5 mol%), PPh_3 (9.4 mg [b], 0.036 mmol, 20 mol%) and Ag_2CO_3 (37 mg, 0.13 mmol, 0.75 equiv.) in DMF (see below) was prepared following general procedure C. The reaction progress was monitored by *in situ* FT-IR spectroscopy.

[a] Using 200 µL of 45 mM $Pd(OAc)_2$ stock solution made from stirring 20 mg $Pd(OAc)_2$ (0.089 mmol) in 2 cm^3 DMF for 10 min.

[b] Using 200 µL of 0.18 M PPh_3 stock solution made from stirring 47 mg PPh_3 (0.18 mmol) in 1 cm^3 DMF for 5 min.

1. Pentafluorobenzene **1** (0.2 cm^3, 0.3 g, 1.8 mmol, 10 equiv.), DMF (9.5 cm^3 total volume); Quantitative conversion of starting material **2a** was achieved with k_{obs} = (3.73 ± 0.08) × 10^{-6} mol dm^{-3} s^{-1}. (*Lab book reference number*: GMHP-6-349) The method was repeated with:
2. **1** (0.4 cm^3, 0.6 g, 3.6 mmol, 20 equiv.), DMF (9.3 cm^3 total volume)
3. **1** (0.6 cm^3, 0.9 g, 5.4 mmol, 30 equiv.), DMF (9.1 cm^3 total volume)
4. **1** (0.8 cm^3, 1.2 g, 7.2 mmol, 40 equiv.), DMF (8.9 cm^3 total volume)

Table S17. The variation of k_{obs} with $[Pd_{tot}/4PPh_3]$ for direct arylation of **2a** by **1** at 56 ± 1 °C.

Entry	$[Pd]_{TOT}$ / 10^{-3} mol dm^{-3}	k_{obs} / 10^{-6} . mol dm^{-3} s^{-1}
**1**	0.93	3.73 ± 0.08
**2**	1.87	5.96 ± 1.02
**3**	2.81	6.74 ± 0.84
**4**	3.70	9.91 ± 1.70

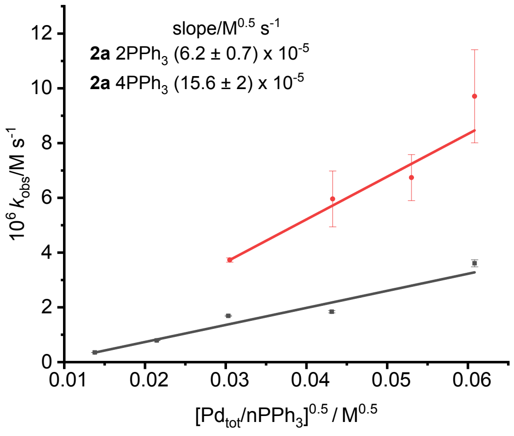

Figure S32. Plot of k_{obs} vs$[[Pd_{tot}/nPPh_3]^{0.5}$ for n = 2 and n = 4 for direct arylation of **2a** by **1** at 56 ± 1 °C.

7.4 Kinetic Analysis of the Direct Arylation of Iodobenzene 2b with 1 by Isolation Method

Order with respect to pentafluorobenzene 1

A mixture of iodobenzene **2b** (60 µL, 0.54 mmol, 1 equiv.), **1** (see below), $Pd(OAc)_2$ (6 mg [a], 0.027 mmol, 5 mol%), PPh_3 (14 mg [b], 0.054 mmol, 10 mol%) and Ag_2CO_3 (111 mg, 0.40 mmol) in DMF (see below) was prepared following general procedure C. The reaction progress was monitored by *in situ* FT-IR spectroscopy.

[a] Using 300 µL of 0.09 M $Pd(OAc)_2$ stock solution made from stirring 20 mg $Pd(OAc)_2$ (0.089 mmol) in 1 cm^3 DMF for 10 min.

[b] Using 300 µL of 0.18 M PPh_3 stock solution made from stirring 47 mg PPh_3 (0.18 mmol) in 1 cm^3 DMF for 5 min.

8. Pentafluorobenzene **1** (0.6 cm^3, 0.9 g, 5.4 mmol, 10 equiv.), DMF (9.1 cm^3 total volume); Quantitative conversion of starting material **3b** with k_{obs} = (9.99 ± 0.11) × 10^{-6} mol dm^{-3} s^{-1}. (*Lab book reference number*: GMHP-5-287)
9. **1** (1.2 cm^3, 1.8 g, 0.011 mol, 20 equiv.), DMF (8.5 cm^3 total volume)
10. **1** (1.8 cm^3, 2.7 g, 0.016 mol, 30 equiv.), DMF (7.9 cm^3 total volume)
11. **1** (2.4 cm^3, 3.6 g, 0.022 mol, 40 equiv.), DMF (7.3 cm^3 total volume)

Order with respect to Pd catalyst with $Pd(OAc)_2/PPh_3$ 1:2 pre-catalyst

A mixture of iodobenzene **2b** (60 µL, 0.54 mmol, 1 equiv.), **1** (0.6 cm^3, 0.9 g, 5.4 mmol, 10 equiv.), $Pd(OAc)_2$ (see below), PPh_3 (see below) and Ag_2CO_3 (111 mg, 0.40 mmol, 0.75 equiv.) in DMF (9.1 cm^3 total volume) was prepared following general procedure C. The reaction progress was monitored by *in situ* FT-IR spectroscopy.

1. $Pd(OAc)_2$ (6 mg [a], 0.027 mmol, 5 mol%), PPh_3 (14 mg [b], 0.054 mmol, 10 mol%). [a] Using 300 µL of 0.09 M $Pd(OAc)_2$ stock solution made from stirring 20 mg $Pd(OAc)_2$ (0.089 mmol) in 1 cm^3 DMF for 10 min. [b] Using 300 µL of 0.18 M PPh_3 stock solution made from stirring 47 mg PPh_3 (0.18 mmol) in 1 cm^3 DMF for 5 min); Quantitative conversion of starting material **2b** with k_{obs} = (9.99 ± 0.11) × 10^{-6} mol dm^{-3} s^{-1}. (*Lab book reference number*: GMHP-5-287). The method was repeated with:
2. $Pd(OAc)_2$ (12 mg, 0.053 mmol, 10 mol%), PPh_3 (28 mg, 0.11 mmol, 20 mol%)
3. $Pd(OAc)_2$ (24 mg, 0.11 mmol, 20 mol%), PPh_3 (56 mg, 0.22 mmol, 40 mol%)
4. $Pd(OAc)_2$ (37 mg, 0.16 mmol, 30 mol%), PPh_3 (85 mg, 0.32, 60 mol%)

Order with respect to Pd catalyst with $Pd(OAc)_2/PPh_3$ 1:4 pre-catalyst

A mixture of 4-iodotoluene **2b** (39 mg, 0.18 mmol, 1 equiv.), **1** (0.2 cm^3, 0.3 g, 1.8 mmol, 10 equiv.), $Pd(OAc)_2$ (see below), PPh_3 (see below) and Ag_2CO_3 (37 mg, 0.13 mmol, 0.75 equiv.) in DMF (9.5 cm^3 total volume) were prepared following the general procedure C. The reaction progress was monitored by *in situ* FT-IR spectroscopy.

1. $Pd(OAc)_2$ (2 mg [a], 9 × 10^{-6} mol, 5.0 mol%), PPh_3 (9.4 mg [b], 0.036 mmol, 20 mol%). [a] Using 200 µL of 45 mM $Pd(OAc)_2$ stock solution made from stirring 20 mg $Pd(OAc)_2$ (0.089 mmol) in 2 cm^3 DMF for 10 min. [b] Using 400 µL of 90 mM PPh_3 stock solution made from stirring 47 mg PPh_3 (0.18 mmol) in 2 cm^3 DMF for 5 min; Quantitative conversion of starting material **2a** was achieved with k_{obs} = (3.73 ± 0.08) × 10^{-6} mol dm^{-3} s^{-1}. (*Lab book reference number*: GMHP-6-349). The method was repeated with:
2. $Pd(OAc)_2$ (4 mg, 0.018 mmol, 10 mol%), PPh_3 (19 mg, 0.073 mmol, 40 mol%).
3. $Pd(OAc)_2$ (6 mg 0.027 mmol, 15 mol%), PPh_3 (28 mg, 0.11 mmol, 60 mol%).
4. $Pd(OAc)_2$ (8.1 mg, 0.036 mmol, 20 mol%), PPh_3 (38 mg, 0.14 mmol, 0.8 equiv.).

Table S18. Variation of rate with [**1**] for the direct arylation reaction of **2b** at 56 ± 1 °C

Entry	Equivalents	$[1]_{ave}$ / mol dm^{-3}	k_{obs} / 10^{-5} . mol dm^{-3} s^{-1}
**1**	10	0.53	0.999 ± 0.011
**2**	20	1.08	1.24 ± 0.02
**3**	30	1.63	1.57 ± 0.02
**4**	40	2.19	2.07 ± 0.05

Table S19. Variation of rate with $[Pd]_{tot}$ for the direct arylation reaction of **2b** with **1** at 56 ± 1 °C

Entry	mol% Equiv.	$[Pd]_{TOT}$ / 10^{-3} . mol dm^{-3}	k_{obs} / 10^{-5} . mol dm^{-3} s^{-1}
**1**	5	2.79	0.999 ± 0.011
**2**	10	5.51	1.61 ± 0.01
**3**	20	11.0	2.18 ± 0.03
**4**	30	16.7	2.41 ± 0.04

7.5 Kinetic Analysis of the Direct Arylation of 4-Iodotoluene 2b with 1 using Different Quantity of PPh_3

A mixture of 4-iodotoluene **2b** (39 mg, 0.18 mmol, 1 equiv.), pentafluorobenzene **1** (200 µL, 0.3 g, 1.8 mmol, 10 equiv.), $Pd(OAc)_2$ (2 mg [a], 8.9 × 10^{-6} mol, 5 mol%), PPh_3 (see below) and Ag_2CO_3 (37 mg, 0.13 mmol, 0.75 equiv.) in DMF (9.5 cm^3 total volume) was prepared following general procedure C. The reaction progress was monitored by *in situ* FT-IR spectroscopy.

[a] Using 200 µL of 45 mM $Pd(OAc)_2$ stock solution made from stirring 20 mg $Pd(OAc)_2$ (0.089 mmol) in 2 cm^3 DMF for 10 min.

1. PPh_3 (2.4 mg [b], 0.009 mmol, 5 mol%), [b] Using 100 µL of 90 mM PPh_3 stock solution made from stirring 47 mg PPh_3 (0.18 mmol) in 2 cm^3 DMF for 5 min.; 76% conversion with k_{obs} = (6.04 ± 0.07) × 10^{-7} mol dm^{-3} s^{-1}. (*Lab book reference number*: GMHP-6-348)
2. PPh_3 (4.7 mg, 0.018 mmol, 10 mol%), Quantitative conversion with k_{obs} = (1.68 ± 0.02) × 10^{-6} mol dm^{-3} s^{-1}. (*Lab book reference number*: GMHP-4-233)
3. PPh_3 (7.1 mg, 0.027 mmol, 15 mol%), Quantitative conversion of with k_{obs} = (2.78 ± 0.03) × 10^{-6} mol dm^{-3} s^{-1}. (*Lab book reference number*: GMHP-6-367)
4. PPh_3 (9.4 mg, 0.036 mmol, 20 mol%), Quantitative conversion with k_{obs} = (3.73 ± 0.08) × 10^{-6} mol dm^{-3} s^{-1}. (*Lab book reference number*: GMHP-6-349)
5. PPh_3 (12 mg, 0.046 mmol, 25 mol%), Quantitative conversion with k_{obs} = (2.72 ± 0.05) × 10^{-6} mol dm^{-3} s^{-1}. (*Lab book reference number*: GMHP-6-380)
6. PPh_3 (14 mg, 0.054 mmol, 30 mol%), Quantitative conversion with k_{obs} = (2.67 ± 0.04) × 10^{-6} mol dm^{-3} s^{-1} (*Lab book reference number*: GMHP-6-357)
7. PPh_3 (19 mg, 0.072 mmol, 40 mol%), Quantitative conversion with k_{obs} = (2.71 ± 0.06) × 10^{-6} mol dm^{-3} s^{-1}. (*Lab book reference number*: GMHP-6-350)

Table S20. Variation of rate with **[1]** for the direct arylation reaction of **2b** at 56 ± 1 °C.

Entry	Equivalents	$[1]_{ave}$ / mol dm^{-3}	k_{obs} / 10^{-5} mol dm^{-3} s^{-1}
**1**	10	0.53	0.999 ± 0.011
**2**	20	1.08	1.24 ± 0.02
**3**	30	1.63	1.57 ± 0.02
**4**	40	2.19	2.07 ± 0.05

Table S21. Variation of rate with $[Pd_{tot}/2PPh_3]$ for the direct arylation reaction of **2b** with **1** at 56 ± 1 °C.

Entry	mol% Equiv.	$[Pd]_{TOT}$ / 10^{-3} mol dm^{-3}	k_{obs} / 10^{-5} mol dm^{-3} s^{-1}
**1**	5	2.79	0.999 ± 0.011
**2**	10	5.51	1.61 ± 0.01
**3**	20	11.0	2.18 ± 0.03
**4**	30	16.7	2.41 ± 0.04

7.6 Kinetic Analysis of the Direct Arylation of 4-Substituted-Iodobenzenes with 1

A mixture of 4-substituted-iodoarene (see below), pentafluorobenzene **1** (200 µL, 0.3 g, 1.8 mmol, 10 equiv.), $Pd(OAc)_2$ (2 mg [a], 8.9 × 10^{-6} mol, 5 mol%), PPh_3 (4.7 mg [b], 0.018 mmol, 10 mol%) and Ag_2CO_3 (37 mg, 0.13 mmol, 0.75 equiv.) in DMF (9.5 cm^3 total volume) was prepared following general procedure C. The reaction progress was monitored by *in situ* FT-IR spectroscopy.

[a] Using 200 µL of 45 mM $Pd(OAc)_2$ stock solution made from stirring 20 mg $Pd(OAc)_2$ (0.089 mmol) in 2 cm^3 DMF for 10 min.

[b] Using 200 µL of 90 mM PPh_3 stock solution made from stirring 47 mg PPh_3 (0.18 mmol) in 2 cm^3 DMF for 5 min.

1. OH (39 mg, 0.18 mmol, 1 equiv.); 0% conversion. (*Lab book reference number*: GMHP-7-510)
2. NH_2 (39 mg, 0.18 mmol, 1 equiv.); 0% conversion. (*Lab book reference number*: GMHP-7-500)
3. Me (39 mg, 0.18 mmol, 1 equiv.); Quantitative conversion with k_{obs} = (1.68 ± 0.04) × 10^{-6} mol dm^{-3} s^{-1}. (*Lab book reference number*: GMHP-4-233)
4. H (20 µL, 36 mg, 0.18 mmol, 1 equiv.); Quantitative conversion with k_{obs} = (1.01 ± 0.03) × 10^{-6} mol dm^{-3} s^{-1}. (*Lab book reference number*: GMHP-5-276)
5. F (21 µL, 40 mg, 0.18 mmol, 1 equiv.); Quantitative conversion with k_{obs} = (1.68 ± 0.04) × 10^{-6} mol dm^{-3} s^{-1}. (*Lab book reference number*: GMHP-7-501)
6. CF_3 (27 µL, 49 mg, 0.18 mmol, 1 equiv.); Quantitative conversion of starting material to product with k_{obs} = (1.13 ± 0.03) × 10^{-6} mol dm^{-3} s^{-1}. (*Lab book reference number*: GMHP-5-285)

The electronic effect of the iodoarene (ArI) was studied by comparing the k_{obs} obtained for the direct arylation reactions of different 4-substituted-iodoarenes with 10-fold excess pentafluorobenzene **1** under pseudo-zeroth-order kinetics condition at 56 ± 1 °C (Table S22). The k_{obs} values were calculated from 5–50% formation of the products determined by following the IR bands of the respective products around 990 cm^{-1}.

Table S22. k_{obs} for the direct arylation of substituted 4-iodoarenes with **1** at 56 ± 1 °C.

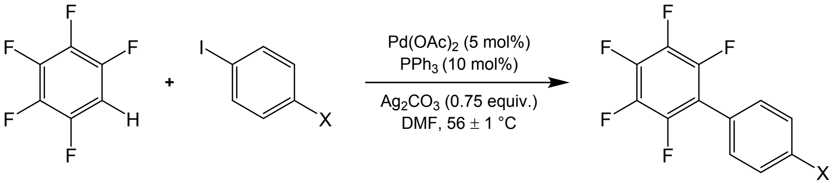

1, [0.18 M] [0.018 M] X = OH, NH$_2$, Me, H, F, CF$_3$

Entry	X	σ_{para}	k_{obs} [a] / 10^{-6} mol dm^{-3} s^{-1}
**1**	OH	−0.30	N/A
**2**	NH_2	−0.38	N/A
**3**	Me	−0.14	1.68 ± 0.04
**4**	H	0.00	1.01 ± 0.03
**5**	F	+0.15	1.68 ± 0.04
**6**	CF_3	+0.53	1.13 ± 0.03

[a] k_{obs} calculated from 5–50% formation of the products

7.7 Kinetic analysis with alternative catalysts

Table S23. Rates of direct arylation reaction of **1** with **2a** at 50 ± 1 °C using Ag_2CO_3 or Ag_2O additive with $Pd(OAc)_2$ or $Pd(PPh_3)_4$ pre-catalysts.

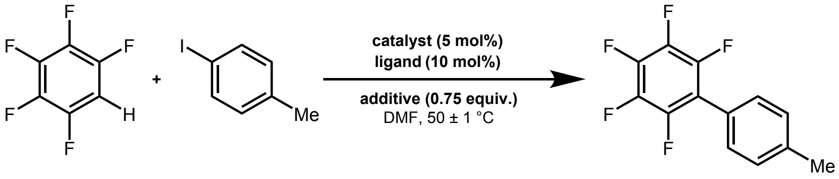

1 [1.8 M] **2a**, [0.18 M] 3a, quant.

Entry	Catalyst	Ligand	Additive	k_{obs} / 10^{-5} . mol dm^{-3} s^{-1}
**1**	$Pd(OAc)_2$	PPh_3	Ag_2CO_3	3.05 ± 0.03
**2**			Ag_2O	3.78 ± 0.03
**3**	$Pd(PPh_3)_4$	N/A	Ag_2CO_3	9.12 ± 0.04
**4**			Ag_2O	0.514 ± 0.002

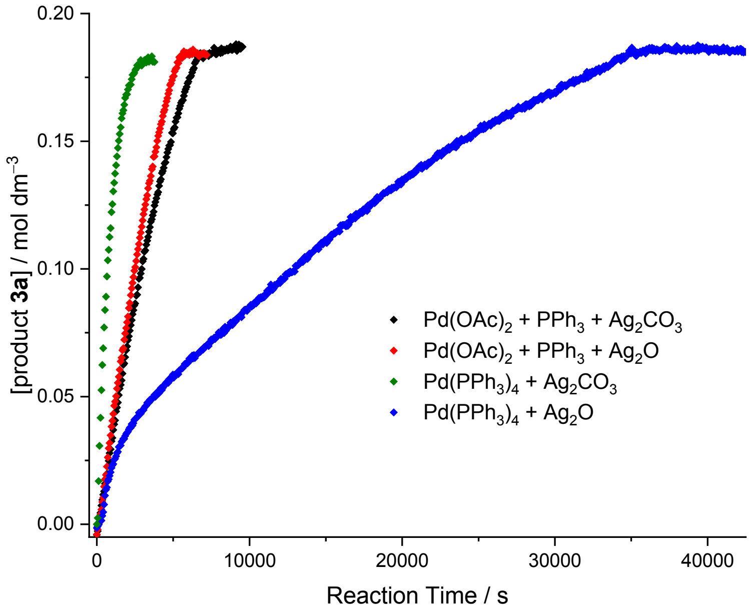

Figure S33. Reaction profiles of product **3a** formation for the reactions shown in Table S23.

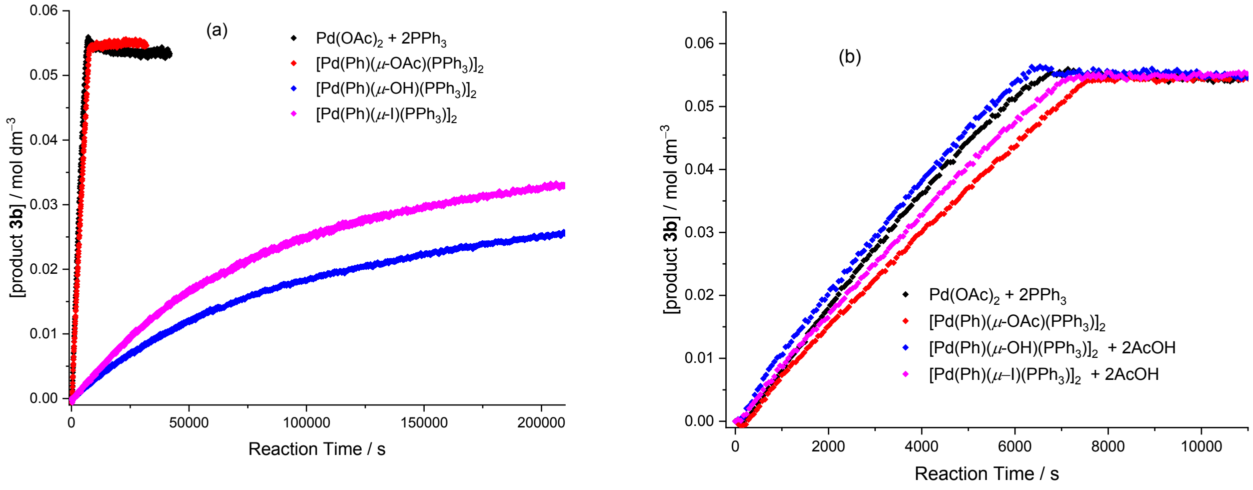

Figure S34. Kinetic profiles showing the product **3b** formation for the reactions catalysed by (a) the dinuclear complexes and (b) the dinuclear complexes with added AcOH.

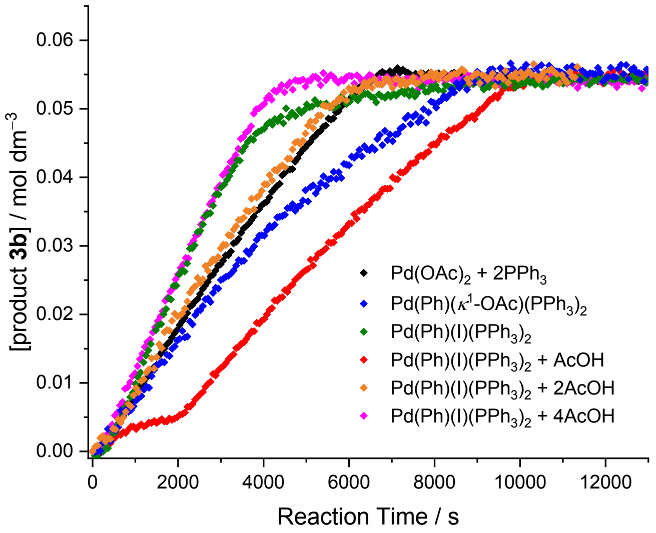

Figure S35. Kinetic profiles showing the product **3b** formation for the reactions catalysed by 5 mol% of mononuclear Pd species.

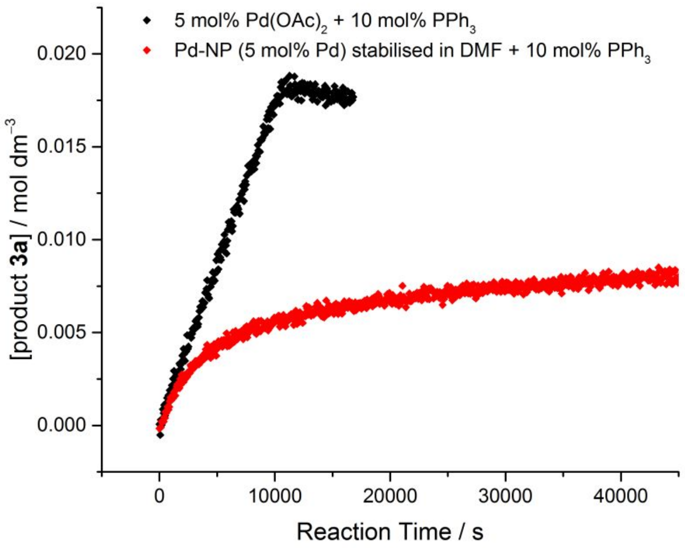

Figure S36. Reaction profile of direct arylation reaction catalyzed by (black) $Pd(OAc)_2$ pre-catalyst and (red) Pd-NPs stabilised in DMF at 56 ± 1 °C. Concentrations for black points: **1** (185 mM), **2a** (18.5 mM), $Pd(OAc)_2$ (0.93 mM), PPh_3 (1.85 mM), with Ag_2CO_3 (0.75 equiv) in DMF. Concentrations for red points: **1** (191 mM), **2a** (19.2 mM), Pd-NP (1.0 mM), PPh_3 (1.99 mM), with Ag_2CO_3 (0.75 equiv) in DMF.

7.8 Kinetic Isotope Effect of the Direct Arylation of 4-Iodotoluene 2b with Deuteropentafluorobenzene 1-d

A mixture of 4-iodotoluene **2a** (39 mg, 0.18 mmol, 1 equiv.), deuteropentafluorobenzene **1-d** (see below), $Pd(OAc)_2$ (2 mg [a], 8.9 × 10^{-6} mol, 5 mol%), PPh_3 (4.7 mg [b], 0.018 mmol, 10 mol%) and Ag_2CO_3 (37 mg, 0.13 mmol, 0.75 equiv.) in DMF (9.5 cm^3 total volume) was prepared following general procedure C. The reaction progress was monitored by *in situ* FT-IR spectroscopy. The IR signals of product **3a** at 989 cm^{-1} was monitored and the k_{obs} were used to calculate the KIE values.

[a] Using 200 µL of 45 mM $Pd(OAc)_2$ stock solution made from stirring 20 mg $Pd(OAc)_2$ (0.089 mmol) in 2 cm^3 DMF for 10 min.

[b] Using 200 µL of 90 mM PPh_3 stock solution made from stirring 47 mg PPh_3 (0.18 mmol) in 2 cm^3 DMF for 5 min.

Reactions heated at 40 ± 1 °C

1. C_6F_5D **1-d** (0.2 cm^3, 0.3 g, 1.8 mmol, 10 equiv.); with k_{obs} = (7.65 ± 0.02) × 10^{-8} mol dm^{-3} s^{-1}. (*Lab book reference number*: GMHP-6-340)
2. C_6F_5D **1-d** (0.8 cm^3, 1.2 g, 7.2 mmol, 40 equiv.); Quantitative conversion with k_{obs} = (3.20 ± 0.02) × 10^{-7} mol dm^{-3} s^{-1} (Entry 4). (*Lab book reference number*: GMHP-6-339)

Similarly at 56 ± 1 °C

The KIE calculated from the ratio of $k_{obs(H)}$ to $k_{obs(D)}$ was 4.36 ± 0.06 at 56 ± 1 °C

7.9 Temperature Dependence of the Direct Arylation of 4-Iodotoluene 2a with 1

A mixture of 4-iodotoluene **2a** (39 mg, 0.18 mmol, 1 equiv.), pentafluorobenzene **1** (see below), $Pd(OAc)_2$ (2 mg [a], 8.9 × 10^{-6} mol, 5 mol%), PPh_3 (4.7 mg [b], 0.018 mmol, 10 mol%) and Ag_2CO_3 (37 mg, 0.13 mmol, 0.75 equiv.) in DMF (see below) was prepared following general procedure C. The reaction progress was monitored by *in situ* FT-IR spectroscopy.

[a] Using 200 µL of 45 mM $Pd(OAc)_2$ stock solution made from stirring 20 mg $Pd(OAc)_2$ (0.089 mmol) in 2 cm^3 DMF for 10 min.

[b] Using 200 µL of 90 mM PPh_3 stock solution made from stirring 47 mg PPh_3 (0.18 mmol) in 2 cm^3 DMF for 5 min.

Reactions heated at 40.0 ± 1 °C

1. **1** (0.2 cm^3, 0.3 g, 1.8 mmol, 10 equiv.), DMF (9.5 cm^3 total volume); Quantitative conversion with k_{obs} = (5.22 ± 0.04) × 10^{-7} mol dm^{-3} s^{-1}. (*Lab book reference number*: GMHP-4-241)

2. **1** (0.4 cm^3, 0.6 g, 3.6 mmol, 20 equiv.), DMF (9.3 cm^3 total volume); Quantitative conversion with k_{obs} = (6.53 ± 0.06) × 10^{-7} mol dm^{-3} s^{-1}. (*Lab book reference number*: GMHP-5-327)
3. **1** (0.6 cm^3, 0.9 g, 5.4 mmol, 30 equiv.), DMF (9.1 cm^3 total volume); Quantitative conversion with k_{obs} = (8.60 ± 0.06) × 10^{-7} mol dm^{-3} s^{-1}. (*Lab book reference number*: GMHP-6-396)
4. **1** (0.8 cm^3, 1.2 g, 7.2 mmol, 40 equiv.), DMF (8.9 cm^3 total volume); Quantitative conversion with k_{obs} = (1.04 ± 0.02) × 10^{-6} mol dm^{-3} s^{-1}. (*Lab book reference number*: GMHP-5-333)
5. **1** (1 cm^3, 1.5 g, 9 mmol, 50 equiv.), DMF (8.7 cm^3 total volume); Quantitative conversion with k_{obs} = (1.27 ± 0.01) × 10^{-6} mol dm^{-3} s^{-1}. (*Lab book reference number*: GMHP-5-335)7

Similarly with reactions heated at 50 ± 1 °C, 64 ± 1 °C, 72 ± 1 °C

Table S24. Activation parameters calculated from experimentally determined $k_2(2K_D)^{-1/2}$ and k′ for half-order kinetics in Pd ($[Pd]_{TOT}$ = 0.9 mM).

Activation parameter	$k_2(2K_D)^{-1/2}$	k′
E_a [a] / kJ mol^{-1}	61.9 ± 2.6	60.9 ± 3.0
A [a] / s^{-1}	6.59 × 10^5	1.51 ×1 0^5
ΔH‡ [b] / J mol^{-1}	59.1 ± 2.6	58.2 ± 3.0
ΔS‡ [b] / J K^{-1} mol^{-1}	−143 ± 8	−155 ± 9
ΔG(298 K)‡ [b] / J mol^{-1}	102 ± 5	104 ± 6

[a] Values determined from Arrhenius plot. [b] Value determined from Eyring plot.

8. References

(1) Chaignon, N. M.; Fairlamb, I. J. S.; Kapdi, A. R.; Taylor, R. J. K.; Whitwood, A. C., Bis(triphenylphosphine)palladium(II)phthalimide - an easily prepared precatalyst for efficient Suzuki-Miyaura coupling of aryl bromides. *J. Mol. Catal. A Chem.* **2004,** *219*, 191–199.
(2) Coulson, D. R.; Satek, L. C.; Grim, S. O., Tetrakis(triphenylphosphine)palladium(0). In *Inorg. Synth.*, John Wiley & Sons, Inc.2007; pp 121–124.
(3) Grushin, V. V.; Alper, H., Alkali-induced disproportionation of palladium(II) tertiary phosphine complexes, [L_2PdCl_2], to L0 and palladium(0). Key intermediates in the biphasic carbonylation of ArX catalyzed by [L_2PdCl_2]. *Organometallics* **1993,** *12*, 1890–1901.
(4) Grushin, V. V.; Bensimon, C.; Alper, H., The first isolable organopalladium formato complexes - synthesis, characterization, and x-ray structure - facile and convenient thermal generation of coordinatively unsaturated palladium(0) species. *Organometallics* **1995,** *14*, 3259–3263.
(5) Negishi, E.; Takahashi, T.; Baba, S.; Vanhorn, D. E.; Okukado, N., Nickel-catalyzed or palladium-catalyzed cross coupling .31. Palladium-catalyzed or nickel-catalyzed reactions of alkenylmetals with unsaturated organic halides as a selective route to arylated alkenes and conjugated dienes - scope, limitations, and mechanism. *J. Am. Chem. Soc.* **1987,** *109*, 2393–2401.
(6) Burns, M. J.; Thatcher, R. J.; Taylor, R. J. K.; Fairlamb, I. J. S., Pd-catalysed regioselective C-H functionalisation of 2-pyrones. *Dalton Trans.* **2010,** *39*, 10391–10400.
(7) Tyrra, W.; Wickleder, M. S., Silver compounds in synthetic chemistry. 1 - A facile preparative route for pentafluorophenylsilver, AgC_6F_5 and its use as an oxidative pentafluorophenyl group transfer

reagent in reactions with group 12 to 16 elements - the single crystal structure of AgC_6F_5 . EtCN, the first arylsilver derivative crystallising in infinite chains. *Z. Anorg. Allg. Chem.* **2002,** *628*, 1841-1847.
(8) Edwards, D. A.; Harker, R. M.; Mahon, M. F.; Molloy, K. C., Aerosol-assisted chemical vapour deposition (AACVD) of silver films from triorganophosphine adducts of silver carboxylates, including the structure of $[Ag(O_2CC_3F_7)(PPh_{3)2}]$. *Inorg. Chim. Acta* **2002,** *328*, 134-146.
(9) Bowmaker, G. A.; Effendy; Hanna, J. V.; Healy, P. C.; King, S. P.; Pettinari, C.; Skelton, B. W.; White, A. H., Solution and mechanochemical syntheses, and spectroscopic and structural studies in the silver(I) (bi-)carbonate: triphenylphosphine system. *Dalton Trans.* **2011,** *40*, 7210-7218.
(10) Chen, F.; Min, Q. Q.; Zhang, X. G., Pd-Catalyzed direct arylation of polyfluoroarenes on water under mild conditions using PPh_3 ligand. *J. Org. Chem.* **2012,** *77*, 2992-2998.
(11) Cho, C. H.; Sun, M.; Seo, Y. S.; Kim, C. B.; Park, K., Nickel-catalyzed cross-coupling of neopentyl arenesulfonates with methyl and primary alkylmagnesium bromides. *J. Org. Chem.* **2005,** *70*, 1482–1485.
(12) Park, B. R.; Kim, K. H.; Kim, T. H.; Kim, J. N., Palladium-catalyzed benzoin-mediated redox process leading to biaryls from aryl halides. *Tetrahedron Lett.* **2011,** *52*, 4405–4407.
(13) Oldham, P. H.; Williams, G. H.; Wilson, B. A., Homolytic reactions of perfluoroaromatic compounds .4. formation of pentafluorophenyl radicals from pentafluoroaniline, and their reactions with aromatic compounds. *J. Chem. Soc. C* **1971**, 1094–1098.
(14) Korenaga, T.; Kosaki, T.; Fukumura, R.; Ema, T.; Sakai, T., Suzuki-Miyaura coupling reaction using pentafluorophenylboronic acid. *Org. Lett.* **2005,** *7*, 4915–4917.
(15) Lafrance, M.; Rowley, C. N.; Woo, T. K.; Fagnou, K., Catalytic intermolecular direct arylation of perfluorobenzenes. *J. Am. Chem. Soc.* **2006,** *128*, 8754–8756.
(16) Chen, F.; Min, Q. Q.; Zhang, X., Pd-catalyzed direct arylation of polyfluoroarenes on water under mild conditions using PPh3 ligand. *J. Org. Chem.* **2012,** *77*, 2992–2998.
(17) Johnson, S. A.; Taylor, E. T.; Cruise, S. J., A Combined Experimental and Computational Study of Unexpected C-F Bond Activation Intermediates and Selectivity in the Reaction of Pentafluorobenzene with a $(PEt_3)_2Ni$ Synthon. *Organometallics* **2009,** *28*, 3842–3855.
(18) Burdon, J.; Hollyhea.Wb; Tatlow, J. C., Aromatic polyfluoro-compounds .29. nucleophilic replacement reactions of pentafluorobenzoic acid. *Journal of the Chemical Society* **1965**, 6336–6342.
